# Molecular pathway activation features linked with transition from normal skin to primary and metastatic melanomas in human

**DOI:** 10.18632/oncotarget.6394

**Published:** 2015-11-26

**Authors:** Denis Shepelin, Mikhail Korzinkin, Anna Vanyushina, Alexander Aliper, Nicolas Borisov, Raif Vasilov, Nikolay Zhukov, Dmitry Sokov, Vladimir Prassolov, Nurshat Gaifullin, Alex Zhavoronkov, Bhupinder Bhullar, Anton Buzdin

**Affiliations:** ^1^ Pathway Pharmaceuticals, Wan Chai, Hong Kong, Hong Kong SAR; ^2^ Group for Genomic Analysis of Cell Signaling Systems, Shemyakin-Ovchinnikov Institute of Bioorganic Chemistry, Moscow, Russia; ^3^ First Oncology Research and Advisory Center, Moscow, Russia; ^4^ Laboratory of Bioinformatics, D. Rogachyov Federal Research Center of Pediatric Hematology, Oncology and Immunology, Moscow, Russia; ^5^ National Research Centre “Kurchatov Institute”, Centre for Convergence of Nano-, Bio-, Information and Cognitive Sciences and Technologies, Moscow, Russia; ^6^ Pirogov Russian National Research Medical University, Department of Oncology, Hematology and Radiotherapy, Moscow, Russia; ^7^ Moscow 1st Oncological Hospital, Moscow Russia; ^8^ Engelhardt Institute of Molecular Biology, Russian Academy of Sciences, Mosow, Russia; ^9^ Moscow State University, Faculty of Fundamental Medicine, Moscow, Russia; ^10^ Insilico Medicine, Inc, ETC, Johns Hopkins University, Baltimore, MD, USA; ^11^ Novartis Institute for Biomedical Research, Basel, Switzerland

**Keywords:** transition from nevus to primary and metastatic melanoma, OncoFinder, intracellular molecular networks, metabolic and signaling pathways, machine learning algorithms

## Abstract

Melanoma is the most aggressive and dangerous type of skin cancer, but its molecular mechanisms remain largely unclear. For transcriptomic data of 478 primary and metastatic melanoma, nevi and normal skin samples, we performed high-throughput analysis of intracellular molecular networks including 592 signaling and metabolic pathways. We showed that at the molecular pathway level, the formation of nevi largely resembles transition from normal skin to primary melanoma. Using a combination of bioinformatic machine learning algorithms, we identified 44 characteristic signaling and metabolic pathways connected with the formation of nevi, development of primary melanoma, and its metastases. We created a model describing formation and progression of melanoma at the level of molecular pathway activation. We discovered six novel associations between activation of metabolic molecular pathways and progression of melanoma: for allopregnanolone biosynthesis, L-carnitine biosynthesis, zymosterol biosynthesis (inhibited in melanoma), fructose 2, 6-bisphosphate synthesis and dephosphorylation, resolvin D biosynthesis (activated in melanoma), D-myo-inositol hexakisphosphate biosynthesis (activated in primary, inhibited in metastatic melanoma). Finally, we discovered fourteen tightly coordinated functional clusters of molecular pathways. This study helps to decode molecular mechanisms underlying the development of melanoma.

## INTRODUCTION

Melanoma is a type of skin cancer formed from melanocytes, skin cells that produce the pigment melanin. Treatment of primary melanoma includes surgical removal, and in the case of early diagnosis, the US survival rate reaches 91%. However, melanomas are very active in forming metastases, and if not diagnosed at the early stage, the survival prognosis is poor [1]. Melanoma accounts for 75% of deaths related to skin cancer [1]. In 2012, melanoma occurred in 232,000 patients and resulted in 55,000 deaths worldwide [2]. Development of melanomas is commonly caused by mutations from UV linked DNA damage [3] and by inherited genetic factors like highly penetrant loss-of-function mutations in tumor suppressor genes *CDKN2A* and *XP* [4, 5]. About 40% of human melanomas contain activating mutations of the B-Raf protein, resulting in constitutive signaling through the Raf to MAP kinases growth signaling pathways [6]. The presence of multiple melanocytic nevi, a genetic trait compounded by sun exposure, also increases the risk of developing melanoma, although the transition from benign nevi to melanoma does not usually occur and what triggers this change is unknown.

Melanoma cells are characterized by a high mutation rate. Genome-wide sequencing of twenty-five human melanomas identified ∼100 structural rearrangements and ∼80,000 mutated bases per genome [7]. This is roughly 1100-times higher than the background mutation frequency in a normal human genome replicated between generations [8].

The molecular mechanisms of developing melanoma may be quite distinct. For example, UV irradiation causes keratinocytes to increase expression of multifunctional protein p53, which, by acting as a transcriptional factor, increases production of melanocyte-stimulating hormone (MSH) by these cells [9]. Secreted MSH molecules bind to melanocortin 1 receptors (MC1R) on the surface of melanocytes, which, in turn, promote the internal adenylate cyclase cascade and activate the CREB pathway, thus resulting in the activation of transcriptional factor MITF [10]. MITF, in turn, transactivates expression of p16 and Bcl2 proteins, which promote survival of melanocytes [11].

Alternatively, B-Raf, and its downstream signaling pathway through MAP kinases, directly promotes cell proliferation leading in melanomas, as evidenced by the positive clinical trials for B-Raf inhibitor drugs Dabrafenib and Vemurafenib [13, 14].

Another feature of invasive and metastatic melanoma cells is their ability to suppress the immune system, e.g. by overproducing CTLA-4 protein receptor, which inactivates T-cells [12]. Targeting this protein by the recently developed anticancer drug Ipilimumab showed enhanced survival for the advanced melanoma patients [13].

To learn more about the mechanisms that induce melanoma and cause it to progress, we performed high-throughput analysis of melanoma-related intracellular molecular networks including 592 signaling and metabolic pathways. We profiled a total of 478 transcriptomes corresponding to primary and metastatic melanoma, nevi and normal tissue samples. Using a combination of statistics and machine learning algorithms, we found characteristic sets of signaling and metabolic pathways activated or repressed during the development of primary melanoma from normal skin and also during its further progression to the metastatic state. We provide evidence that, at the molecular pathway level, formation of nevi clearly resembles the transitional state from normal skin to primary melanoma. For each stage of skin-to-melanoma transition, we identified characteristic molecular pathways, many of which are novel associations. Using bioinformatics analysis combined with various statistics and machine learning algorithms, we then created a stable model describing formation and progression of melanoma at the level of molecular pathway activation. Understanding the molecular mechanisms of melanoma development will be key in developing new treatment strategies.

## RESULTS AND DISCUSSION

### Bioinformatics tool for the analysis of intracellular signaling and metabolic pathways

We processed transcriptomic data from primary and metastatic melanoma, nevi, and reference normal samples to establish pathway activation strength (PAS) profiles corresponding to signaling and metabolic intracellular molecular pathways. Several approaches were published previously by us and others to measure PAS based on large scale gene expression data, either transcriptomic or proteomic. Khatri *et al.* [14] classified those methods into three major groups: Over-Representation Analysis (ORA), Functional Class Scoring (FCS) and Pathway Topology (PT)-based approaches. ORA-based methods calculate whether the pathway is significantly enriched with differentially expressed genes [15]. These methods have many limitations, as they ignore all non-differentially expressed genes and do not account for many gene-specific characteristics. FCS-based approaches partially tackle aforementioned limitations by calculating fold change-based scores for each gene and then combining them into a single pathway enrichment score [16]. PT-based analysis also takes into account topological characteristics of each given pathway, assigning additional weights to the genes (for a review, see [17]). To account for gene expression variability within a pathway, a set of differential variability methods has been developed [18]. Differential variability analysis determines a group of genes with a significant change in variance of gene expression between case and control groups [19]. This approach was further extended and applied on the pathway level [20].

Recently, we developed a new biomathematical method for pathway analysis, termed OncoFinder [21]. Based on kinetic models that use the “low-level” approach of mass action law, OncoFinder performs quantitative and qualitative enrichment analysis of the signaling pathways. For each sample investigated, it performs a case-control pairwise comparison and calculates the Pathway Activation Strength (PAS), a value which serves as a qualitative measure of pathway activation. Unlike most other methods, this approach determines if the signaling pathway is significantly up- or down-regulated compared to the reference. Negative and positive overall PAS values correspond to an inhibited or activated state of signaling pathway [21].

OncoFinder is also, to our knowledge, a unique PAS calculating method, which was reported to provide output data with significantly reduced noise introduced by the experimental transcriptome profiling systems [22]. This method was shown to be efficient in finding new cancer biomarkers, more stable than individual gene expression patterns [23]. Since its development, Oncofinder has been applied to the analysis of transcriptomes of various conditions, including leukemia and solid cancers [24–26], Hutchinson Gilford Disease [27] and Age-Related Macular Degeneration Disease [28].

Here, we updated the OncoFinder algorithm and the corresponding databases, which were originally developed to analyze only intracellular signaling pathways, to a new version supporting the analysis of both signaling and metabolic pathways. To build the internal interactions database for metabolic pathways, we used the publicly available HumanCyc database (www.humancyc.org). The resulting database used in this study contained 271 signaling and 321 metabolic intracellular pathways (Supplementary Dataset S1).

### Intracellular signaling and metabolic pathway activation profiles

In this study, we profiled a group of 478 human transcriptomes consisting of 132 human primary melanoma, 222 metastatic melanoma, 103 normal skin and 21 nevi samples (Table 1).

**Table 1:** Summary of transcriptomic datasets used in this study

Dataset ID	Experimental platform	Skin samples	Nevus	Primary melanoma	Metastatic melanoma
GSE 7553	GPL570	5	0	14	40
GSE 53223	GPL570	6	12	0	0
GSE 46517	GPL96	8	9	31	52
GSE 39612	GPL570	64	0	0	0
GSE 31879	GPL570	4	0	10	0
GSE 23376	GPL570	0	0	0	22
GSE 19234	GPL570	0	0	0	44
GSE 15605	GPL570	16	0	46	12
GSE 8401	GPL96	0	0	31	52

**Figure 1:**
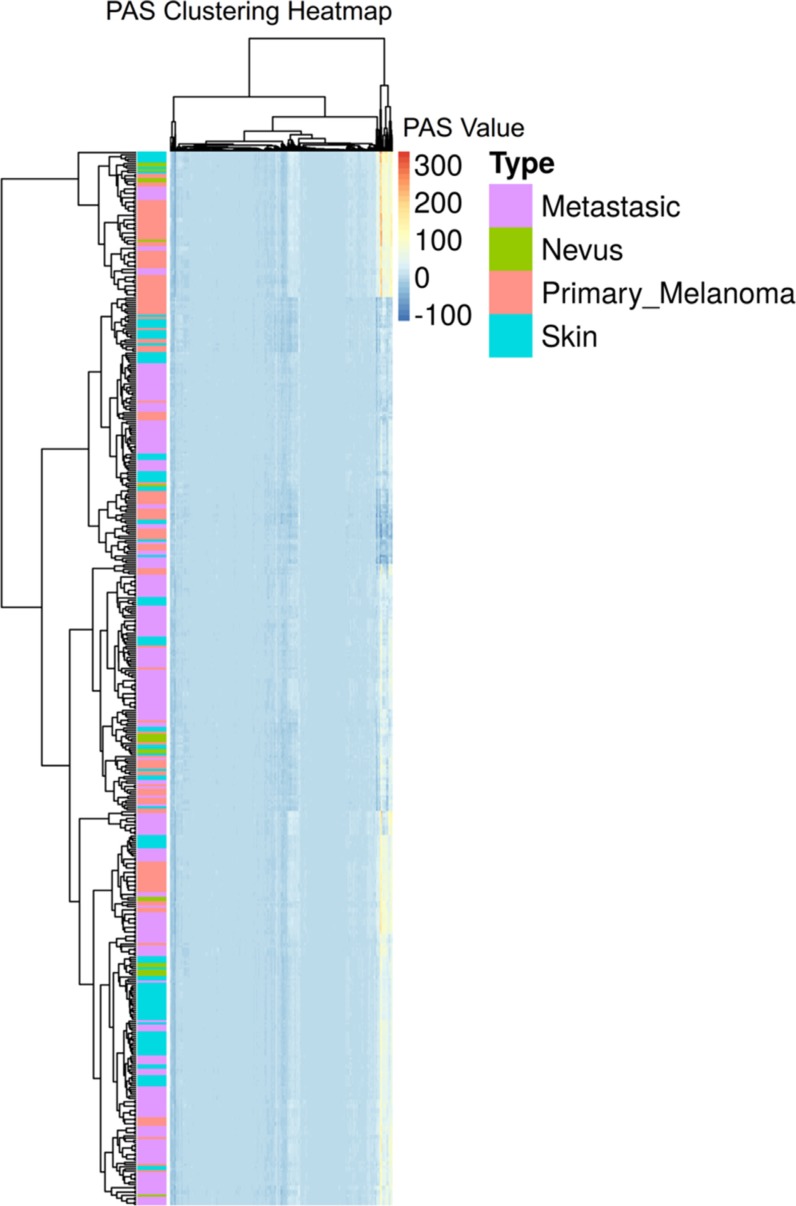
Hierarchical clustering heatmap of all samples and all molecular pathways under investigation

The normalized gene expression data were next processed using the OncoFinder algorithm to establish pathway activation strength (PAS) profiles. The complete PAS data are shown on Supplementary Dataset S2. To assess the functional relations between the investigated groups of samples, we built hierarchical clustering heatmaps with Ward method using Euclidean distance for all samples and all investigated molecular pathways (Figure 1). We observed rather uncertain clustering features hardly distinguishing between the four sample classes. To increase the resolution of clustering methods and to identify features that distinguish the above functional groups, we applied a selection of machine learning classifier algorithms.

### Enhanced sampling classification with machine learning algorithms

We used several different machine classifiers, including Random Forest (RF) Support Vector Machines (SVM) with Linear and Radial kernels, Partial Least Squares (PLS) and Generalized linear regression with Glmnet regularization. Prior to classification, we filtered for small deviation and collinearity to prevent using two highly correlated variables when one would suffice. Such approaches allowed us to achieve ∼0.94 average balanced accuracy of a 4-class problem (classification into four groups: Skin, Nevi, Primary and Metastatic melanoma) using only metabolic pathways (Table 2) and ∼0.94 average balanced accuracy using only signaling pathways (Table 3). In accordance with their vague transitional state, the most difficult group for all the classifiers used were nevi, for which the classifiers showed lowest combinations of sensitivity (0.4–0.8) and balanced accuracy (0.7–0.9) (Tables 2–3). Full statistical comparison of different classifiers are shown on Supplementary Dataset S3. Groups other than nevi formed significantly more clear-cut clusters, which corresponded to their physiologically distinct states. Overall, the SVM family classifiers showed the best results compared to other models.

**Table 2:** SVM with Radial kernel classification report based on metabolic pathways

SVM Radial	Sensitivity	Specificity	Balanced accuracy
Metastatic Melanoma	0.927	0.952	0.94
Nevus	0.8	0.991	0.896
Primary Melanoma	0.909	0.965	0.937
Skin	1	0.989	0.995

Average balanced accuracy 0, 941.

**Table 3:** SVM with Radial kernel classification report based on signaling pathways

SVM Radial	Sensitivity	Specificity	Balanced accuracy
Metastatic Melanoma	0.909	0.937	0.923
Nevus	1	0.973	0.987
Primary Melanoma	0.879	0.941	0.91
Skin	0.88	1	0.94

Average balanced accuracy 0, 939.

### Identification of top pathways discriminating transition from skin to nevi and melanoma

**Figure 2:**
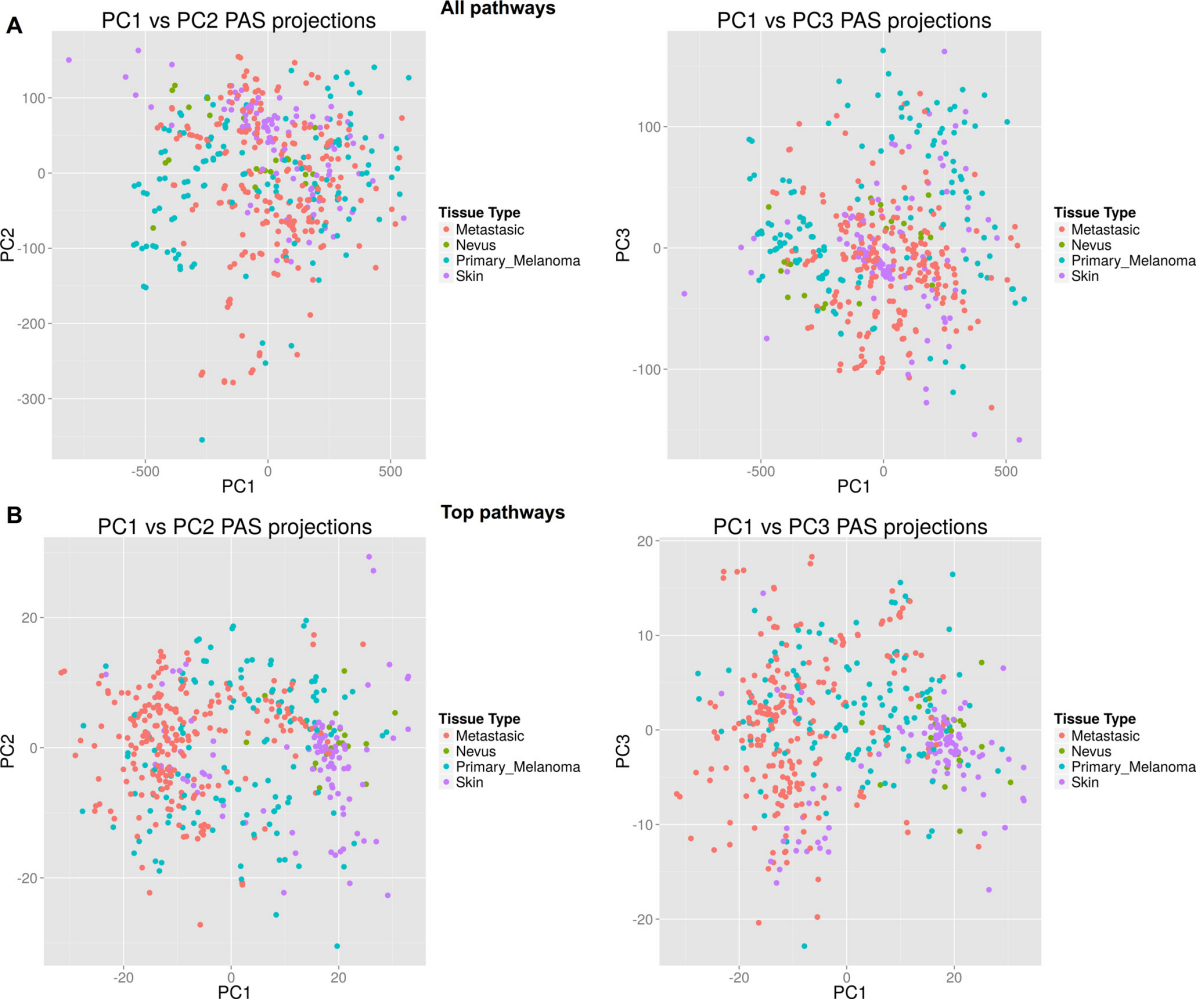
Scatterplots for principal component analysis (**A**) Results built for all metabolic and signaling pathways. (**B**) Results built for top characteristic metabolic and signaling pathways.

To further analyze progression of melanoma and relevant molecular events at the level of pathway activation, we used information of variable importance during the process of classification. For each statistical model, we identified the top 30 metabolic and top 30 signaling pathways, distinguishing the two classes using the “varImp” function from “caret” package, which unifies different techniques of measuring importance between different models (Supplementary Dataset S3). Next, the top pathways were intersected and a list of consensus pathways was established (Tables 4–5). The consensus records included 25 metabolic and 19 signaling pathways for two different models of melanoma development, the first occurring via transitional state of the nevus (Skin → Nevus → Melanoma) and the second not involving nevus (Skin → Primary Melanoma → Metastatic Melanoma). To test the classification power of these top pathways, we built a new hierarchical clustering heatmap with the Ward method, using Euclidean distance for all samples and top investigated molecular pathways with supporting Principal Components Analysis (PCA) projections plots (Figure 2). These top pathways enabled significantly better discrimination between the groups, as evidenced by PCA projections plots for all pathways (Figure 2A) compared to plots for the selected top pathways (Figure 2B). Next, we used these top pathways in the same 4-type prediction model as before. Results for best model (SVM Linear model) confirmed adequacy of the classifier pathway selection and showed an averaged balanced accuracy of ∼0.93, very close to the model with full pathways (Table 6, Supplementary Dataset S4).

**Table 4:** Top metabolic pathways implicated in progression of melanoma

Pathway	Nevus vs Skin	Pr. Mel vs Skin	Met. Mel vs Skin	Met. Mel vs Pr. Mel	Primary vs Nevus
allopregnanolone biosynthesis	UP	DOWN	DOWN	DOWN	DOWN
citrulline-nitric oxide cycle	UP	DOWN	DOWN	DOWN	DOWN
dTMP ide novoi biosynthesis mitochondrial	DOWN	UP	UP	UP	UP
L-carnitine biosynthesis	UP	DOWN	DOWN	DOWN	DOWN
5-aminoimidazole ribonucleotide biosynthesis	DOWN	UP	UP	UP	UP
eumelanin biosynthesis	UP	UP	UP	DOWN	DOWN
putrescine biosynthesis II	DOWN	DOWN	UP	UP	UP
pyrimidine deoxyribonucleosides salvage	DOWN	UP	UP	UP	UP
spermine and spermidine degradation I	UP	DOWN	DOWN	DOWN	DOWN
superpathway of tryptophan utilization	UP	DOWN	DOWN	UP	DOWN
tryptophan degradation X mammalian via tryptamine	UP	DOWN	DOWN	DOWN	DOWN
1D-imyoi-inositol hexakisphosphate biosynthesis V from Ins134P3	UP	UP	DOWN	DOWN	UP
D-mannose degradation	UP	UP	UP	UP	DOWN
fructose 26-bisphosphate synthesis, dephosphorylation	UP	UP	UP	DOWN	DOWN
histamine biosynthesis	UP	DOWN	DOWN	DOWN	DOWN
inosine-5-phosphate biosynthesis	UP	UP	UP	UP	DOWN
melatonin degradation II	UP	DOWN	DOWN	DOWN	DOWN
pyrimidine deoxyribonucleosides degradation	UP	UP	UP	DOWN	UP
resolvin D biosynthesis	UP	UP	UP	DOWN	UP
retinoate biosynthesis I	DOWN	DOWN	DOWN	UP	UP
superpathway of steroid hormone biosynthesis	UP	DOWN	DOWN	DOWN	DOWN
tRNA charging	UP	UP	UP	UP	UP
UDP-N-acetyl-D-galactosamine biosynthesis II	UP	UP	UP	UP	DOWN
valine degradation	DOWN	DOWN	DOWN	UP	DOWN
zymosterol biosynthesis	UP	DOWN	DOWN	DOWN	DOWN

UP or DOWN indicates positive and negative difference between the state of interest (nevus, primary and metastatic melanoma) and skin in median PAS value, respectively.

**Table 5:** Top signaling pathways implicated in progression of melanoma

Pathway	Nevus vs Skin	Pr. Mel vs Skin	Met. Mel vs Skin	Met. Mel vs Pr. Mel	Pr. Mel vs Nevus
Fas Signaling Pathway (Negative)	DOWN	UP	UP	UP	UP
cAMP Pathway (Glycolysis)	UP	DOWN	DOWN	UP	DOWN
CD40 Pathway (Cell Survival)	UP	UP	UP	UP	UP
AKT Pathway (Protein Synthesis)	UP	DOWN	DOWN	DOWN	DOWN
ATM Pathway (Apoptosis, Senescense)	DOWN	UP	UP	UP	UP
BRCA1 Main Pathway	UP	UP	UP	UP	UP
cAMP Pathway (Endothelial Cell Regulation)	UP	DOWN	DOWN	DOWN	DOWN
cAMP Pathway (Myocardial Contraction)	DOWN	DOWN	DOWN	DOWN	DOWN
cAMP Pathway (Protein Retention)	DOWN	UP	UP	UP	UP
Caspase Cascade (Apoptosis)	UP	DOWN	DOWN	DOWN	DOWN
CD40 Pathway (IKBs Degradation)	UP	UP	UP	UP	UP
DDR pathway Apoptosis	DOWN	UP	UP	UP	UP
Glucocorticoid Receptor Pathway (Cell cycle arrest)	UP	DOWN	DOWN	DOWN	DOWN
HGF Pathway (PKC pathway)	UP	UP	UP	UP	DOWN
HIF1-Alpha Main Pathway	UP	UP	UP	UP	UP
JNK Pathway (Insulin signaling)	UP	DOWN	DOWN	DOWN	DOWN
mTOR Pathway (VEGF pathway)	DOWN	DOWN	UP	UP	DOWN
PAK Pathway (Myosin Activation)	DOWN	DOWN	DOWN	DOWN	DOWN
Ubiquitin Proteasome Pathway (Degraded Protein)	DOWN	UP	UP	UP	UP

UP or DOWN indicates positive and negative difference between the states of interest (nevus, primary and metastatic melanoma) and skin in median PAS value, respectively.

**Table 6:** SVM with Linear kernel method classification report based on combination of top signaling and metabolic pathways

SVM Linear	Sensitivity	Specificity	Balanced accuracy
Metastasic	0.909	0.889	0.899
Nevus	1	0.991	0.996
Primary Melanoma	0.788	0.965	0.876
Skin	0.96	0.978	0.969

Average balanced accuracy 0, 935.

On the heatmap and PCA projection plots, the samples corresponding to nevi formed a cloudy group and clustered either with each other or diffusely between primary melanoma and normal skin samples. In agreement with previous reports, this suggests that nevi form a complicated group of highly variable samples, which frequently correspond to the intermediate state between normal skin and primary melanoma [29]. The top classifier elements included 25 metabolic and 19 signaling pathways. For all of these *signaling* pathways, association with melanoma was reported previously in the literature. However, for the *metabolic* pathways, this was not the case, and previous reports on the association with melanoma were not found for the following (Table 4): Allopregnanolone biosynthesis, L-carnitine biosynthesis, Zymosterol biosynthesis (inhibited in melanoma), D-myo-inositol hexakisphosphate biosynthesis (activated in primary, inhibited in metastatic melanoma), Fructose 2, 6-bisphosphate synthesis and dephosphorylation, Resolvin D biosynthesis (activated in melanoma). Thus, we report here six novel associations between activation of metabolic molecular pathways and progression of melanoma.

### Functional significance of characteristic molecular pathways

We observed a number of molecular pathway features distinguishing the transition from normal skin to either nevi or primary and metastatic melanomas (Tables 4, 5).

In melanomas, we identified several characteristically activated tumor suppressor pathways, like Fas signaling, a branch of ATM signaling leading to apoptosis and senescence, and the DDR pathway, leading to apoptosis. However, caspase cascade members were strongly inhibited in melanomas, which suggests that cancer cells efficiently escape cell death initiated at the upstream stages. This may be achieved by degradation of tumor suppressor proteins using proteasome-linked mechanisms (activated in both types of melanoma), and by activating cell survival and proliferation pathways like branches of CD40 signaling, HGF signaling and HIF1-Alpha pathway (activated in melanomas). Interestingly, the BRCA1 pathway dealing with DNA repair was strongly stimulated in melanomas, which may contribute to the relative inefficiency of radiation therapy treatment of metastatic melanomas.

Actively replicating cancer cells are faced with a lack of oxygen (hypoxia) and thus have to switch their energetic balance from oxidative phosphorylation to glycolysis. Glycolysis, which is based on anaerobic conversion of glucose to lactate, becomes the major source of ATP and NADH in the cells. This phenomenon, known as Warburg effect, is characteristic for most cancer cells [30]. Glycolysis is also the major source of substrates for basic biosynthetic pathways, e.g. those dealing with building ribonucleotides and amino acids [31]. For both nevi and melanomas, we observed increased expression of mannose-6-phosphate isomerase, an enzyme that makes it possible to utilize the sugar mannose as substrate for glycolysis. This, in turn, may intensify production of ATP by the transformed cells.

On the other hand, nevi-independent transformation to melanomas was characterized by gradual decrease of the nitric oxide (NO) biosynthesis pathway. NO is a known mediator of cancer aggressiveness. Mechanisms of its influence depend greatly on concentration and time of exposure. Low concentrations stimulate proliferation of cancer cells, whereas high levels of NO may cause antitumor effects [32]. Low levels (< 300 nM) promote cell survival and proliferation by activating mTOR, cyclic GMP signaling, by Akt phosphorylation and by stabilization of hypoxia-induced factor HIF-1α [33]. At the same time, low NO activates glycolysis by stimulating AMP-protein kinases, by increasing concentrations of fructose-2, 6-bisphosphatase, and by promoting glucose uptake by the cells [34]. In contrast, increased levels of NO (greater than 300 nM) promote activation of p53 signaling and related cytostatic and apoptotic effects linked with inactivation of ERK and Akt signaling [35].

We speculate that the apparently stimulated NO synthesis pathway in nevi may be one of the factors that stabilizes their condition and prevents cancer transformation. Activity of the enzymes involved in NO biosynthesis, like arginine succinate synthase (ASS) depends on the regulation by glucocorticoid hormones cAMP, glucagon and insulin. Reduced expression of ASS was previously reported in the literature for melanoma and other tumors [36]. Cancer cells frequently are unable to synthesize sufficient amounts of arginine and thus fully depend on the import of this amino acid from blood. It was shown previously that arginine deprivation in blood using the enzyme arginine deimynidase may result in cancer regression [36]. Moreover, arginine is tightly associated with the metabolism of polyamines in eukaryotic cells. Arginine is the precursor of ornithine, which is a substrate for further biosynthesis of polyamines [37]. Thus, reduction of arginine biosynthesis may cause altered concentrations and metabolism for putrescin, spermidine, spermine and other polyamines in melanoma. In this study, we show suppressed degradation of spermine and spermidine in both types of melanoma, which may help the cells to store the synthesized polyamines. The polyamines are major organic cations presenting in the eukaryotic cells. These molecules are absolutely necessary for cell growth and differentiation [38]. They can non-specifically activate DNA replication, transcription and translation [39]. For example, increased concentrations of polyamines stimulate proliferation, increase synthesis of the external matrix proteins and enhance angiogenesis [40]. Ornitindecarboxylase (ODC) is the first enzyme in the pathway of polyamine synthesis. In transgenic mice, progression of melanoma depends on the biosynthesis of polyamines, particularly putrescin, as treatment with ODC inhibitors inhibits tumor growth and causes its rapid regression [41]. Moreover, inhibitors of polyamine synthesis enzymes efficiently decrease frequencies of spontaneous skin cancers caused by UV irradiation or induced by chemicals in different experimental models. In contrast, artificial induction of ODC activity causes a sequential increase in concentrations of polyamines followed by enhanced frequencies of skin cancers [41].

Furthermore, we observed sequential inhibition of a pathway of histamine biosynthesis in both types of melanoma. Histamine is a diamine molecule that has important neurotropic activities but also modulates immune response by increasing permeability of blood vessels to leukocytes and proteins [42]. In line with our observations, an antagonistic relationship was previously reported for polyamines and histamine because of partial inhibition of intracellular uptake of polyamines by the histamine [42].

Another feature distinguishing normal skin, nevi, and melanomas was the pathway of carnitine biosynthesis, apparently activated in nevi and inhibited in melanomas. Carnitine is a compound required for fatty acid metabolism in mammals. When entering the cell, free fatty acids are oxidized to form acil-CoA molecules, which are next transferred to the mitochondrial matrix using carnitine-palmitoyl transferase 1 (CPT1) and carnitine [43]. The CPT1-based transfer system is, therefore, directly linked with the biosynthesis of carnitine and a hypoxic state [44]. Carnitine is synthesized using two amino acid substrates, lysine and methionine, in a reaction catalyzed by trimethyl-lysine-dioxygenase (TMLH) [45]. The observed increase in carnitine biosynthesis regulation may be connected with the hypoxic conditions characteristic for primary and metastatic melanomas, both of which feature upregulation of the hypoxia-induced factor 1 (HIF1) pathway. The melanoma cells, therefore, may be deficient in their ability to use fatty acids as the substrate for oxidative phosphorylation; this deficiency may be an object of future molecular therapeutic approaches.

We also noticed decreased activation of the tryptophan degradation pathway in melanomas, which may lead to tryptophan accumulation in the cells. Tryptophan is the precursor of many signaling molecules, including melatonin and serotonin [46], and also of NAD and niacin. Congruently, we found that a pathway of melatonin degradation is decreased in both types of melanoma. Thus, regulation of melatonin and tryptophan degradation may be correlated in melanoma cells.

### Generalized molecular model of transition from normal skin to melanoma and nevi

**Figure 3:**
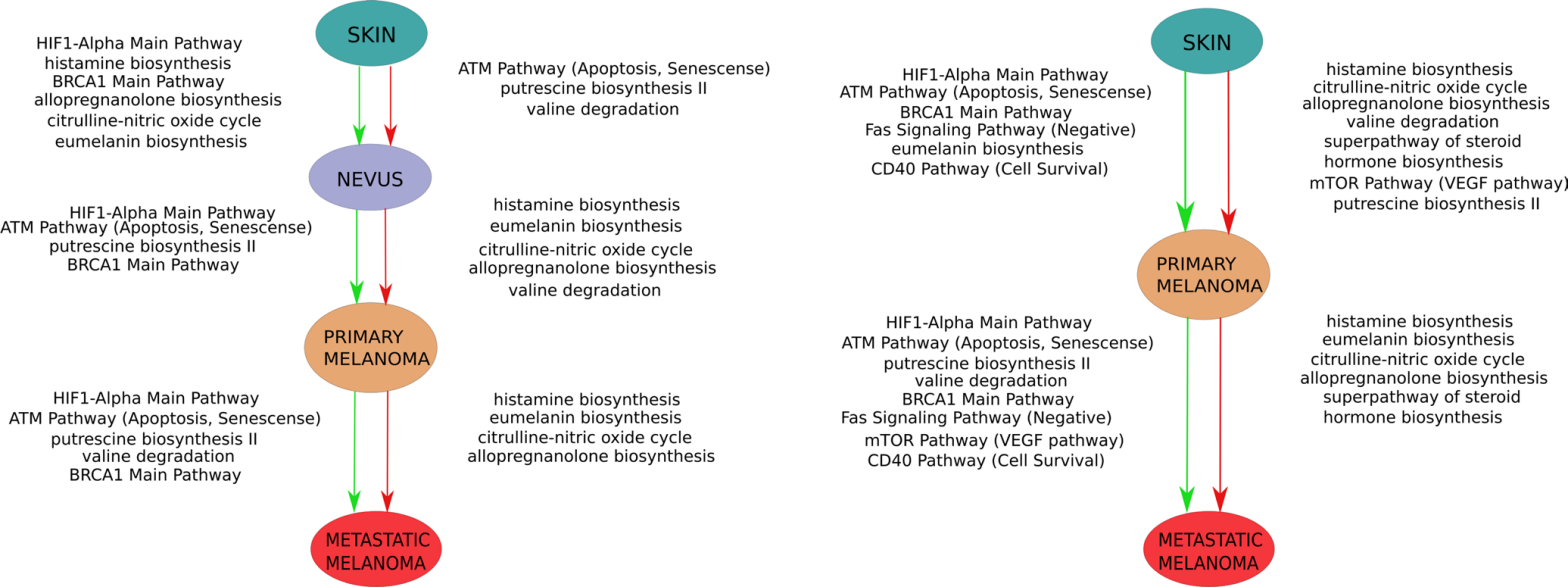
Schematic representation of two alternative models of melanoma progression built in this study One model comprises transition from skin to primary melanoma versus “nevus” stage (left panel), the second – direct transition from skin to primary melanoma (right panel). Green arrows indicate activated molecular pathways, red arrows – suppressed pathways.

We found 25 metabolic and 19 signaling pathways that were good-quality characteristic discriminators between the classes of normal skin, nevus, primary melanoma and serotonin metastatic melanoma (Tables 4, 5). We considered two general models of melanoma formation and transformation including transitions (i) Skin → Nevus → Prtimary melanoma→ Metastatic melanoma) and nevus-independent model (ii) Skin → Primary Melanoma → Metastatic Melanoma (Figure 3). In both transition axes, HIF1-alpha and BRCA1 pathways were gradually increasing when moving from normal state to metastatic melanoma.

Transition from *normal skin to nevi* compared to primary melanoma was very peculiar because it included activation of histamine, allopregnanolone, and citrulline – NO cycle biosynthesis pathways. Eumelanin biosynthesis and BRCA1, HIF1-alpha signaling pathways were also activated. Several pathways were also suppressed in nevi, in contrast to primary and metastatic melanomas; these included putrescin biosynthesis, valine degradation, and the senescence/apoptotic branch of the ATM pathway.

Transition from *normal skin to primary melanoma* was characterized by upregulation of the eumelanin biosynthesis pathway, BRCA1, HIF1-alpha pathways, senescence/apoptotic branch of the ATM pathway, cell death-promoting Fas signaling pathways, and the cell survival-promoting branch of the CD40 pathway. In turn, pathways of putrescine, histamine, allopregnanolone, steroid hormone and citrulline – NO cycle biosynthesis and of valine degradation were inhibited in primary melanoma compared to skin.

Transition from *nevus to primary melanoma* showed upregulation of the BRCA1, HIF1-alpha pathways, senescence/apoptotic branch of the ATM pathway, and putrescine biosynthesis pathway. Inhibited pathways were histamine, allopregnanolone, eumelanin biosynthesis and citrulline – NO cycle biosynthesis and of valine degradation.

Finally, transition from *primary to metastatic melanoma* comprised upregulation of BRCA1, HIF1-alpha pathways, the senescence/apoptotic branch of the ATM pathway, putrescine biosynthesis, and valine degradation pathways. Inhibited pathways were histamine, allopregnanolone, eumelanin biosynthesis, and citrulline – NO cycle biosynthesis. The complete list of characteristic pathways is shown on Tables 4 and 5.

### Epigenetic regulation of melanoma advancement

The HIF1 pathway is specifically activated in the critical transitions from nevus to primary melanoma and, again to the more aggressive metastatic melanoma. HIF1-alpha has a tight correlation to epigenetic mechanisms. It has been demonstrated that HIF1-alpha induces expression of histone demethylases, JARID1C and JMJD1A for example, in colorectal cancer cells, which directly promote malignant development by epigenetic mechanisms [47, 48]. Since chromatin remodeling is associated with changes in gene expression, a closer look at the pathways regulated by HIF1-alpha, would help elucidate significant molecular pathways promoting the transitions to primary and metastatic melanoma, This does not preclude other epigenetic mechanisms that may be specific to the development of melanoma. For example, studies have shown the role of micro-RNA, miR-211 and miR-375, have tumor suppressive functions in melanocytes [49, 50]. Silencing of both micro-RNA species have been observed in melanoma, and indeed, epigenetic downregulation of miR-375 locus was observed as one mechanism of this silencing [50]. Taken together, the role of epigenetics in melanoma needs to be investigated further, and using Pathway Activation Scoring, we may be able to delineate the relationships with the metabolic and signaling pathways that we have described above.

### Congruent activation of various sets of molecular pathways

For the first time, we explored here the activity of 592 signaling and metabolic pathways using the hierarchical clustering assay. We applied the Weighted Correlation Network Analysis (WGCNA) method to identify similar regulation patterns between the molecular pathways. We found that molecular pathways form 14 distinguishable clusters, each characterized by concordant activation signatures of the enclosing pathways (Jaccard structural similarity index signatures are shown separately for each cluster on Supplementary Dataset S5). All pathways in clusters were filtered according to their paired and overall correlation coefficients. All molecular pathways from each cluster are listed in Supplementary Dataset S6.

**Figure 4:**
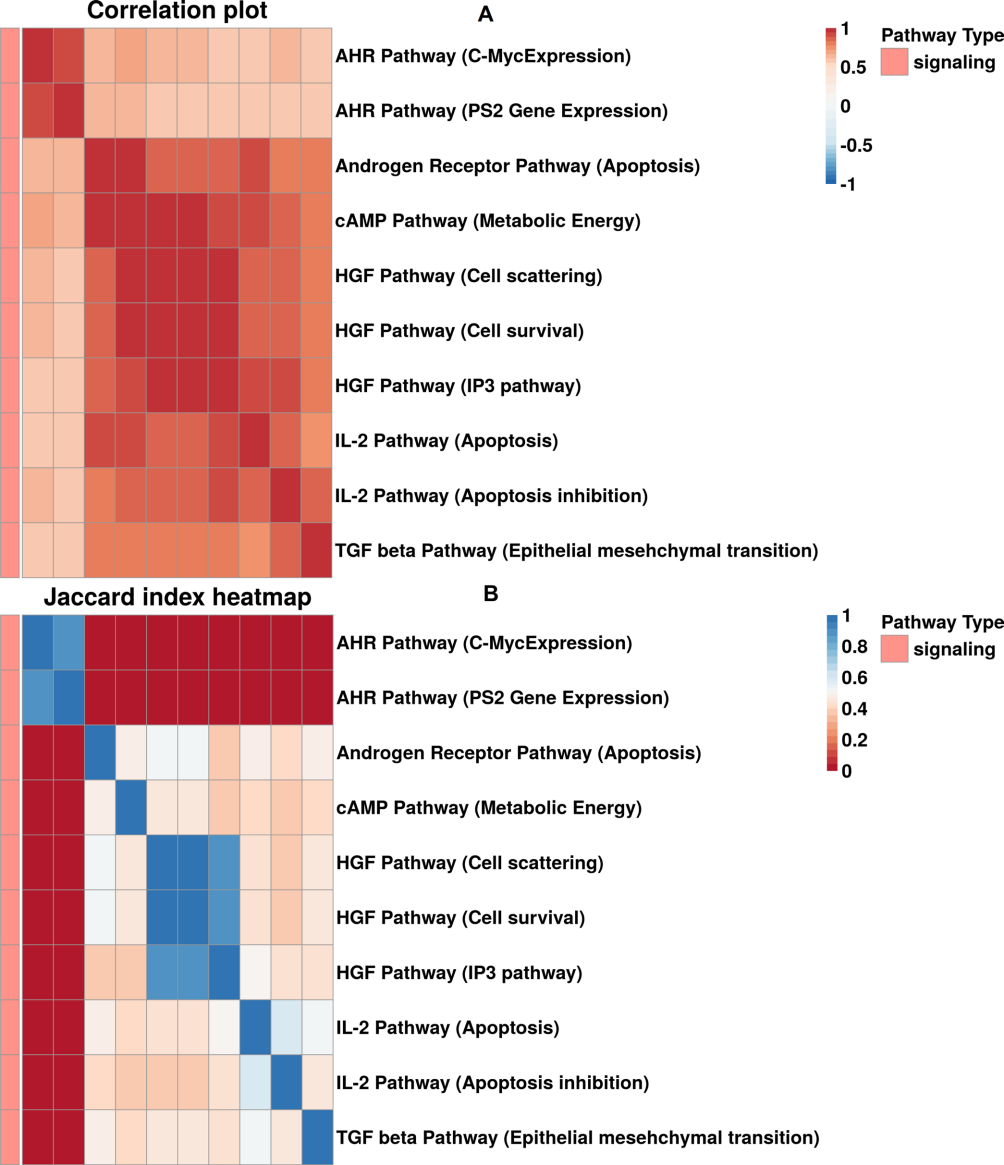
(**A**) Correlation plot built for cluster 6. (**B**) Heatmap of Jaccard gene intersection index between pathways in cluster 6.

In some instances, congruent activation for the pathways forming the same clusters can be explained by the structural similarities between the cluster-forming pathways – e.g., AKT Pathway (Caspase Cascade) and AKT Pathway (p73 Mediated Apoptosis) from cluster #8, or p53 Signaling (Negative) Pathway (p53 Degradation) and Wnt Pathway (Ctnn-b Degradation) from cluster #5 are highly similar in their gene product composition (Supplementary Dataset S5). However, for the majority (10 out of 14) of clusters, pathways were combined not due to similar gene content, but rather because of the true functional coordination between the cluster members (Supplementary Dataset S5). For example, for cluster 6 shown on Figure 4, most of the enclosing pathways have low structural similarity, but are at the same time strongly functionally coordinated, as reflected by low Jaccard indexes and high PAS correlation scores, respectively. This common regulation of various molecular pathways is a novel finding and will be analyzed in detail in further studies.

## CONCLUSIONS

Here, we provide evidence that at the molecular pathway level, nevi largely correspond to a transitional state from normal skin to primary melanoma. We found 44 signaling and metabolic pathways connected with the formation of nevi and with the development of primary melanoma and its metastases. We created a stable model describing formation and progression of melanoma at the level of molecular pathway activation. Many of these pathways had never been previously associated with melanoma. We found six novel associations between activation of metabolic molecular pathways and progression of melanoma. Finally, we discovered fourteen tightly coordinated functional clusters of molecular signaling and metabolic pathways. This study helps to decode molecular mechanisms underlying development of melanoma.

## MATERIALS AND METHODS

### Transcriptomic datasets

We obtained from NCBI GEO repository (www.ncbi.nlm.nih.gov/geo/) nine datasets containing transcriptomic information for human tissues related to melanoma: GSE7553, GSE53223, GSE46517, GSE39612, GSE31879, GSE23376, GSE19234, GSE15605 and GSE8401, and one dataset with the transcriptomic data for Agilent Universal Human RNA Reference (GSE3061). For all the datasets, experimental transcriptional profiling was performed using Affymetrix GPL90 and GPL570 platforms. The data from different datasets were quantile-normalized using «affy» package for R 3.1.1 for each platform, and afterwards combined together followed by the harmonization with the XPN algorithm for cross-platform normalization of samples [51]. Each sample under study, except Agilent Universal Human RNA Reference, was classified to one of the groups below: “Normal Skin”, “Primary Melanoma”, “Metastatic Melanoma” or “Nevi”, according to dataset description. In total, we included 103 Skin, 21 Nevus, 132 Primary Melanoma, and 222 Metastatic Melanoma samples in our study.

### Functional annotation of gene expression data

For the functional annotation of gene expression data at the molecular pathway level, we applied OncoFinder algorithm, recently published by Buzdin *et al.* [21]. It operates with calculation of the Pathway Activation Strength (PAS), a value which serves as a qualitative measure of a molecular pathway activation. The formula for PAS calculation accounts for gene expression data and for information on the protein interactions in a pathway, namely, individual protein activator or repressor roles in a pathway [24].

The positive value of PAS indicates abnormal activation of a signaling pathway, and the negative value - its repression. Here, the case-to-normal ratio, CNR_n_, is the ratio of expression levels for a gene *n* in the sample under investigation to the same average value for the control group of samples. In addition, for each CNR value, we applied multiplication to a Boolean flag of BTIF (beyond tolerance interval flag), which equals to 1 when the CNR value passed, and to 0 when CNR value did not pass both or either one of the two criteria of significantly differential expression: first, the expression level for the sample must fit outside of the tolerance interval for norms, with *p* < 0.05, and second, the value of CNR must differ from 1 by at least 1.5-fold. For each sample, we obtained results for the 269 signaling and 363 metabolic pathways, in standard application using Agilent Universal Human RNA Reference samples as the norms. Alternatively, for some calculations, the norms were the normal human skin samples.

### Classification and feature selection

We applied the following frequently used machine learning algorithms for classification of the samples – SVM (Support Vector Machine) with linear and radial kernel (“e1071” package), Random Forest (“rf”), Lasso (“glmnet”), Partial Least Squares (“pls”) and Boosted Logistical Regression (“caTools”), Naive Bayes Classifier (“klaR”). R package “caret” was employed to implement uniform interface for each of the above learning algorithms. To prevent overfitting, we used repeated 10-fold cross-validation. Before training these classifiers, we performed different feature selections – features (PAS scores for different molecular pathways) with near zero variance were removed using the helper function from “caret” package termed “nearZeroVar”, while collinear predictors were identified and removed with the function “findCorrelation” from “caret” package, with threshold setting 0.85.

### Identification of top molecular pathways

We applied a two-step scheme to identify the most relevant pathways for classification of the melanoma samples. At the first step, we used function “varImp” from the caret package to obtain the top 30 pathways for each classificator model. Next, we looked for the intersections among all the models. The second step was the Kruskall-Wallis test, implemented in package “agricolae,” and removal of pathways not significantly differentially regulated (*p* > 0.05) between at least two different classes in progressions Skin→ Nevus → Primary Melanoma, and Skin → Primary Melanoma → Metastasic Melanoma.

### Clustering

To explore subtypes of primary and metastasic melanoma, we applied the hierarchical clustering method “hclust” with Ward distance and cutted clustering dendrogram at height 2000. We used R package “WGCNA” to find clusters of correlating pathways in melanoma samples. Function “blockwiseModules” was used to create a network, with minimum of 5 pathways in subnetworks. A correlation diagram was built using function “pheatmap” from the package “pheatmap,” sorted with respect to hierarchical clustering.

Each pathway in a cluster was filtered by correlation with other paths in the cluster – either with a mean modulus of correlation coefficient more than 0.7, or with at least one coefficient more than 0.85 and not 1 (correlation with itself). For each cluster, we also built heatmaps of Jaccard coefficient of gene intersections.

### Heatmaps and statistical analysis

We built our hierarchical clustering heatmaps using function “pheatmap” from “pheatmap” package; we also used this to create cluster images and correlational plots. PCA plots were made with help of “prcomp” function in R. Changes in pathway activation in different groups were measured by median change and *t*-test with multiple test correction.

## SUPPLEMENTARY MATERIALS DATASETS















## References

[1] Jerant AF, Johnson JT, Sheridan CD, Caffrey TJ (2000). Early detection and treatment of skin cancer. Am Fam Physician.

[2] Stewart BW, Wild C, International Agency for Research on Cancer, World Health Organization (2014). World cancer report 2014.

[3] El Ghissassi F, Baan R, Straif K, Grosse Y, Secretan B, Bouvard V, Benbrahim-Tallaa L, Guha N, Freeman C, Galichet L, Cogliano V (2009). WHO International Agency for Research on Cancer Monograph Working Group. A review of human carcinogens—part D: radiation. Lancet Oncol.

[4] Greene MH (1999). The genetics of hereditary melanoma and nevi. 1998 update. Cancer.

[5] Halachmi S, Gilchrest BA (2001). Update on genetic events in the pathogenesis of melanoma. Curr Opin Oncol.

[6] Davies MA, Samuels Y (2010). Analysis of the genome to personalize therapy for melanoma. Oncogene.

[7] Berger MF, Hodis E, Heffernan TP, Deribe YL, Lawrence MS, Protopopov A, Ivanova E, Watson IR, Nickerson E, Ghosh P, Zhang H, Zeid R, Ren X (2012). Melanoma genome sequencing reveals frequent PREX2 mutations. Nature.

[8] Campbell CD, Chong JX, Malig M, Ko A, Dumont BL, Han L, Vives L, O'Roak BJ, Sudmant PH, Shendure J, Abney M, Ober C, Eichler EE (2012). Estimating the human mutation rate using autozygosity in a founder population. Nat Genet.

[9] Swope V, Alexander C, Starner R, Schwemberger S, Babcock G, Abdel-Malek ZA (2014). Significance of the melanocortin 1 receptor in the DNA damage response of human melanocytes to ultraviolet radiation. Pigment Cell Melanoma Res.

[10] Rodríguez CI, Setaluri V (2014). Cyclic AMP (cAMP) signaling in melanocytes and melanoma. Arch Biochem Biophys.

[11] Hartman ML, Czyz M (2015). Pro-survival role of MITF in melanoma. J Invest Dermatol.

[12] Lee KM, Chuang E, Griffin M, Khattri R, Hong DK, Zhang W, Straus D, Samelson LE, Thompson CB, Bluestone JA (1998). Molecular basis of T cell inactivation by CTLA-4. Science.

[13] Ito A, Kondo S, Tada K, Kitano S (2015). Clinical Development of Immune Checkpoint Inhibitors. Biomed Res Int.

[14] Khatri P, Sirota M, Butte AJ (2012). Ten years of pathway analysis: current approaches and outstanding challenges. PLoS Comput Biol.

[15] Khatri P, Drăghici S (2005). Ontological analysis of gene expression data: current tools, limitations, and open problems. Bioinformatics.

[16] Tian L, Greenberg SA, Kong SW, Altschuler J, Kohane IS, Park PJ (2005). Discovering statistically significant pathways in expression profiling studies. Proc Natl Acad Sci USA.

[17] Mitrea C, Taghavi Z, Bokanizad B, Hanoudi S, Tagett R, Donato M, Voichiţa C, Drăghici S (2013). Methods and approaches in the topology-based analysis of biological pathways. Front Physiol.

[18] Afsari B, Geman D, Fertig EJ (2014). Learning dysregulated pathways in cancers from differential variability analysis. Cancer Inform.

[19] Ho JWK, Stefani M, dos Remedios CG, Charleston MA (2008). Differential variability analysis of gene expression and its application to human diseases. Bioinformatics.

[20] Eddy JA, Hood L, Price ND, Geman D (2010). Identifying tightly regulated and variably expressed networks by Differential Rank Conservation (DIRAC). PLoS Comput Biol.

[21] Buzdin AA, Zhavoronkov AA, Korzinkin MB, Venkova LS, Zenin AA, Smirnov PY, Borisov NM (2014). Oncofinder, a new method for the analysis of intracellular signaling pathway activation using transcriptomic data. Front Genet.

[22] Buzdin AA, Zhavoronkov AA, Korzinkin MB, Roumiantsev SA, Aliper AM, Venkova LS, Smirnov PY, Borisov NM (2014). The OncoFinder algorithm for minimizing the errors introduced by the high-throughput methods of transcriptome analysis. Front Mol Biosci.

[23] Borisov NM, Terekhanova NV, Aliper AM, Venkova LS, Smirnov PY, Roumiantsev S, Korzinkin MB, Zhavoronkov AA, Buzdin AA (2014). Signaling pathways activation profiles make better markers of cancer than expression of individual genes. Oncotarget.

[24] Lezhnina K, Kovalchuk O, Zhavoronkov AA, Korzinkin MB, Zabolotneva AA, Shegay PV, Sokov DG, Gaifullin NM, Rusakov IG, Aliper AM, Roumiantsev SA, Alekseev BY, Borisov NM (2014). Novel robust biomarkers for human bladder cancer based on activation of intracellular signaling pathways. Oncotarget.

[25] Aliper AM, Frieden-Korovkina VP, Buzdin A, Roumiantsev SA, Zhavoronkov A (2014). Interactome analysis of myeloid-derived suppressor cells in murine models of colon and breast cancer. Oncotarget.

[26] Spirin PV, Lebedev TD, Orlova NN, Gornostaeva AS, Prokofjeva MM, Nikitenko NA, Dmitriev SE, Buzdin AA, Borisov NM, Aliper AM, Garazha AV, Rubtsov PM, Stocking C (2014). Silencing AML1-ETO gene expression leads to simultaneous activation of both pro-apoptotic and proliferation signaling. Leukemia.

[27] Aliper AM, Csoka AB, Buzdin A, Jetka T, Roumiantsev S, Moskalev A, Zhavoronkov A (2015). Signaling pathway activation drift during aging: Hutchinson-Gilford Progeria Syndrome fibroblasts are comparable to normal middle-age and old-age cells. Aging (Albany NY).

[28] Makarev E, Cantor C, Zhavoronkov A, Buzdin A, Aliper A, Csoka AB (2014). Pathway activation profiling reveals new insights into age-related macular degeneration and provides avenues for therapeutic interventions. Aging (Albany NY).

[29] Elder D (1999). Tumor progression, early diagnosis and prognosis of melanoma. Acta Oncol.

[30] Vander Heiden MG, Cantley LC, Thompson CB (2009). Understanding the Warburg effect: the metabolic requirements of cell proliferation. Science.

[31] Cantor JR, Sabatini DM (2012). Cancer cell metabolism: one hallmark, many faces. Cancer Discov.

[32] Ridnour LA, Thomas DD, Donzelli S, Espey MG, Roberts DD, Wink DA, Isenberg JS (2006). The biphasic nature of nitric oxide responses in tumor biology. Antioxid Redox Signal.

[33] Roberts DD, Isenberg JS, Ridnour LA, Wink DA (2007). Nitric oxide and its gatekeeper thrombospondin-1 in tumor angiogenesis. Clin Cancer Res.

[34] Almeida A, Moncada S, Bolaños JP (2004). Nitric oxide switches on glycolysis through the AMP protein kinase and 6-phosphofructo-2-kinase pathway. Nat Cell Biol.

[35] Thomas DD, Espey MG, Ridnour LA, Hofseth LJ, Mancardi D, Harris CC, Wink DA (2004). Hypoxic inducible factor 1alpha, extracellular signal-regulated kinase, and p53 are regulated by distinct threshold concentrations of nitric oxide. Proc Natl Acad Sci USA.

[36] Feun L, You M, Wu CJ, Kuo MT, Wangpaichitr M, Spector S, Savaraj N (2008). Arginine deprivation as a targeted therapy for cancer. Current pharmaceutical design.

[37] Yerushalmi HF, Besselsen DG, Ignatenko NA, Blohm-Mangone KA, Padilla-Torres JL, Stringer DE, Guillen JM, Holubec H, Payne CM, Gerner EW (2006). Role of polyamines in arginine-dependent colon carcinogenesis in Apc(Min) (/+) mice. Mol Carcinog.

[38] Gilmour SK (2007). Polyamines and nonmelanoma skin cancer. Toxicol Appl Pharmacol.

[39] Igarashi K, Sakamoto I, Goto N, Kashiwagi K, Honma R, Hirose S (1982). Interaction between polyamines and nucleic acids or phospholipids. Arch Biochem Biophys.

[40] Gerner EW, Meyskens FL (2004). Polyamines and cancer: old molecules, new understanding. Nat Rev Cancer.

[41] Di Marino D, D'Annessa I, Tancredi H, Bagni C, Gallicchio E (2015). A unique binding mode of the eukaryotic translation initiation factor 4E for guiding the design of novel peptide inhibitors. Protein Sci.

[42] Medina MA, Correa-Fiz F, Rodríguez-Caso C, Sánchez-Jiménez F (2005). A comprehensive view of polyamine and histamine metabolism to the light of new technologies. J Cell Mol Med.

[43] Abildgaard C, Guldberg P (2015). Molecular drivers of cellular metabolic reprogramming in melanoma. Trends in Molecular Medicine.

[44] van Vlies N, Ofman R, Wanders RJA, Vaz FM (2007). Submitochondrial localization of 6-N-trimethyllysine dioxygenase–implications for carnitine biosynthesis. FEBS J.

[45] Monfregola J, Cevenini A, Terracciano A, van Vlies N, Arbucci S, Wanders RJA, D'Urso M, Vaz FM, Ursini MV (2005). Functional analysis of TMLH variants and definition of domains required for catalytic activity and mitochondrial targeting. J Cell Physiol.

[46] Wei YD, Rannug U, Rannug A (1999). UV-induced CYP1A1 gene expression in human cells is mediated by tryptophan. Chem Biol Interact.

[47] Niu X, Zhang T, Liao L, Zhou L, Lindner DJ, Zhou M, Rini B, Yan Q, Yang H (2012). The von Hippel–Lindau tumor suppressor protein regulates gene expression and tumor growth through histone demethylase JARID1C. Oncogene.

[48] Wellmann S, Bettkober M, Zelmer A, Seeger K, Faigle M, Eltzschig HK, Bührer C (2008). Hypoxia upregulates the histone demethylase JMJD1A via HIF-1. Biochemical and Biophysical Research Communications.

[49] Levy C, Khaled M, Iliopoulos D, Janas MM, Schubert S, Pinner S, Chen P-H, Li S, Fletcher AL, Yokoyama S, Scott KL, Garraway LA, Song JS (2010). Intronic miR-211 Assumes the Tumor Suppressive Function of Its Host Gene in Melanoma. Molecular Cell.

[50] Mazar J, Khaitan D, DeBlasio D, Zhong C, Govindarajan SS, Kopanathi S, Zhang S, Ray A, Perera RJ (2011). Epigenetic Regulation of MicroRNA Genes and the Role of miR-34b in Cell Invasion and Motility in Human Melanoma. PLoS ONE.

[51] Shabalin AA, Tjelmeland H, Fan C, Perou CM, Nobel AB (2008). Merging two gene-expression studies via cross-platform normalization. Bioinformatics.

