# Supplementary material for: Molecular pathway activation features linked with transition from normal skin to primary and metastatic melanomas in human
Source: Oncotarget. 2015 Nov 26;7(1):656–70. doi: 10.18632/oncotarget.6394 (PMC4808024; doi:10.18632/oncotarget.6394)
Supplement: Supplementary file 1 [file oncotarget-07-0656-s001.pdf]

Molecular pathway activation features linked with transition from normal skin to primary and metastatic melanomas in human

Supplementary Materials

Supplementary Dataset S1: List of molecular pathways used in this study, including 271 signaling and 321 metabolic intracellular pathways

Supplementary Dataset S2: Pathway activation strength (PAS) values calculated for all the samples under investigation

Supplementary Dataset S3: Full statistical comparison of different classifiers based on all molecular pathways under comparison. Sensitivity, specifity and balanced accuracy values are shown for different classifier models.

Supplementary Dataset S4: Full statistical comparison of different classifiers based only on top molecular pathways. Sensitivity, specifity and balanced accuracy values are shown for different classifier models.

Supplementary Dataset S5: Correlation heatmaps and Jaccard structural similarity index for each cluster of molecular pathways under investigation

Supplementary Dataset S6: List of molecular pathways forming each commonly regulated pathway cluster
