# Supplementary material for: Molecular pathway activation features linked with transition from normal skin to primary and metastatic melanomas in human
Source: Oncotarget. 2015 Nov 26;7(1):656–70. doi: 10.18632/oncotarget.6394 (PMC4808024; doi:10.18632/oncotarget.6394)
Supplement: Supplementary file 2 [file oncotarget-07-0656-s002.docx]

**Supplementary Dataset S1.** Description of signalling and metabolic pathways analysed in this study.

| **Pathway Name** | **Pathway Summary:** | **Reference for Pathway Information:** |
| --- | --- | --- |
| **AHR Main Pathway** | Aryl Hydrocarbon Receptor (AHR) is is a member of the bHLH (basic Helix-Loop-Helix)- PAS (PerARNT-Sim) family of transcriptional regulators. Upon ligand binding, AHR in the complex is activated by a conformation change that exposes a NLS. | PMID: 16545780, PMID: 16153594, PMID: 12354770 |
| **AHR Pathway (AHR Degradation)** | microPathway (chain consisting of 3 nodes) brings to degradation of AHR. AHR is degraded by the Ubiquitin/Proteasome machinery after being exported from the nucleus to the cytoplasm. | PMID: 1620591, PMID: 11368516 |
| **AHR Pathway (C-MycExpression)** | microPathway (chain consisting of 3 nodes) brings to expression C-Myc protein. Direct interactions between AHR and RelA (a NF-KappaB subunit) induce transactivation of c-Myc protein. | PMID: 12181450, PMID: 16091746, PMID: 14560034 |
| **AHR Pathway (Cath-D Expression)** | microPathway (chain consisting of 3 nodes) blocks expression of Cath-D. Activation of the AHR results in transcriptional inhibition of genes such as those encoding the Immunoglobulin heavy-chain and Estrogen-inducible p27, Cathepsin D, and pS2. | PMID: 12354770, PMID: 12024042, PMID:11165043 |
| **AHR Pathway (PS2 Gene Expression)** | microPathway (chain consisting of 3 nodes) blocks expression of PS2 (Gastrointestinal Trefoil Protein-pS2). Activation of the AHR results in transcriptional inhibition of genes encoding the Immunoglobulin heavy-chain, p27, Cath-D and pS2. | PMID: 12354770, PMID: 12024042, PMID:11165043 |
| **AKT Main Pathway** | Akt /Akt /PKB is a Serine/threonine Kinase that is involved in mediating various biological responses.vActivation of Akt can begin with several events, mainly the binding of a Ligand to a Receptor in the cell membrane.PKB is a Serine/threonine Kinase that is involved in mediating various biological responses. | PMID: 12818177, PMID: 12535517 |
| **AKT Pathway (Aggregation & Neurodegeneration)** | microPathway brings to Aggregation and Neurodegeneration. Akt phosphorylates Ataxin1 and modulate neurodegeneration. 14-3-3 protein mediates the neurotoxicity of Ataxin1 by binding to and stabilizing Ataxin1, thereby slowing its normal degradation. | PMID: 12734380, PMID: 15710331 |
| **AKT Pathway (Apoptosis Inhibition)** | microPathway (chain consisting of 3 nodes) brings to inhibition of apoptosis. Akt and PRAS40 are components of the PI3K pathway. This pathway plays a role in glucose uptake, cell growth, and apoptosis inhibition. | PMID: 14645242, PMID: 15799971, PMID: 12524439, |
| **AKT Pathway (Blocks Apoptosis)** | microPathway (chain consisting of 3 nodes) blocks apoptosis. The downstream targets of Akt include XIAP (X-Linked Inhibitor of Apoptosis Protein). | PMID: 12176997 |
| **AKT Pathway (Cardiovascular Homeostasis)** | microPathway (chain consisting of 3 nodes) brings to cardiovascular homeostasis. Akt has been linked to angiogenesis, through the activation of eNOS, which influences long-term blood vessel growth. | PMID: 14523021, PMID:16375650 |
| **AKT Pathway (Caspase Cascade)** | microPathway (chain consisting of 3 nodes) blocks сaspase сascade. Phosphorylation of Caspase9 decreases apoptosis by directly inhibiting the protease activity. | PMID: 14523021, PMID:16375650 |
| **AKT Pathway (Cell Cycle)** | microPathway (chain consisting of 3 nodes) activates cell cycle. Akt also activates Glycogen synthesis by phosphorylating and inactivating GSK3, which leads to the activation of Glycogen Synthase and CyclinD1. | PMID: 14523021, PMID:16375650 |
| **AKT Pathway (Cell Cycle Progression)** | microPathway influences on cell cycle progression. 14-3-3 proteins bind phosphorylated p27 and cause active export from nucleus. Without p27 in the nucleus, the Cyclin-CDK complexes form and promote cell cycle progression. | PMID: 15674326, PMID: 15123687, PMID: 15289327 |
| **AKT Pathway (Cell Survival)** | microPathway (chain consisting of 3 nodes) leads to cell survival. Akt inhibits apoptosis by phosphorylating the BAD component of the BAD/BclXL (Bcl2 Related Protein Long Isoform) complex. | PMID: 14523021, PMID:16375650 |
| **AKT Pathway (Death Genes)** | microPathway (chain consisting of 3 nodes) brings to death genes. In the presence of survival factors, Akt1 phosphorylates FKHRL1, leading to the association of FKHRL1 with 14-3-3 proteins and its retention in the cytoplasm. | PMID: 14523021, PMID:16375650 |
| **AKT Pathway (Elevation of Glucose Import)** | microPathway (chain consisting of 3 nodes) brings to elevation of glucose import. Akt can regulate several levels of Glucose metabolism. It enhances Glucose-uptake in Insulin-responsive tissues; the GLUTs transport glucose into the cell. | PMID: 14523021, PMID:16375650 |
| **AKT Pathway (ERK Pathway)** | microPathway (chain consisting of 3 nodes) activates ERK pathway. The downstream targets of Akt include Raf1 (v-Raf1 Murine Leukemia Viral Oncogene Homolog-1), which is positive regulators of the mTOR-S6K pathway. | PMID: 12176997 |
| **AKT Pathway (Genetic Stability)** | microPathway (chain consisting of 3 nodes) unpromotes genetic stability. Akt impair Chk1 through phosphorylation, ubiquitination, and reduced nuclear localization to promote genomic instability in tumor cells. | PMID: 15710331, PMID: 12734380 |
| **AKT Pathway (Glucose Uptake)** | microPathway (chain consisting of 3 nodes) stimulates glucose uptake. Akt induces glycolysis through the phosphorylation and activation PFK2, which in turn activates PFK1. These enzymes convert Fructose-6-Phosphate into Fructose-1, 6-Bisphosphate. | PMID: 14523021, PMID:16375650 |
| **AKT Pathway (Glycogen Synthesis)** | microPathway (chain consisting of 3 nodes) activates glycogen synthesis. Akt activates Glycogen synthesis by phosphorylating and inactivating GSK3, which leads to the activation of Glycogen Synthase and CyclinD1. | PMID: 14523021, PMID:16375650 |
| **AKT Pathway (Insulin Stimulated Mitogenesis)** | microPathway (chain consisting of 3 nodes) activates Insulin-stimulated mitogenesis. WNK1 is a physiologically relevant target of Insulin signaling through PI3K and Akt and functions as a negative regulator of Insulin-stimulated mitogenesis. | PMID: 15710331, PMID: 15799971, PMID: 12524439 |
| **AKT Pathway (JNK Pathway)** | microPathway blocks JNK pathway. | PMID: 12734380, PMID: 15710331 |
| **AKT Pathway (Neuroprotection)** | microPathway blocks JNK pathway. Akt interacts with the JIP1 (JNK Interacting Protein-1) scaffold and inhibits the ability of JIP1 to form active JNK signaling complexes. Akt can inhibit one or more steps within the JNK signaling pathway. | PMID: 12734380, PMID: 15710331 |
| **AKT Pathway (NF-kB Pathway)** | microPathway (chain consisting of 3 nodes) stimulates neuroprotection. Htt is a substrate of Akt and phosphorylation of Htt by Akt is crucial to mediate the neuroprotective effects of IGF1 (Insulin-Like Growth Factor-I). | PMID: 15710331, PMID: 15799971, PMID: 12524439 |
| **AKT Pathway (p53 Degradation)** | microPathway (chain consisting of 3 nodes) activates NF-kB pathway. Akt activates IKK, which ultimately leads to NF-KappaB activation and cell survival. | PMID: 14523021, PMID:16375650 |
| **AKT Pathway (p73 Mediated Apoptosis)** | microPathway (chain consisting of 3 nodes) brings to degradation of p53. Akt phosphorylation of MDM2 allows its entry into the nucleus where it targets p53 for degradation. | PMID: 15674326, PMID: 15123687, PMID: 15289327, PMID: 11756412 |
| **AKT Pathway (Protein Synthesis)** | microPathway (chain consisting of 3 nodes) blocks Protein Synthesis. Nonphosphorylated PHASI binds to eIF4E (Eukaryotic Initiation Factor-4E) and inhibits protein synthesis. | PMID: 14523021, PMID:16375650 |
| **AKT Pathway (Regeneration of Cyclic Nucleotide)** | microPathway (chain consisting of 3 nodes) operates regeneration of cyclic nucleotide. Akt phosphorylates PDE3B on Ser273. This activates PDE3B and results in regulation of intracellular levels of cyclic nucleotides in response to Insulin. | PMID: 14523021, PMID:16375650 |
| **AKT Pathway (Respiratory Burst)** | microPathway (chain consisting of 3 nodes) stimulates respiratory burst. Akt mediates PI3K-dependent p47Phox phosphorylation, which contributes to respiratory burst activity in human neutrophils. | PMID: 12734380, PMID: 15710331 |
| **AKT Pathway (Survival Genes)** | microPathway (chain consisting of 3 nodes) stimulates survival genes. The transcription factor CREB is directly phosphorylated at Ser133 by Akt. This causes an increased affinity of CREB for its co-activator protein, Crb (Crumbs). | PMID: 15674326, PMID: 15123687, PMID: 15289327, PMID: 11756412 |
| **AKT Pathway (Synaptic Signaling)** | microPathway (chain consisting of 3 nodes) stimulates synaptic signaling. Akt-mediated phosphorylation increases the number of GABA(A)Rs on the plasma membrane surface, thereby increasing the receptor-mediated synaptic transmission in neurons. | PMID: 14645242, PMID: 15799971, PMID: 12524439, |
| **AKT Pathway (Translation)** | microPathway (chain consisting of 3 nodes) stimulates translation. Activation of mTOR results in the phosphorylation of ribosomal protein S6 kinase, p70S6K. Akt also phosphorylates the two tumor suppressor genes TSC1 and TSC2. | PMID: 15710331, PMID: 12734380 |
| **Androgen Receptor Pathway** | The steroidal androgens, testosterone and its metabolite DHT, mediate their biological effects predominantly through binding to the AR (Androgen Receptor), an androgen-inducible member of the nuclear receptor superfamily of transcription factors. | PMID: 12351684, PMID: 12648523 |
| **Androgen Receptor Pathway (Apoptosis)** | microPathway (chain consisting of 3 nodes) blocks apoptosis. | PMID: 12648523 |
| **Androgen Receptor Pathway (Degradation)** | microPathway (chain consisting of 3 nodes) brings to AR degradation. | PMID: 12351684 |
| **Androgen Receptor Pathway (Cell Survival & Cell Growth)** | microPathway (chain consisting of 3 nodes) stimulates cell survival and cell growth. | PMID: 12700179 |
| **Androgen Receptor Pathway (Gonadotropin Regulation)** | microPathway (chain consisting of 3 nodes) are especially important in male gonadotropin regulation. | PMID: 12351684 |
| **Androgen Receptor Pathway (Histone Modification)** | microPathway (chain consisting of 3 nodes) brings to histone modification. | PMID:11714699 |
| **Androgen Receptor Pathway (Prostate Differentiation & Development)** | microPathway (chain consisting of 3 nodes) has impact on prostate differentiation and development. | PMID: 14501770 |
| **Androgen Receptor Pathway (Sexual Differentiation & Sexual Maturation at Puberty)** | microPathway (chain consisting of 3 nodes) has impact on sexual differentiation sexual maturation at puberty | PMID:12700179 |
| **ATM Main Pathway** | ATM is a key regulator of multiple pw which respond to DNA strand breaks induced by damaging agents IR, radiometric agents or by normal processes. These responses involve the activation of cell cycle Chk factors, DNA repair and Apoptosis. | PMID:16411093 |
| **ATM Pathway (Apoptosis)** | microPathway (chain consisting of 3 nodes) stimulates apoptosis. Activated c-Abl may also promote apoptosis via up-regulation of p73, a pro-apoptotic protein and a p53 homolog. | PMID: 16178012, PMID: 15916964, PMID: 12955071 |
| **ATM Pathway (Apoptosis & Senescense)** | microPathway (chain consisting of 3 nodes) brings to apoptosis and senescense. p53 can induce expression of genes that induce apoptosis; and in certain tissues, induction of p53 leads to apoptosis, rather than cell cycle arrest. | PMID: 16178012, PMID: 15916964, PMID: 12955071 |
| **ATM Pathway (Cell Cicle Checkpoint Control)** | microPathway (chain consisting of 3 nodes) carries out cell cycle checkpoint control. ATM also phosphorylates BID (BH3 Interacting Domain Death Agonist). This phosphorylation is required for a downstream function in cell cycle checkpoint control. | PMID: 15073328, PMID: 16122426, PMID:15289825 |
| **ATM Pathway (Cell Survival)** | microPathway influences on cell survival. SAPK activation by c-Abl leads to activation of c-Jun and thus plays an important role in cell survival. Also, SMC1 and histone H2AX, are important for cell survival after irradiation. | PMID: 16178012, PMID: 15916964, PMID: 12955071 |
| **ATM Pathway (Checkpoint Activation)** | microPathway (chain consisting of 3 nodes) brings to checkpoint activation. Hyperphosphorylation of Rad9 induced by IR is also dependent on ATM. Ser(272) of Rad9 is phosphorylated directly by ATM. | PMID: 15073328, PMID: 16122426, PMID:15289825 |
| **ATM Pathway (DNA Repair)** | microPathway (chain consisting of 3 nodes) brings to DNA repair. SMC1 and histone H2AX phosphorylation facilitate DNA repair by means of phosphorylated histone H2AX recruits chromatin remodeling complexes to sites of DNA DSBs. | PMID: 16150728, PMID: 16082221, PMID: 16178012 |
| **ATM Pathway (G2 Mitosis Progression)** | microPathway (chain consisting of 3 nodes) blocks G2_Mitosis_Progression. The inactive CDC25C is incapable of removing an inhibitory phosphate group on Tyr-15 of CDC2, preventing entry into mitosis. | PMID: 16150728, PMID: 16082221, PMID: 16178012 |
| **ATM Pathway (G2/M Checkpoint Arrest)** | microPathway (chain consisting of 3 nodes) brings to G2-M checkpoint arrest. p53 inhibits G2-M transition by repressing the transcription of CDC2 (Cell Division Cycle-2, G1 to S and G2 to M) and Cyclin-B. | PMID: 16150728, PMID: 16082221, PMID: 16178012 |
| **ATM Pathway (MDMX Ubiquitination & Degradation)** | microPathway (chain consisting of 3 nodes) brings to MDMX ubiquitination and Degradation. The mechanism by which phosphorylation enhances MDMX-MDM2 (Mouse Double Minute-2) binding and MDMX ubiquitination by MDM2. | PMID: 16150728, PMID: 16082221, PMID: 16178012 |
| **ATM Pathway (NF-kB Pathway)** | microPathway (chain consisting of 3 nodes) activates NF-kB pathway. ATM also phosphorylates IKappaB-Alpha and thus plays an important role in NF-KappaB activation. | PMID: 16178012, PMID: 15916964, PMID: 12955071 |
| **ATM Pathway (S-Phase Arrest)** | microPathway (chain consisting of 3 nodes) brings to S phase arrest. ATM also phosphorylates FANCD2 (Fanconi Anemia Complementation Group-D2) protein, which then leads to S phase arrest. | PMID: 16178012, PMID: 15916964, PMID: 12955071 |
| **ATM Pathway (S-Phase Progression)** | microPathway (chain consisting of 3 nodes) brings to S-phase progresion. CDC25A activates CDK2 and promotes progression through S phase, while CDC25C activates CDC2 and promotes progression from G2 into mitosis. | PMID: 16150728, PMID: 16082221, PMID: 16178012 |
| **ATM Pathway (Synaptic Vesicle Transport)** | microPathway (chain consisting of 3 nodes) brings to synaptic vesicle transport. ATM interacts with AP3B2, a neuronal homolog of Beta-Adaptin involved in synaptic vesicle transport in neuronal cells. | PMID: 16178012, PMID: 15916964, PMID: 12955071 |
| **cAMP Main Pathway** | cAMP (Cyclic Adenosine 3,5-monophosphate) is second messenger, which has a fundamental role in the cellular response to many extracellular stimuli. cAMP provide the paradigms for the second messenger concept and for signaling compartmentalization. | PMID: 15922020, PMID: 15539636 |
| **cAMP Pathway (Axonal Growth)** | microPathway (chain consisting of 3 nodes) brings to axonal growth. microPathway maintains cardiovascular homeostasis. | PMID: 14715913 |
| **cAMP Pathway (Cell Growth)** | microPathway maintains cardiovascular homeostasis. PKA activation enhances eNOS enzyme activity by phosphorylation of Serine residue in order to stimulate eNOS signaling, which is essential to maintain cardiovascular homeostasis. | PMID: 15539636 |
| **cAMP Pathway (Cell Proliferation)** | microPathway (chain consisting of 3 nodes) brings to cell growth. cAMP-activated mTOR and p70S6K promote cell growth via the mTOR and p70S6K signaling route. | PMID:14715081 |
| **cAMP Pathway (Cell Survival)** | microPathway (chain consisting of 3 nodes) stimulates cell proliferation. Inactivation of PCTK1 (PCTAIRE Protein Kinase-1) helps to maintain control over cell proliferation, thereby degrading cell cycle checkpoint regulators. | PMID: 14715913, PMID: 16214430 |
| **cAMP Pathway (Cell Survival & Chemotaxis)** | microPathway (chain consisting of 3 nodes) brings to cell survival. Rap1A and Rap1B further stimulate Rap1 and Rap2 pathways that are vital for cell survival. | PMID: 16056139, PMID: 16207818 |
| **cAMP Pathway (Cytokines Production)** | microPathway (chain consisting of 3 nodes) brings to cell survival and chemotaxis. cAMP-activated mTOR and p70S6K promote cell growth via the mTOR and p70S6K signaling route, whereas. | PMID:14715081 |
| **cAMP Pathway (Degradation of Cell Cycle Regulators)** | microPathway (chain consisting of 3 nodes) brings to Cytokine production. The Urocortin-cAMP mediated induction of PKC and p38 results in Apoptosis and Cytokine production (like that of IL-6), downstream to the Urocortin-cAMP pathway. | PMID:14715081 |
| **cAMP Pathway (Endothelial Cell Redulation)** | microPathway brings to degradation of cell cycle regulators. Inactivation of PCTK1 and APC helps to maintain control cell proliferation and anaphase initiation and late mitotic events, thereby checking the degradation cell cycle regulators. | PMID: 14715913, PMID: 16214430 |
| **cAMP Pathway (Cardiovascular Homeostasis)** | microPathway brings to endothelial cell regulation. Rho Kinase regulates Myosin-II and cell contraction by catalyzing phosphorylation of the regulatory subunit of Myosin phosphatase, PPtase1, by inhibiting its catalytic activity. | PMID: 16055445 |
| **cAMP Pathway (Glycogen Synthesis)** | microPathway (chain consisting of 3 nodes) brings to glycogen synthesis. PKA inhibits GYS (Glycogen Synthase) leading to seizure of energy consuming process like Glycogen Synthesis. | PMID: 14715913, PMID: 16214430 |
| **cAMP Pathway (Glycolysis)** | microPathway (chain consisting of 3 nodes) brings to glycolysis. The phosphate on C-1 is transferred to the enzyme regenerating it and Glucose-6-Phospahte is the released product that enters Glycolysis. | PMID: 14715913, PMID: 16214430 |
| **cAMP Pathway (Metabolic Energy)** | microPathway brings to release of metabolic energy. Akt activates PDE (Phosphodiesterase) to facilitate the conversion of cAMP to AMP through Akt Signaling. This modulates cardiac contractility and release of metabolic energy. | PMID: 15956717 |
| **cAMP Pathway (Myocardial Contraction)** | microPathway (chain consisting of 3 nodes) operates the process of myocardial contraction. cAMP plays a vital role in regulation of cardiovascular function by controlling the process of myocardial contraction. | PMID: 14715913 |
| **cAMP Pathway (Oncogenesis)** | microPathway (chain consisting of 3 nodes) blocks oncogenesis. Increased cAMP levels promote survival of neuronal cells by inactivating GSK3Alpha and GSK3Beta via a PKA dependent mechanism and thus prevents Oncogenesis. | PMID: 14715913, PMID: 15950778 |
| **cAMP Pathway (Protein Retention)** | microPathway (chain consisting of 3 nodes) brings to protein retention. The activated KDELR promotes retrieval of proteins (protein retention) from golgi complex to endoplasmic reticulum thereby maintaining steady state of the cell. | PMID: 14715913, PMID: 15950778 |
| **cAMP Pathway (Regulation of Cytoskeleton)** | microPathway (chain consisting of 3 nodes) influences on regulation of cytoskeleton. PKA inhibits Adducin action by limiting its role during assembly of Spectrin Actin network in erythrocytes, thereby reducing the chances of Erythroleukemia. | PMID: 14715913, PMID: 15950778 |
| **Caspase Cascade Main** | Caspases are a family of cysteine proteases that act in concert in a cascade triggered by apoptosis signaling. The Caspase cascade is activated by two distinct routes: one from cell surface and the other from mitochondria. | PMID: 9651578 |
| **Caspase Cascade (Activated Tissue Transglutaminase)** | microPathway (chain consisting of 3 nodes) brings to activated tissue trans-glutaminase. | PMID: 9651578 |
| **Caspase Cascade (Apoptosis)** | microPathway (chain consisting of 3 nodes) brings to apoptosis. After activation, down stream Caspases cleave cytoskeletal and nuclear proteins like PARP, DNA-PK, Rb, PAK1, GDID4, Fodrin, Lamin-A, Lamin-B1, Lamin-B2, thus inducing apoptosis. | PMID: 10713717 |
| **Caspase Cascade (Cell Survival)** | microPathway (chain consisting of 3 nodes) brings to cell survival. | PMID: 10713717 |
| **Caspase Cascade (ICAD Degradation)** | microPathway (chain consisting of 3 nodes) brings to ICAD Degradation. Caspase3 cleaves ICAD (Inhibitor of CAD) to free CAD (Caspase-Activated DNase) to cause DNA fragmentation. | PMID: 10713717 |
| **CD40 Main Pathway** | CD40, a TNFR family member, conveys signals regulating diverse cellular responses, ranging from proliferation and differentiation to growth suppression and cell death. | PMID: 10891490, PMID: 10764746 |
| **CD40 Pathway (Cell Survival)** | microPathway (chain consisting of 3 nodes) brings to cell survival. CD40 stimulation include the JAK3 (Janus family of kinases)-STAT3 and PI3K (Phosphatidyl Inositol 3-Kinase) - Akt, which may contribute to the antiapoptotic properties. | PMID: 10085038 |
| **CD40 Pathway (Gene Expresion)** | microPathway brings to gene expression. The activation of multiple pathways including NF-KappaB, MAPK and STAT3 regulate gene expression through activation of Activating Proteins, c-Jun, ATF2 and Rel transcription factors. | PMID:11830667 |
| **CD40 Pathway (IKBs Degradation)** | microPathway brings to IKBs degradation. Act1 (NF-KappaB Activator-1) functions as an adapter, linking TRAF proteins to TAK1/ IKK to activate NF-KappaB /I-KappaB (Inhibitor of Kappa Light Chain Gene Enhancer in B-Cells). | PMID:11830667 |
| **Cellular Anti Apoptosis Main Pathway** | There are broad pathways that lead to Apoptosis. In both pathways, signaling results in the activation of a family of Cys (Cysteine) Proteases, named Caspases that act in a proteolytic cascade to dismantle and remove the dying cell. | PMID: 12181741 |
| **Cellular Anti Apoptosis Pathway (Apoptosis)** | microPathway (chain consisting of 3 nodes) brings to apoptosis. Death Receptors are Cell surface Receptors that transmit Apoptotic signals initiated by specific ligands and play a central role in instructive Apoptosis. | PMID: 15350837 |
| **Cellular Anti Apoptosis Pathway (Depolarization)** | microPathway brings to depolarization. p53 also transactivates other genes that may contribute to Apoptosis including PTEN, APAF1, Perp, p53AIP1 (p53-regulated Apoptosis-Inducing Protein-1), and genes that lead to increases in ROS. | PMID: 12972501 |
| **Chemokine Main Pathway** | Chemokines participate in the growth, differentiation, and activation of leukocytes as well as stimulate various effector functions of these cells, such as integrin activation, chemotaxis, superoxide radical production and granule enzyme release. | PMID: 9649701 |
| **Chemokine Pathway (Cell Activation)** | microPathway (chain consisting of 3 nodes) brings to cell activation. | PMID: 9649701 |
| **Chemokine Pathway (Gene Expression & Apoptosis)** | microPathway (chain consisting of 3 nodes) brings to Gene_Expresion_&_Apoptosis. | PMID:10807766 |
| **Chemokine Pathway (Internalization Degradation Recycling)** | microPathway (chain consisting of 3 nodes) stimulates to internalization degradation recycling. | PMID: 9649701 |
| **Chromatin Main Pathway** | Chromatin assembly involves the formation of nucleosomes from histones and DNA, whereas chromatin remodeling involves the disruption and re-formation of histone-DNA contacts. | PMID: 10839822, PMID: 10397708 |
| **Chromatin Pathway (Octamer Sliding)** | microPathway (chain consisting of 3 nodes) brings to octamer sliding. | PMID: 10397708 |
| **Chromatin Pathway (Octamer Transfer)** | microPathway (chain consisting of 3 nodes) brings to octamer transfer. | PMID: 10839822 |
| **Circadian Main Pathway** | The mammalian circadian system is critical for the proper regulation of behavioral and physiological rhythms. The central oscillator, or master clock, is located in the hypothalamic SCN (Suprachiasmatic Nucleus). | PMID: 12015981, PMID: 16281023 |
| **CREB Main Pathway** | The activation of the nuclear transcription factor CREB (cAMP Responsive Element Binding Protein) belongs to bZIP class of transcription factors that functions in vivo to regulate the proliferation of pituitary cells and thymocytes. | PMID: 16266283 |
| **CREB Branch Gene Expression Pathway** | microPathway stimulates gene expression. CBP/p300 stimulates gene expression by interacting with components of the general transcriptional machinery or by promoting the acetylation of specific lysine residues in nucleosomes. | PMID: 15240013 |
| **Cytokine Main Pathway** | Cytokines are pleiotropic in their biological activities and play pivotal roles in a variety of responses, including the immune response, hematopoiesis, neurogenesis, embryogenesis, and oncogenesis. | PMID:11687446, PMID: 9716487 |
| **DDR pathway Apoptosis** | In normal cells, DNA damage leads to activation of cell cycle checkpoints and arrest in the G1 and G2-phases of the cell cycle. 14-3-3 proteins control the biological activity of several key cell cycle checkpoint proteins. | PMID: 9278512, PMID: 10523629 |
| **DDR Main pathway** | In normal cells, DNA damage leads to activation of cell cycle checkpoints and arrest in the G1 and G2-phases of the cell cycle. 14-3-3 proteins control the biological activity of several key cell cycle checkpoint proteins. | PMID: 10523629, PMID: 9923681 |
| **DNA Repair Mechanisms Pathway** | DNA repair enzymes continuously monitor chromosomes to correct damaged nucleotide residues generated by these exogenous and endogenous agents and exposure to carcinogens and cytotoxic compounds. | PMID: 11357144 |
| **EGFR Main Pathway** | EGFR is a member of the ErbB (Erythroblastic Leukemia Viral Oncogene Homolog) family receptors, a subfamily of four closely related receptor tyrosine kinases: EGFR (ErbB1), Her2/c-Neu (ErbB2), Her3 (ErbB3) and Her4 (ErbB4). | PMID: 16435094, PMID: 16595133 |
| **ErbB Family Main Pathway** | The ErbB family includes four members, EGFR (EGF Receptor)/ErbB1/Her1 (Heregulin-1), ErbB2/Her2 (Heregulin-2), ErbB3/Her3 (Heregulin-3), and ErbB4/Her4 (Heregulin-4). ErbB1 and ErbB2 are involved in the development of many types of human cancer. | PMID: 15077147, PMID: 16442236 |
| **ErbB Family Pathway (Anti-Apoptosis)** | microPathway (chain consisting of 3 nodes) blocks apoptosis. The downstream targets of Akt include XIAP (X-Linked Inhibitor of Apoptosis Protein). Activation of PI3K-Akt pathway results in enhanced antiapoptotic and prosurvival signals, through inhibition of the proapoptotic proteins BAD, GSK3, and the transcription factor FKHR-L1. | PMID: 15972258 |
| **ERK Signaling Main Pathway** | ERK, have been established as major participants in the regulation of cell growth and differentiation, but when improperly activated contribute to malignant transformation. ERK1 and ERK2 form a central component in the MAPK cascade. | PMID: 15077147, PMID: 16442236 |
| **Erythropoeitin Main Pathway** | Erythropoiesis is a major pathway for Erythrocyte production, by which pluripotent Hematopoietic Stem Cells give rise to mature end stage cells via a series of differentiations. | PMID: 16507992, PMID: 17467346, PMID: 15149544, PMID: 15927257 |
| **Estrogen Main Pathway** | The physiological effects of estrogens are mediated by the intracellular ERs (Estrogen Receptors), which regulate transcription of target genes through binding to specific DNA target sequences. | PMID: 11559590, PMID: 10732321 |
| **Fas Signaling Pathway (Negative)** | Fas (Apo1 or CD95) is a death domain-containing member of the TNFR superfamily. It has a central role in the physiological regulation of apoptosis and has been implicated in the pathogenesis of various malignancies and diseases of the immune system. | PMID: 16129431, PMID: 16687251 |
| **Fas Signaling Pathway (Positive)** | Fas (Apo1 or CD95) is a death domain-containing member of the TNFR superfamily. It has a central role in the physiological regulation of Apoptosis and has been implicated in the pathogenesis of various malignancies and diseases of the immune system. | PMID: 16129431, PMID: 16703261 |
| **FLT3 Main Pathway** | The ligand for FLT3 (FLT3L) is expressed by marrow stromal cells and other cells and synergize with other growth factors to stimulate proliferation of stem cells, progenitor cells, dendritic cells, and natural killer cells. | PMID: 7507245, PMID: 8618433, PMID: 8590775 |
| **G-protein Pathway (Ras family GTPases)** | The Tubby protein is the founding member of a multigene protein family that plays an important role in maintenance and function of neuronal cells during development and post-differentiation. | PMID: 10591637, PMID: 11000483, PMID: 9096357 |
| **Glucocorticoid Receptor Main Pathway** | The Glucocorticoids have the ability to inhibit all stages of the inflammatory response. They have an essential role in cell metabolism and raise the level of blood glucose by stimulating gluconeogenesis in the liver. | PMID: 10837061, PMID: 12650700, PMID: 12551744 |
| **Glucocorticoid Receptor Pathway (Cell cycle arrest)** | microPathway brings to Cell Cycle Arrest. The antiproliferative effect of Glucocorticoids is mediated by transcription factors the GR and CEBP-Alpha which induce the synthesis of p21(CIP1) (Cyclin Dependent Kinase Inhibitor-p21). | PMID: 15746257 |
| **Glucocorticoid Receptor Pathway (Cell cycle progression)** | microPathway (chain consisting of 3 nodes) brings to Glucocorticoids increase DNA-binding activity of CEBPbeta via post-translational mechanisms involving phosphorylation. | PMID: 15528271 |
| **Glucocorticoid Receptor Pathway (Gene expression)** | Glucocorticoids bind to the intracellular GR (Glucocorticoid Receptor) that translocates to the nucleus, that modulates gene expression through diverse mechanisms. | PMID: 12650700 |
| **Glucocorticoid Receptor Pathway (Inflammatory cytokines)** | microPathway (chain consisting of 3 nodes) stimulates inflammatory cytokines. Glucocorticoids are important mediators of the immune system and modulate the biological activities of inflammatory cytokines. | PMID: 15331759, PMID: 15488991, PMID: 16113443 |
| **Glucocorticoid Receptor Pathway (SMAD signaling)** | microPathway (chain consisting of 3 nodes) blocks SMAD signaling. GR inhibits transcriptional activation by both Smad3 and Smad4 C-terminal activation domains. | PMID: 15295049 |
| **GPCR Main Pathway** | The principal function of GPCRs is a transmit information about the extracellular environment to the interior of the cell, and they do this by interacting with the G-Proteins. GPCRs recognize a variety of ligands and stimuli. | PMID: 12944098, PMID: 11836477 |
| **GPCR Pathway (Gene expression)** | microPathway (chain consisting of 3 nodes) regulates gene expression. ERK phosphorylate /activate transcription factors (like Elk1 ) and leads to changes in gene expression and cell cycle progression. | PMID: 10836144 |
| **Growth Hormone Main Pathway** | GH (Growth Hormone) and IGF1 (Insulin-Like Growth Factor-1) are two important anabolic hormones that regulate metabolic processes including protein synthesis in almost all tissues throughout the lifespan of mammals. | PMID: 11701436, PMID: 11181952 |
| **Growth Hormone Pathway (Cell survival)** | microPathway (chain consisting of 3 nodes) brings to cell survival. PI3K activates the Akt/PKB Pathway through PDK-1 (Phosphoinositide Dependent Kinase-1) that culminates in cell survival. | PMID: 8815791 |
| **Growth Hormone Pathway (Gene expression)** | microPathway (chain consisting of 3 nodes) brings to gene expression. Signaling molecules (MAPKs, IRS1, PI3K, DAG, PKC, Ca2+, and STATs) contribute to the GH-induced changes in enzymatic activity, transport function, and gene expression. | PMID: 8815791 |
| **Growth Hormone Pathway (Glucose uptake)** | microPathway (chain consisting of 3 nodes) brings to glucose uptake. Activation of PI3K and IRS1 by GH signaling results in increased glucose uptake by effecting the translocation of GLUT4 (Glucose Transporter Protein-4). | PMID: 8815791 |
| **Growth Hormone Pathway (Protein synthesis)** | microPathway (chain consisting of 3 nodes) activates protein synthesis. GH is an anabolic hormone that induces positive nitrogen balance in intact animals and protein synthesis in muscle. | PMID: 11701436 |
| **GSK3 Main Pathway** | GSK3 is involved in signal transduction cascades involving cellular processes, ranging from glycogen metabolism, cell development, gene transcription, protein translation to cytoskeletal organization, cell cycle regulation and apoptosis. | PMID: 12615961, PMID: 11861647 |
| **GSK3 Pathway (Degradation)** | microPathway (chain consisting of 3 nodes) brings to degradation. Cadherins help GSK3 to efficiently phosphorylate the signaling molecule Beta-Ctnn, thus targeting it for subsequent proteasomal degradation. | PMID: 16326393, PMID: 12039794 |
| **GSK3 Pathway (Gene expression)** | microPathway activates gene expression. Stabilization of Beta-Ctnn is associated with its translocation to the nucleus in presence of PP2A where it interacts with members of the LEF/TCF and activates specific target genes. | PMID: 16326393 |
| **GSK3 Pathway (Translation)** | microPathway blocks translation. GSK3 phosphorylates a variety of substrates, Glycogen Synthase and other metabolic enzymes, transcription factors CBP, c-Myc and c-Jun, and the translation initiation factors eIF2 and eIF2B. | PMID: 11094086, PMID: 16326393 |
| **Hedgehog Main Pathway** | microPathway blocks translation. GSK3 phosphorylates a variety of substrates, Glycogen Synthase and other metabolic enzymes, transcription factors CBP, c-Myc and c-Jun, and the translation initiation factors eIF2 and eIF2B. | PMID: 11731473, PMID: 10660040 |
| **Hedgehog Pathway (Repression of target genes(Hh & BMP))** | microPathway (chain consisting of 3 nodes) brings to repression of target genes (Hh, BMP). SUFU interacts directly with Ci proteins, repressing Hh signaling. | PMID: 11493558 |
| **Hedgehog Pathway (Target genes(WNT & Ptc & BMP))** | The vertebrate Hh family is represented by at least three members: Dhh, Ihh and Shh, two Patched homologs, Ptc1 (Patched-1) and Ptc2 (Patched-2); and three homologs of Ci (Cubitus interruptus, a 155 kDa cytoplasmic zinc finger protein) | PMID: 10660040 |
| **HGF Main Pathway** | HGF (Hepatocyte Growth Factor)/SF (Scatter Factor) mediates epithelial-mesenchymal interactions with mitogenic, motogenic and morphogenic activities towards many normal and neoplastic epithelial cells. | PMID: 15686627, PMID: 11046147 |
| **HGF Pathway (Anoikis)** | microPathway (chain consisting of 3 nodes) blocks anoikis. Anoikis is strongly suppressed by HGF through ERK and Akt-signaling pathway, because tumor cells lose matrix attachment during metastasis. | PMID: 11257710 |
| **HGF Pathway (Cell adhesion and cell mirgation)** | microPathway brings to cell adhesion and cell mirgation. The activation of FAK induces the formation of focal adhesions, increased cell motility , and Paxillin phosphorylation may also alter cell adhesion of Met transformed cells. | PMID: 14685170 |
| **HGF Pathway (Cell cycle progression)** | microPathway (chain consisting of 3 nodes) brings to cell cycle progression. Activation of various transcription factors by HGF induces expression of several genes, involved in cell survival and cell cycle progression. | PMID: 11994287 |
| **HGF Pathway (Cell polarity & motility)** | microPathway (chain consisting of 3 nodes) stimulates cell polarity and motility. Activation of the Rac1 pathway and the CDC42 pathway contributes to the regulation of cytoskeleton, thus culminating in cell polarity and cell motility. | PMID: 12393863 |
| **HGF Pathway (Cell scattering)** | microPathway (chain consisting of 3 nodes) activates cell scattering. The PI3K pathway is responsible for cell scattering by inducing the loss of intercellular junctions and cell migration. | PMID: 11502202 |
| **HGF Pathway (Cell survival)** | microPathway (chain consisting of 3 nodes) brings to cell survival. Activation of Rac1 by HGF also contributes to cell survival and differentiation by activating the MEKK (MAP/ERK Kinase Kinases)-->MEK4/7-->JNK (c-Jun Kinase) pathway. | PMID: 12393863 |
| **HGF Pathway (IP3 pathway)** | microPathway activates IP3 (Inositol 1,4,5-Trisphosphate) pathway. Activation of the Met receptor also results in an increase in receptor-mediated activation of PLC-Gamma which catalyzes the generation of IP3 and DAG from PIP2. | PMID: 11766356 |
| **HGF Pathway (PKC pathway)** | microPathway (chain consisting of 3 nodes) activates PKC pathway. DAG (Diacylglycerol) from PIP2 acts as second messenger molecules to mobilize intracellular Calcium and activate PKC (Protein Kinase-C) respectively. | PMID: 11766356 |
| **HIF1-Alpha Main Pathway** | HIF1 consists of a heterodimer of two basic helix-loop-helix PAS proteins, HIF1Alpha and HIF1Beta. HIF1Alpha is an important mediator of the hypoxic response of tumor cells and controls the up-regulation of a number of factors. | PMID: 9235919, PMID: 9890965 |
| **HIF1Alpha Pathway (Gene expression)** | microPathway activates gene expression. Under hypoxic conditions the HIF1Alpha subunits translocate to the nucleus, where they interacts with cofactors CBP /p300 and the Pol II complex to bind to HREs and activate transcription of target genes. | PMID: 9359410 |
| **HIF1Alpha Pathway (HIF1alpha degradation)** | microPathway (chain consisting of 3 nodes) controls degradation of HIF1alpha. The degradation of HIF1Alpha by p53 in a hypoxic condition is inhibited by direct interaction with the JAB1 and the ODD domain by blocking the interaction with p53. | PMID: 9359410 |
| **HIF1Alpha Pathway (NOS pathway)** | microPathway (chain consisting of 3 nodes) activates NOS pathway. HIF1Alpha activates transcription of NOS (Nitric Oxide Synthase), which promotes angiogenesis and vasodilation. | PMID: 9359410 |
| **HIF1Alpha Pathway (p53 Hypoxia pathway)** | microPathway activates p53 Hypoxia pathway. p53 directly interacts with HIF1Alpha and limits hypoxia-induced expression of HIF1Alpha by promoting MDM2- mediated ubiquitination and proteasomal degradation under hypoxic conditions. | PMID: 11516994 |
| **HIF1Alpha Pathway (Pyruvate)** | microPathway (chain consisting of 3 nodes) activates pyruvate. | PMID: 11516994 |
| **HIF1Alpha Pathway (VEGF pathway)** | microPathway activates VEGF pathway. HIF1Alpha is an important mediator of the hypoxic response of tumor cells and controls the up-regulation of a number of factors important for solid tumor expansion including the angiogenic factor VEGF. | PMID: 9359410 |
| **Hypoxia pathway EMT 1** |  | PMID: 25197976 |
| **Hypoxia pathway EMT 2** |  | PMID: 25197976 |
| **Hypoxia pathway EMT 3** |  | PMID: 25197976 |
| **Hypoxia pathway EMT 4** |  | PMID: 25197976 |
| **IGF1R Main Pathway** | IGF1R plays a pivotal role in tissue homeostasis, regulating cell proliferation, differentiation and migration during development and in the adults. Activation of the IGF1R is a particularly important survival-promoting signal. | PMID: 16370142, PMID: 12175651 |
| **IGF1R Signaling Pathway (Cell survival)** | microPathway (chain consisting of 3 nodes) brings to cell survival. The CalmKs activate the transcription factor CREB, and has also been suggested to activate the ERK1/2 pathway, ultimately promoting the process of IGF1R-induced cell survival. | PMID: 12444011, PMID: 12082100, PMID: 15961082 |
| **IGF1R Signaling Pathway (Glucose uptake)** | microPathway (chain consisting of 3 nodes) stimulates glucose uptake. These PKCs, along with Akt enhance the rate of Glucose uptake by the cell by facilitating the GLUT4 (Glucose Transporter-4) translocation from the GLUT4 vesicle to the membrane . | PMID: 15961082 |
| **IGF1R Signaling Pathway (Glycogen synthesis)** | microPathway (chain consisting of 3 nodes) brings to glycogen synthesis. GSK3 promotes the dephosphorylation and activation of Glycogen Synthase, contributing to the stimulation of glycogen synthesis. | PMID: 12388420 |
| **IGF1R Signaling Pathway (IKB degradation)** | microPathway brings to IKB degradation. Induced activity of Akt leads to expression of the anti-apoptotic transcription factor NF-KappaB through regulation of IKKs (I-KappaB Kinases). This results in I-KappaB degradation. | PMID: 15961082 |
| **IGF1R Signaling Pathway (Protein synthesis)** | microPathway activates protein synthesis. IGF1R, by activating eIF4E (Eukaryotic Initiation Factor-4E) and by inhibiting GSK3, stimulates the dephosphorylation and activation of eIF2B, contributing to an increased rate of protein synthesis. | PMID: 12388420 |
| **IL-10 Main Pathway** | IL-10 (Interleukin-10) is a pleiotropic cytokine with important immunoregulatory functions whose actions influence activities of many of the cell-types in the immune system. It is a cytokine with potent anti-inflammatory properties. | PMID: 10433356 |
| **IL-10 Pathway (Gene expression)** | microPathway (chain consisting of 3 nodes) activates gene expression. Homodimerizes and translocates to the nucleus where it binds with high affinity to SBE ( STAT-Binding Elements) in the promoters of various IL-10-responsive genes. | PMID: 11447117 |
| **IL-10 Pathway (Stability determination)** | microPathway (chain consisting of 3 nodes) influences on stability determination. TTP (TNF-ARE Binding Protein Regulating-TNF Biosynthesis) exerts a potent destabilizing activity on TNF-mRNA without imposing translational control. | PMID: 11777539 |
| **IL-10 Pathway (Translational modulation)** | microPathway (chain consisting of 3 nodes) carries out translational modulation. Two RNA binding proteins that have recently been demonstrated to have TNF-ARE binding capacities are TiaR and Tia1. | PMID: 11875494, PMID: 11777539 |
| **IL-2 Main Pathway** | IL-2 (Interleukin-2) is a T-Cell-derived cytokine important in the regulation of growth and differentiation of T-Cells, B-Cells, natural killer cells, glioma cells, and cells of the monocyte lineage after specifically interacting with its receptors. | PMID: 3131876, PMID: 9159211, PMID: 12525482 |
| **IL-2 Pathway (Actin reorganization)** | microPathway (chain consisting of 3 nodes) activates actin reorganization. PI3K (Phosphoinositide-3 Kinase) and Akt/PKB (Protein Kinase-B) mediated signaling pathway are also involved in IL-2 - promoted regulation of actin cytoskeleton organization. | PMID: 11917142, PMID: 9159211 |
| **IL-2 Pathway (Apoptosis)** | microPathway (chain consisting of 3 nodes) activates apoptosis. | PMID: 9159211 |
| **IL-2 Pathway (Apoptosis inhibition)** | microPathway (chain consisting of 3 nodes) blocks apoptosis. CD132 protein mediates an anti-apoptotic signaling pathway through Akt which cooperates with signals from its partner chain, IL-2R beta. | PMID: 9159211, PMID: 3131876 |
| **IL-2 Pathway (Protein synthesis)** | microPathway (chain consisting of 3 nodes) activates protein synthesis. | PMID: 3131876, PMID: 12525482 |
| **IL-6 Main Pathway** | IL-6 provokes a broad range of cellular and physiological responses, including the immune response, inflammation, hematopoiesis, and oncogenesis by regulating cell growth, gene activation, proliferation, survival, and differentiation. | PMID: 9716487, PMID: 10851053 |
| **IL-6 Pathway (IKBs degradation)** | microPathway (chain consisting of 3 nodes) activates degradation of IKBs. | PMID: 9497331, PMID: 9388184 |
| **ILK Main Pathway** | ILK is the major regulator of Integrin mediated signaling. The main function of ILK is to connect Integrins to the cytoskeleton. ILK recruits other adaptor molecules into a large complex to regulate Actin dynamics and Integrin function. | PMID: 12884912, PMID: 16493410, PMID: 12960424 |
| **ILK Pathway (Apoptosis)** | microPathway (chain consisting of 3 nodes) blocks apoptosis. Akt suppresses apoptosis and promote survival by inhibiting BAD (Bcl2-Antagonist of Cell Death), Caspase-3/9 and cell cycle transition by blocking proteolysis of CcnD1 (Cyclin-D1). | PMID: 12778079 |
| **ILK Pathway (Cell adhesion cell motility & opsonization)** | microPathway brings to cell adhesion, cell motility and opsonization. Mesenchymal markers such as Vim and Fn are up-regulated and re-distributed; these regulate wound healing, cell adhesion, cell motility and Opsonization. | PMID: 12884912, PMID: 16493410 |
| **ILK Pathway (Cell cycle proliferation)** | microPathway carries out cell cycle proliferation. Binding of cells to ECM components via Beta1-Integrins suppresses apoptosis and promote survival by inhibiting BAD, Caspase-3/9 and cell cycle transition by blocking proteolysis of CcnD1. | PMID: 12884912, PMID: 16493410, PMID: 12778079 |
| **ILK Pathway (Cell migration/retraction)** | microPathway (chain consisting of 3 nodes) activates cell migration and retraction. Pinch binds to RSU1 and T-Beta4 to influence JNK signaling and cell migration/survival/adhesion (G-Actin polymerization). | PMID: 16493410 |
| **ILK Pathway (Cell motility)** | microPathway (chain consisting of 3 nodes) activates cell motility. Mesenchymal markers such as Vim (Vimentin) and Fn (Fibronectin) are up-regulated and re-distributed; these regulate wound healing, cell adhesion, cell motility and opsonization. | PMID: 12884912, PMID: 16493410 |
| **ILK Pathway (Cytoskeletal reorganization)** | microPathway (chain consisting of 3 nodes) activates cytoskeletal reorganization.TESK1 mainly phosphorylate Cfl (Cofilin) and induce Actin cytoskeletal reorganization, whereas, Hic5 regulates Apoptosis. | PMID: 16314921 |
| **ILK Pathway (G2-phase arrest)** | microPathway (chain consisting of 3 nodes) brings G2-phase arrest. Irradiation is able to activate this pathway, which then does not stimulate proliferation but rather blocks cells in the G2-Phase possibly allowing damage repair. | PMID: 12778079, PMID: 15630415 |
| **ILK Pathway (Induced cell proliferation)** | microPathway (chain consisting of 3 nodes) induces cell proliferation. | PMID: 12884912, PMID: 16493410 |
| **ILK Pathway (Regulation of intermediate filaments)** | microPathway (chain consisting of 3 nodes) regulates intermediate filaments. The epithelial markers are essential for regulation of junction assembly at desmosomes, regulation of intermediate filaments and cell adhesion. | PMID: 12884912, PMID: 16493410 |
| **ILK Pathway (Regulation of junction assembly of desmosomes)** | microPathway regulates junction assembly of desmosomes. With regard to EMT, in addition to E-Cadherin, SNAI/Slug down-regulate epithelial markers- Dsp (Desmoplakin), the epithelial Muc1 (Mucin-1) and Krt18 (Keratin-18)/Cytokeratin-18. | PMID: 12884912, PMID: 16493410 |
| **ILK Pathway (Wound healing)** | microPathway regulates wound healing. Mesenchymal markers such as Vim (Vimentin) and Fn (Fibronectin) are up-regulated and re-distributed; these regulate wound healing, cell adhesion, cell motility and Opsonization. | PMID: 12884912, PMID: 16493410 |
| **Integrin SIgnaling Main Pathway** | The major groups of proteins which regulate cell survival, growth, differentiation, migration, inflammatory responses, platelet aggregation, tissue repair and tumor invasion, are a family of cell surface receptors known as Integrins. | PMID: 11944032, PMID: 9779984, PMID: 9325334 |
| **Integrin SIgnaling Pathway (Cell survival)** | microPathway stimulates сell survival. PI3K is also associated with Integrin-associated focal adhesion complexes and provides protective signal acting through Akt/PKB (Protein Kinase-B) which blocks entry into apoptosis. | PMID: 9779984 |
| **Integrin SIgnaling Pathway (Cytoskeleton contraction integrin modulation cell invasion and migration)** | microPathway regulates cell invasion and migration. Activated Rac with activated CDC42 regulate numerous biochemical pathways, including activation of MEKKs, PAK, MEKs, Vav, and JNK, the key regulators of gene expression and cell cycle. | PMID: 9779984 |
| **Integrin SIgnaling Pathway (Focal adhesion and stress fibers)** | microPathway regulates focal adhesion and stress fibers. The increase in PIP2 synthesis by RhoA is potentially relevant to focal adhesion assembly because the actin binding activity of several cytoskeletal proteins is modulated by PIP2. | PMID: 9779984, PMID: 12040184 |
| **Integrin SIgnaling Pathway (Translocates to the nucleus)** | microPathway translocates to the nucleus. When Integrins are mechanically stressed, the complex stimulates Gs-mediated up-regulation of the cAMP cascade through AC, resulting in nuclear translocation of the catalytic subunit of PKA. | PMID: 12040184, PMID: 11114741 |
| **Interferon Main Pathway** | Once the virus has invaded the cell, a host defense-mediated response is triggered which involves the induction of a family of pleiotropic cytokines known as the IFNs (Interferons). | PMID: 11313713, PMID: 15546383, PMID: 12496969, PMID: 12483210, PMID: 15236653, PMID: 15271478 |
| **Interferon Pathway (Gene expression)** | microPathway (chain consisting of 3 nodes) activates gene expression. The specific transcription factors that are regulated by p38s include CREB (cAMP Responsive Element Binding protein) and Histone-H3. | PMID: 12481403 |
| **Interferon Pathway (Transcription)** | microPathway (chain consisting of 3 nodes) regulates transcription. After phosphorylation by JAKs, the activated STATs form homodimers or heterodimers that translocate to the nucleus and initiate transcription. | PMID: 12040185, PMID: 15864272, PMID: 16110316, PMID: 15829276 |
| **Interferon Pathway (Translation)** | microPathway (chain consisting of 3 nodes) regulates translation. PI3K is activated and regulates downstream activation of mTOR (Mammalian Target of Rapamycin) which mediates the initiation of mRNA translation. | PMID: 12395928 |
| **IP3 Main Pathway** | IP3 is a molecule that functions to transfer a chemical signal received by the cell, such as from a hormone, neurotransmitters, growth factors and hypertrophic stimuli (AngII, ET1, etc.) to various signaling networks within the cell. | PMID: 14761954 |
| **IP3 Pathway (Gene expression)** | microPathway (chain consisting of 3 nodes) regulates gene expression. HDAC export allows MEF2 to activate transcription by recruiting other Ca2+-sensitive transcriptional factors such as NFAT and transcriptional coactivators such as p300. | PMID: 16195467, PMID: 16104587 |
| **JAK mStat Main Pathway** | The JAK -STAT (Signal Transducer and Activator of Transcription) cascade is a pathway mediating the transduction of information between cells are essential for development, cellular differentiation, homeostasis, proliferation and haematopoiesis. | PMID: 16260187, PMID: 16142855 |
| **JAK mStat Pathway (Akt pathway)** | microPathway (chain consisting of 3 nodes) activates Akt pathway. | PMID: 16263337, PMID: 16191414 |
| **JAK mStat Pathway (JAK degradation)** | microPathway (chain consisting of 3 nodes) brings to JAK degradation. The SOCS Box couples the SOCS and associates target proteins JAKs to the proteasomal protein degradation pathway. | PMID: 16532038, PMID: 16266868, PMID: 16426581 |
| **JNK Main Pathway** | Members of the JNK/SAPK family of MAPKs are strongly stimulated by numerous Environmental Stresses, but also more modestly stimulated by Mitogens, Inflammatory Cytokines, Oncogenes, and inducers of Cell differentiation and morphogenesis. | PMID: 12750372, PMID: 16455978 |
| **JNK Pathway (Apoptosis Inflammation Tumorigenesis Cell Migration)** | microPathway (chain consisting of 3 nodes) brings to apoptosis inflammation tumorogenesis cell migration. JNK phosphorylates Serine 178 on Paxillin and regulate cell migration. | PMID: 16601071, PMID: 15950217, PMID: 16682809 |
| **JNK Pathway (Insulin signaling)** | microPathway (chain consisting of 3 nodes) regulates insulin signaling. JNK also regulates Insulin signaling by negatively regulating IRS1 (Insulin Receptor Substrate-1). | PMID: 16601071, PMID: 15950217, PMID: 16682809 |
| **MAPK Family Main Pathway** | MAPKs deliver extracellular signals from activated receptors to various cellular compartments, where they direct the execution of appropriate genetic programs (activation of gene transcription, protein synthesis, cell death, and differentiation). | PMID: 10082509, PMID: 12604570 |
| **MAPK Family Pathway (Chromatin Remodeling)** | microPathway (chain consisting of 3 nodes) brings to chromatin remodeling. The p38 subfamily is involved in affecting cell motility, transcription and chromatin remodeling. | PMID: 14511403 |
| **MAPK Family Pathway (Cytoskeleton)** | microPathway (chain consisting of 3 nodes) operates cytoskeleton. | PMID: 14511403, PMID: 10082509 |
| **MAPK Family Pathway (Gene Expression)** | microPathway (chain consisting of 3 nodes) activates gene expression. JNK-regulated transcription factors help to regulate gene expression in response to a variety of cellular stimuli, including stress events, growth factors and Cytokine. | PMID: 12604570 |
| **MAPK Family Pathway (IKBs Degradation)** | microPathway (chain consisting of 3 nodes) brings to IKBs degradation. p38 MAPK activation by TNF proceeds independently of the TRAF2 -associated NIK (NF-KappaB-Inducing Kinase), an additional MEKK, which binds and activates IKKs (I-KappaB Kinases). | PMID: 14511403 |
| **MAPK Family Pathway (Translation)** | microPathway (chain consisting of 3 nodes) regulates translation. | PMID: 14516729, PMID: 11274345 |
| **MAPK Signaling Main Pathway** | The MAPKs are a group of protein Serine/threonine Kinases that are activated in response to a variety of extracellular stimuli and mediate signal transduction from the cell surface to the nucleus. | PMID: 15077147, PMID: 16442236 |
| **MAPK Signaling Pathway (Cell Survival, Inflammation, Apoptosis, Osmoregulation)** | microPathway (chain consisting of 3 nodes) brings to cell servival, cell militory, inflammation, apoptosis, osmoregulation. | PMID: 15845648, PMID: 15550393, PMID: 16683917 |
| **MAPK Signaling Pathway (Gene Expression)** | microPathway (chain consisting of 3 nodes) regulates gene expression. JNK-regulated transcription factors help to regulate gene expression in response to a variety of cellular stimuli, including stress events. | PMID: 12220675, PMID: 16680093, PMID: 16185188 |
| **DDR pathway (MMR)** | Mismatch repair: 1)it recognizes a wide spectrum of mismatches, embedded in correctly base-pairing nucleotides. 2)mismatch repair discriminates between a correct nucleotide in the template strand and a incorrect nucleotide in the replicated DNA. | PMID: 11509249, PMID: 9099749, PMID: 11113111, PMID: 17919654 |
| **Mismatch Repair Main Pathway** | Mismatch repair: 1)it recognizes a wide spectrum of mismatches, embedded in correctly base-pairing nucleotides. 2)mismatch repair discriminates between a correct nucleotide in the template strand and a incorrect nucleotide in the replicated DNA. | PMID: 11509249, PMID: 9099749, PMID: 11113111, PMID: 15932942 |
| **Mitochondrial Apopotosis Main Pathway** | Apoptosis is a naturally occurring process by which a cell is directed to Programmed Cell Death. There are two pathways that lead to Apoptosis, an "Extrinsic" and an "Intrinsic" Pathway. Apoptosis in Mitochondria is intrinsic apoptosis pathway. | PMID: 15914728, PMID: 15350837 |
| **Mitochondrial Apopotosis Pathway (Apoptosis)** | microPathway (chain consisting of 3 nodes) activates apoptosis. The Caspase9 cleaves Procaspase3 and activates Caspase3, or Caspase8 cleaves Procaspase3 directly and activates it. Caspase3 then commits the cell to Apoptosis. | PMID: 15914728, PMID: 15838523 |
| **Mitochondrial Apopotosis Pathway (Depolarization)** | microPathway (chain consisting of 3 nodes) activates depolarization. | PMID: 16175394, PMID: 16148088, PMID: 15985761 |
| **Mitochondrial Apopotosis Pathway (DNA fragmentation)** | microPathway (chain consisting of 3 nodes) brings to DNA fragmentation. AIF (Apoptosis Inducing Factor) is becoming active upon translocation from Mitochondria to nuclei, where it initiates chromatin condensation and large-scale DNA fragmentation. | PMID: 16120419 |
| **Mitochondrial Apopotosis Pathway (Gene expression)** | microPathway (chain consisting of 3 nodes) activates gene expression. | PMID: 16175394, PMID: 15933766 |
| **mTOR Main Pathway** | mTOR (Mammalian Target of Rapamycin) is a 289-kDa serine/threonine protein kinase and a member of the PIKK family. mTOR functions as a central element in a signaling pathway involved in the control of cell growth and proliferation. | PMID: 16469695 |
| **mTOR Pathway (Actin organization)** | microPathway (chain consisting of 3 nodes) regulates actin organization. Rapamycin-insensitive mTORC2 controls the actin cytoskeleton and thereby determines the shape of the cell. | PMID: 15517868, PMID: 15389631, PMID: 15905878 |
| **mTOR Pathway (Akt signaling)** | microPathway (chain consisting of 3 nodes) activates Akt signaling. Signaling through the PI3K/Akt pathway is initiated by mitogenic stimuli from Growth factors that bind receptors in the cell membrane. | PMID: 16452206, PMID: 16391386, PMID: 16397245 |
| **mTOR Pathway (Scanning)** | microPathway (chain consisting of 3 nodes) activates scanning. S6K1 phosphorylates and activates the 40S ribosomal S6 protein, facilitating the recruitment of the 40S ribosomal subunit into actively translating polysomes. | PMID: 15517868, PMID: 15389631, PMID: 15905878 |
| **mTOR Pathway (Translation on)** | microPathway (chain consisting of 3 nodes) controls translation on. The best-characterized effectors downstream of mTOR are 2 signaling pathways that act in parallel to control mRNA translation. | PMID: 15517868, PMID: 15389631, PMID: 15905878 |
| **mTOR Pathway (VEGF pathway)** | microPathway (chain consisting of 3 nodes) activates VEGF pathway. mTORC1 regulates VEGF (Vascular Endothelial Growth Factor) by phosphorylating HIF1Alpha (Hypoxia-Inducible Factor-1-Alpha Subunit). | PMID: 15517868, PMID: 15389631, PMID: 15905878 |
| **NGF (Negative) Main Pathway** | A family of Neurotrophins, which includes NGF (Nerve Growth Factor), BDNF (Brain Derived Neurotrophic Factor), NTF3 (Neurotrophin3) and NTF4/5 (Neurotrophin-4/5), maintains critical balance of cell survival and death. | PMID: 11520933, PMID: 16091303 |
| **NGF (Negative) Pathway (Apoptosis)** | microPathway (chain consisting of 3 nodes) regulates apoptosis. JNKs, in turn, upregulate p53 and the proapoptotic member of the Bcl2 family: BAX in a sequencial manner, which bring about apoptosis of the neuronal cells. | PMID: 16411893, PMID: 15383672, PMID: 15921851 |
| **NGF (Positive) Main Pathway** | A family of Neurotrophins, which includes NGF (Nerve Growth Factor), BDNF (Brain Derived Neurotrophic Factor), NTF3 (Neurotrophin3) and NTF4/5 (Neurotrophin-4/5), maintains critical balance of cell survival and death. | PMID: 11520933, PMID: 16091303, PMID: 16411893 |
| **NHEJ mechanisms of DSBs repair effect** | Non homologous DNA end joining – DNA repair | PMID: 24051048 |
| **Notch Main Pathway** | Interaction between Notch and its proposed ligands initiates a signaling cascade that governs cell fate decisions such as differentiation, proliferation, and apoptosis in numerous tissue types. | PMID: 10779430, PMID: 10805736 |
| **DDR Pathway (NER)** | NER (Nucleotide Excision Repair) is the most flexible of the DNA repair pathways considering the diversity of DNA lesions it acts upon. The most significant of these lesions are pyrimidine dimers caused by the UV component of sunlight. | PMID: 11100718, PMID: 11357144 |
| **p38 (Negative) Main Signaling Pathway** | p38MAPKs belong to mammalian Stress-activated MAPK family. The p38MAPK subfamily plays important roles in in the inhibition of Cell cycle progression, in developmental processes, Cytokine production and the Stress response. | PMID: 16601071, PMID: 1610375 |
| **p38 (Positive) Main Signaling Pathway** | p38MAPKs belong to mammalian Stress-activated MAPK family. The p38MAPK subfamily plays important roles in in the inhibition of Cell cycle progression, in developmental processes, Cytokine production and the Stress response. | PMID: 16601071, PMID: 1610375 |
| **p53 Signaling (Negative) Main Pathway** | p53 is a tumour suppressor protein that regulates the expression of genes involved in Apoptosis, Growth arrest, Inhibition of cell cycle progression, Differentiation, accelerated DNA repair or Senescence in response to Genotoxic or Cellular Stress. | PMID: 16514420, PMID: 16515600, PMID: 16439820 |
| **p53 Signaling (Negative) Pathway (p53 Degradation)** | microPathway (chain consisting of 3 nodes) brings to p53 Degradation. PTEN (Phosphatase and Tensin Homolog) inhibits MDM2- mediated p53 degradation. | PMID: 16514420, PMID: 16439820 |
| **PAK Main Pathway** | PAKs are a growing family of serine/threonine protein kinases, which are activated in response to extracellular signals and regulate cell shape, motility, gene expression, cytoskeletal actin assembly, MAPK pathways, neurite outgrowth, apoptosis. | PMID: 9560242, PMID: 1112103 |
| **PAK Pathway (Actin Cytoskeleton)** | microPathway regulates actin cytoskeleton. The GTP-bound forms of CDC42 and Rac regulate assembly of the actin cytoskeleton, in part by stimulation of PAKs and in part by activation of the intermediate switch proteins, WASP and N-WASP. | PMID: 12167697 |
| **PAK Pathway (Myosin Activation)** | microPathway regulates myosin activation. One way in which PAKs can influence actin organization and cell polarity is through phosphorylation of substrates such as MLCK (Myosin Light Chain Kinase) and myosin itself. | PMID: 10092231 |
| **PPAR Main Pathway** | PPARs are ligand-inducible transcription factors that belong to the nuclear hormone receptor superfamily, together with the receptors for thyroid hormone, retinoids, steroid hormones and Vitamin-D that act as ligand-activated transcription factors. | PMID: 12193105, PMID: 11457759 |
| **PTEN Main Pathway** | PTEN is one of the most frequently mutated tumor suppressors in human cancer that functions primarily as a cytoplasmic phosphatase to regulate crucial signal transduction pathways involving growth, adhesion, migration, invasion, apoptosis. | PMID: 16453012, PMID: 15584861 |
| **RANK Signaling in Osteoclast Main Pathway** | The TNF-family molecule RANKL (Receptor Activator of NF-KappaB Ligand; also known as OPGL, TRANCE, ODF and TNFSF11) and its receptor RANK are key regulators of bone remodeling, and they are essential for the development and activation of osteoclasts. | PMID: 11861618 |
| **RANK Signaling in Osteoclast Pathway (IKBs Degradation)** | microPathway brings to IKBs degradation. Stimuli that activate NF-KappaB induce the activation of IKKs (I-KappaB Kinases), resulting in the phosphorylation and subsequent proteasome-mediated degradation of I-KappaB. | PMID: 10384146 |
| **RAS Main Pathway** | Ras displays dynamic nature of signal transduction across the membrane in determining cellular responses to external stimuli, is associated to an ever increasing list of signaling pathways with immense gene expression strategies. | PMID: 15317758, PMID: 18413774, PMID: 18299280 |
| **Telomere Main Pathway** | The nuclei contain three different RNA Pol., designated I, II and III. Like the DNA Pol. that catalyzes DNA replication, RNA Pol. catalyze the formation of the phosphodiester bonds that link the nucleotides together to form a linear chain. | PMID: 8367480, PMID: 16126390 |
| **RNA Polymerase II Complex Pathway** | RNA polymerase II complex mediates RNA transcription | PMID: 25275381 |
| **Cell Cycle Pathway (SCC during S-phase)** | Sister chromatid cohesion | PMID: 25205350 |
| **SMAD (Negative) Main Pathway** | TGF-Beta, the Activins and the BMPs signal through heteromeric complexes of Type-II and Type-I serine-threonine kinase receptors, which activate the downstream SMAD (Sma and Mad Related Family) signal transduction pathway. | PMID: 10615055, PMID: 16186117, PMID: 15948132 |
| **SMAD (Negative) Pathway (Degradation)** | microPathway brings to degradation. The SMAD7-SMURF complex after TGF-Beta stimulation and ubiquitinates the receptors on the cell surface or endosomal membranes; these are then targeted for degradation in proteasomes and lysosomes. | PMID: 11792802 |
| **SMAD (Positive) Main Pathway** | TGF-Beta, the Activins and the BMPs signal through heteromeric complexes of Type-II and Type-I serine-threonine kinase receptors, which activate the downstream SMAD (Sma and Mad Related Family) signal transduction pathway. | PMID: 10615055, PMID: 16186117, PMID: 15948132 |
| **SMAD (Positive) Pathway (Degradation)** | microPathway brings to degradation. The SMAD7-SMURF complex after TGF-Beta stimulation and ubiquitinates the receptors on the cell surface or endosomal membranes; these are then targeted for degradation in proteasomes and lysosomes. | PMID: 11792802 |
| **Cell Cycle Pathway (Metaphase-Anaphase)** | Spindle assembly and chromosome separation | PMID: 24687279 |
| **Cell Cycle Pathway (Origin of S-phase)** | Start of DNA replication in early S phase | PMID: 25264619 |
| **STAT3 Main Pathway** | STATs (Signal Transducers and Activators of Transcription) are a family of cytoplasmic proteins with SH2 domains that act as signal messengers and transcription factors and participate in normal cellular responses to Cytokines and GFs. | PMID: 15970662, PMID: 15840591 |
| **TGF beta Main Pathway** | The TGF-Beta (Transforming Growth Factor) super family, comprises a large and diverse group of polypeptide morphogens including the prototype of the family-the TGF-Betathemselves as well as the BMPs, and the GDFs. | PMID: 10801076, PMID: 12008039 |
| **TGF beta Pathway (Epithelial mesehchymal transition)** | microPathway operates epithelial mesenchymal transdifferentiation.Epigenetic loss of TGF-Beta signaling promotes tumorigenesis via suppression of the immune system and changes in cell differentiation of epithelial tumor cells (it termed EMT). | PMID: 9418859, PMID: 12008039 |
| **TGF beta Pathway (Post-transcriptional G1 arrest)** | microPathway brings to post transcriptional G1 arrest. Besides the direct induction or repression of target gene expression, TGF-Beta induces a variety of complex cellular responses, depending on the cell type, most notably growth arrest in late G1. | PMID: 11752631 |
| **TGF beta Pathway (SnON degradation)** | microPathway brings to SnON degradation. SMAD3 recruits the APC (Anaphase-Promoting Complex) and Cdh1 (Cadherin-1) to SnON, thus providing an alternative mechanism to target SnON for ubiquitination and degradation. | PMID: 11752631 |
| **TGF beta Pathway (Tumor suppression)** | microPathway brings to tumor suppression. Processes involved in tumor inhibition including maintenance of genomic stability, suppression of telomerase activity and prevention of inappropriate angiogenesis have implicated TGF-Beta. | PMID: 9418859 |
| **TGF beta Pathway (Tumorigenesis)** | microPathway operates tumorogenesis. Some of the activated target genes stimulate tumorigenesis while others suppress tumorigenesis. | PMID: 12479057 |
| **TNF (Negative) Main Pathway** | TNF (Tumor Necrosis Factor) is a multifunctional proinflammatory cytokine, with effects on lipid metabolism, coagulation, insulin resistance, and endothelial function. Members of the TNFR superfamily can send both survival and death signals to cells. | PMID: 11923844, PMID: 10102625 |
| **TNF (Negative) Pathway (Apoptosis)** | microPathway brings to apoptosis. Activated Caspase8 subsequently initiates a proteolytic cascade that includes other Caspases (Caspases3,6,7) and ultimately induces apoptosis. | PMID: 8661109 |
| **TNF (Positive) Main Pathway** | TNF (Tumor Necrosis Factor) is a multifunctional proinflammatory cytokine, with effects on lipid metabolism, coagulation, insulin resistance, and endothelial function. Members of the TNFR superfamily can send both survival and death signals to cells. | PMID: 11923844, PMID: 10102625 |
| **TNF (Positive) Pathway (Gene expression)** | microPathway (chain consisting of 3 nodes) activates gene expression. | PMID: 8661109 |
| **TNF (Positive) Pathway (IKBs degradation)** | microPathway (chain consisting of 3 nodes) brings to IKBs вegradation. | PMID: 11514191 |
| **TRAF (Negative) Main Pathway** | RANK provokes biochemical signaling via the recruitment of intracellular adaptor TRAFs after ligand binding and receptor oligomerization. Signaling through RANK is essential for the differentiation and activation of osteoclasts. | PMID: 10764746 |
| **TRAF (Negative) Pathway (IKBs Degradation)** | microPathway brings to IKBs degradation. IKK phosphorylates I-KappaB. This phosphorylation triggers ubiquitination and subsequent degradation of I-KappaB, resulting in the release of NF-KappaB subunits that translocate to the nucleus. | PMID: 11744690 |
| **TRAF (Positive) Main Pathway** | RANK provokes biochemical signaling via the recruitment of intracellular adaptor TRAFs after ligand binding and receptor oligomerization. Signaling through RANK is essential for the differentiation and activation of osteoclasts. | PMID: 10764746 |
| **TRAF (Positive) Pathway (IKBs Degradation)** | microPathway brings to IKBs degradation. IKK phosphorylates I-KappaB. This phosphorylation triggers ubiquitination and subsequent degradation of I-KappaB, resulting in the release of NF-KappaB subunits that translocate to the nucleus. | PMID: 11744690 |
| **Transcription of mRNA Pathway** | Transcription is the process through which a DNA sequence is enzymatically copied by an RNA polymerase to produce a complementary RNA. Transcription can also be defined as a process that transcribes genetic information from DNA into RNA. | PMID: 17008216, PMID: 16707260 |
| **Cell Cycle Pathway (End of S-phase)** | Transition and termination of DNA replication | PMID: 24894899 |
| **Translation Regulation of EIF4F activity** | eIF4 (Eukaryotic Initiation Factor-4) and p70S6K play critical roles in translational regulation. Recruitment of mRNAs to ribosomes to initiate translation is mediated by initiation factors of the eIF4 group and the PABP (Poly (A)-Binding Protein). | PMID: 12814655 |
| **Ubiquitin Proteasome Main Pathway** | Two systems that play important roles in proteolysis in cytosol are the Calpain Proteases and the Ub-Proteasome complex, which consists of the Ub-conjugating system and the Proteasome , functions widely in intracellular protein turnover. | PMID: 15688069, PMID: 16113643, PMID: 15230346 |
| **Ubiquitin Proteasome Pathway (Degraded Protein)** | Ub undergoes an ATP-dependent reaction with proteins, which condenses its C-terminal Glycine residues with Lysine amino groups on the target protein. Such modified proteins are degraded soon afterward by the Proteasome. | PMID: 11917093, PMID: 10797484 |
| **VEGF Main Pathway** | VEGF signaling plays a role in promoting the proliferation and differentiation of the endothelial lineage from the earliest stages of development, whereas the TIE2 pathway acts to promote the recruitment of supporting cells and vessel stabilization. | PMID: 10995484, PMID: 16403941, PMID: 16311850 |
| **VEGF Pathway (Actin Reorganization)** | microPathway (chain consisting of 3 nodes) operates actin reorganization. The Actin-anchoring proteins such as Talin and Vinculin to the focal adhesion plaque, are essential for VEGFA-induced actin reorganization. | PMID: 11741095 |
| **VEGF Pathway (Nitric Oxide Production)** | microPathway brings to nitric oxide prodaction. VEGF-A-induced Ca2+ mobilization is involved in short-term production of Nitric Oxide and Ptg (Prostaglandin). | PMID: 11741095 |
| **Wnt Main Pathway** | The WNT genes encode a large family of secreted protein growth factors. During development, WNTs have diverse roles in governing cell fate, proliferation, migration, polarity, and death. | PMID: 16267791, PMID: 15754042 |
| **Wnt Pathway (Ctnn-b Degradation)** | microPathway brings to Ctnn-b degradation.The increased expression of Beta-TRCP and the Ubiquitin Conjugating enzyme UbC4/5E2, both involved in degradation of Ctnn-Beta, could act as a negative feedback loop in WNT signaling. | PMID: 16520547, PMID: 12050670 |

Metabolic pathways

| Pathway Name | URL |
| --- | --- |
| phenylethylamine degradation I | <http://humancyc.org/HUMAN/new-image?object=2PHENDEG-PWY> |
| methionine salvage | <http://humancyc.org/HUMAN/new-image?object=ADENOSYLHOMOCYSCAT-PWY> |
| alanine biosynthesis/degradation | <http://humancyc.org/HUMAN/new-image?object=ALANINE-DEG3-PWY> |
| ornithine de novo biosynthesis | <http://humancyc.org/HUMAN/new-image?object=ARGININE-SYN4-PWY> |
| spermine biosynthesis | <http://humancyc.org/HUMAN/new-image?object=ARGSPECAT-PWY> |
| asparagine biosynthesis | <http://humancyc.org/HUMAN/new-image?object=ASPARAGINE-BIOSYNTHESIS> |
| asparagine degradation | <http://humancyc.org/HUMAN/new-image?object=ASPARAGINE-DEG1-PWY> |
| aspartate biosynthesis | <http://humancyc.org/HUMAN/new-image?object=ASPARTATESYN-PWY> |
| beta-alanine degradation | <http://humancyc.org/HUMAN/new-image?object=BETA-ALA-DEGRADATION-I-PWY> |
| lactose degradation III | <http://humancyc.org/HUMAN/new-image?object=BGALACT-PWY> |
| spermidine biosynthesis | <http://humancyc.org/HUMAN/new-image?object=BSUBPOLYAMSYN-PWY> |
| choline degradation | <http://humancyc.org/HUMAN/new-image?object=CHOLINE-BETAINE-ANA-PWY> |
| coenzyme A biosynthesis | <http://humancyc.org/HUMAN/new-image?object=COA-PWY-1> |
| L-cysteine degradation I | <http://humancyc.org/HUMAN/new-image?object=CYSTEINE-DEG-PWY> |
| superoxide radicals degradation | <http://humancyc.org/HUMAN/new-image?object=DETOX1-PWY> |
| fatty acid beta-oxidation | <http://humancyc.org/HUMAN/new-image?object=FAO-PWY> |
| fatty acid elongation -- saturated | <http://humancyc.org/HUMAN/new-image?object=FASYN-ELONG-PWY> |
| glutamine biosynthesis | <http://humancyc.org/HUMAN/new-image?object=GLNSYN-PWY> |
| N-acetylglucosamine degradation I | <http://humancyc.org/HUMAN/new-image?object=GLUAMCAT-PWY> |
| GABA shunt | <http://humancyc.org/HUMAN/new-image?object=GLUDEG-I-PWY> |
| glutamate biosynthesis/degradation | <http://humancyc.org/HUMAN/new-image?object=GLUTAMATE-SYN2-PWY> |
| glutamine degradation/glutamate biosynthesis | <http://humancyc.org/HUMAN/new-image?object=GLUTAMINDEG-PWY> |
| glutathione biosynthesis | <http://humancyc.org/HUMAN/new-image?object=GLUTATHIONESYN-PWY> |
| glutathione redox reactions II | <http://humancyc.org/HUMAN/new-image?object=GLUT-REDOX-PWY> |
| creatine biosynthesis | <http://humancyc.org/HUMAN/new-image?object=GLYCGREAT-PWY> |
| glycine cleavage | <http://humancyc.org/HUMAN/new-image?object=GLYCLEAV-PWY> |
| glycine biosynthesis | <http://humancyc.org/HUMAN/new-image?object=GLYSYN-ALA-PWY> |
| glycine/serine biosynthesis | <http://humancyc.org/HUMAN/new-image?object=GLYSYN-PWY> |
| heme biosynthesis from uroporphyrinogen-III I | <http://humancyc.org/HUMAN/new-image?object=HEME-BIOSYNTHESIS-II> |
| cysteine biosynthesis/homocysteine degradation (trans-sulfuration) | <http://humancyc.org/HUMAN/new-image?object=HOMOCYSDEGR-PWY> |
| 4-hydroxyproline degradation | <http://humancyc.org/HUMAN/new-image?object=HYDROXYPRODEG-PWY> |
| isoleucine degradation | <http://humancyc.org/HUMAN/new-image?object=ILEUDEG-PWY> |
| leucine degradation | <http://humancyc.org/HUMAN/new-image?object=LEU-DEG2-PWY> |
| triacylglycerol degradation | <http://humancyc.org/HUMAN/new-image?object=LIPAS-PWY> |
| phospholipases | <http://humancyc.org/HUMAN/new-image?object=LIPASYN-PWY> |
| lysine degradation I (saccharopine pathway) | <http://humancyc.org/HUMAN/new-image?object=LYSINE-DEG1-PWY> |
| malate-aspartate shuttle | <http://humancyc.org/HUMAN/new-image?object=MALATE-ASPARTATE-SHUTTLE-PWY> |
| D-mannose degradation | <http://humancyc.org/HUMAN/new-image?object=MANNCAT-PWY> |
| dolichyl-diphosphooligosaccharide biosynthesis | <http://humancyc.org/HUMAN/new-image?object=MANNOSYL-CHITO-DOLICHOL-BIOSYNTHESIS> |
| methionine degradation | <http://humancyc.org/HUMAN/new-image?object=METHIONINE-DEG1-PWY> |
| methylglyoxal degradation VI | <http://humancyc.org/HUMAN/new-image?object=MGLDLCTANA-PWY> |
| NAD salvage | <http://humancyc.org/HUMAN/new-image?object=NAD-BIOSYNTHESIS-III> |
| NAD phosphorylation and dephosphorylation | <http://humancyc.org/HUMAN/new-image?object=NADPHOS-DEPHOS-PWY-1> |
| NAD de novo biosynthesis | <http://humancyc.org/HUMAN/new-image?object=NADSYN-PWY> |
| pentose phosphate pathway (non-oxidative branch) | <http://humancyc.org/HUMAN/new-image?object=NONOXIPENT-PWY> |
| pentose phosphate pathway (oxidative branch) | <http://humancyc.org/HUMAN/new-image?object=OXIDATIVEPENT-PWY-1> |
| adenine and adenosine salvage I | <http://humancyc.org/HUMAN/new-image?object=P121-PWY> |
| pentose phosphate pathway | <http://humancyc.org/HUMAN/new-image?object=PENTOSE-P-PWY> |
| phenylalanine degradation/tyrosine biosynthesis | <http://humancyc.org/HUMAN/new-image?object=PHENYLALANINE-DEG1-PWY> |
| pyridoxal 5'-phosphate salvage | <http://humancyc.org/HUMAN/new-image?object=PLPSAL-PWY> |
| propionyl-CoA degradation | <http://humancyc.org/HUMAN/new-image?object=PROPIONMET-PWY> |
| proline biosynthesis | <http://humancyc.org/HUMAN/new-image?object=PROSYN-PWY> |
| proline degradation | <http://humancyc.org/HUMAN/new-image?object=PROUT-PWY> |
| trehalose degradation | <http://humancyc.org/HUMAN/new-image?object=PWY0-1182> |
| biotin-carboxyl carrier protein assembly | <http://humancyc.org/HUMAN/new-image?object=PWY0-1264> |
| lipoate biosynthesis and incorporation | <http://humancyc.org/HUMAN/new-image?object=PWY0-1275> |
| pyrimidine ribonucleosides degradation | <http://humancyc.org/HUMAN/new-image?object=PWY0-1295> |
| purine ribonucleosides degradation to ribose-1-phosphate | <http://humancyc.org/HUMAN/new-image?object=PWY0-1296> |
| glutamate dependent acid resistance | <http://humancyc.org/HUMAN/new-image?object=PWY0-1305> |
| acetate conversion to acetyl-CoA | <http://humancyc.org/HUMAN/new-image?object=PWY0-1313> |
| superpathway of pyrimidine ribonucleotides de novo biosynthesis | <http://humancyc.org/HUMAN/new-image?object=PWY0-162> |
| lipoate salvage | <http://humancyc.org/HUMAN/new-image?object=PWY0-522> |
| PRPP biosynthesis | <http://humancyc.org/HUMAN/new-image?object=PWY0-662> |
| putrescine degradation III | <http://humancyc.org/HUMAN/new-image?object=PWY-0> |
| formaldehyde oxidation | <http://humancyc.org/HUMAN/new-image?object=PWY-1801> |
| glutamate removal from folates | <http://humancyc.org/HUMAN/new-image?object=PWY-2161B> |
| folate polyglutamylation | <http://humancyc.org/HUMAN/new-image?object=PWY-2161> |
| folate transformations | <http://humancyc.org/HUMAN/new-image?object=PWY-2201> |
| myo-inositol de novo biosynthesis | <http://humancyc.org/HUMAN/new-image?object=PWY-2301> |
| glycine betaine degradation | <http://humancyc.org/HUMAN/new-image?object=PWY-3661-1> |
| uracil degradation | <http://humancyc.org/HUMAN/new-image?object=PWY-3982> |
| sphingomyelin metabolism/ceramide salvage | <http://humancyc.org/HUMAN/new-image?object=PWY3DJ-11281> |
| sphingosine and sphingosine-1-phosphate metabolism | <http://humancyc.org/HUMAN/new-image?object=PWY3DJ-11470> |
| ceramide de novo biosynthesis | <http://humancyc.org/HUMAN/new-image?object=PWY3DJ-12> |
| phosphatidylcholine biosynthesis | <http://humancyc.org/HUMAN/new-image?object=PWY3O-450> |
| gamma-glutamyl cycle | <http://humancyc.org/HUMAN/new-image?object=PWY-4041> |
| glutathione-mediated detoxification | <http://humancyc.org/HUMAN/new-image?object=PWY-4061> |
| glutathione redox reactions I | <http://humancyc.org/HUMAN/new-image?object=PWY-4081> |
| putrescine biosynthesis II | <http://humancyc.org/HUMAN/new-image?object=PWY-40> |
| sorbitol degradation I | <http://humancyc.org/HUMAN/new-image?object=PWY-4101> |
| arsenate detoxification I (glutaredoxin) | <http://humancyc.org/HUMAN/new-image?object=PWY-4202> |
| glycerol degradation | <http://humancyc.org/HUMAN/new-image?object=PWY-4261> |
| putrescine biosynthesis I | <http://humancyc.org/HUMAN/new-image?object=PWY-46> |
| UDP-D-xylose and UDP-D-glucuronate biosynthesis | <http://humancyc.org/HUMAN/new-image?object=PWY-4821> |
| protein citrullination | <http://humancyc.org/HUMAN/new-image?object=PWY-4921> |
| citrulline-nitric oxide cycle | <http://humancyc.org/HUMAN/new-image?object=PWY-4983> |
| urea cycle | <http://humancyc.org/HUMAN/new-image?object=PWY-4984> |
| phosphatidylethanolamine biosynthesis II | <http://humancyc.org/HUMAN/new-image?object=PWY4FS-6> |
| histidine degradation | <http://humancyc.org/HUMAN/new-image?object=PWY-5030> |
| 2-oxoisovalerate decarboxylation to isobutanoyl-CoA | <http://humancyc.org/HUMAN/new-image?object=PWY-5046> |
| glycogen biosynthesis | <http://humancyc.org/HUMAN/new-image?object=PWY-5067> |
| 2-oxoglutarate decarboxylation to succinyl-CoA | <http://humancyc.org/HUMAN/new-image?object=PWY-5084> |
| geranylgeranyldiphosphate biosynthesis | <http://humancyc.org/HUMAN/new-image?object=PWY-5120> |
| trans, trans-farnesyl diphosphate biosynthesis | <http://humancyc.org/HUMAN/new-image?object=PWY-5123> |
| 2-oxobutanoate degradation | <http://humancyc.org/HUMAN/new-image?object=PWY-5130> |
| fatty acid beta-oxidation (unsaturated, odd number) | <http://humancyc.org/HUMAN/new-image?object=PWY-5137> |
| fatty acid activation | <http://humancyc.org/HUMAN/new-image?object=PWY-5143> |
| acyl-CoA hydrolysis | <http://humancyc.org/HUMAN/new-image?object=PWY-5148> |
| acetyl-CoA biosynthesis from citrate | <http://humancyc.org/HUMAN/new-image?object=PWY-5172> |
| glutaryl-CoA degradation | <http://humancyc.org/HUMAN/new-image?object=PWY-5177> |
| tetrapyrrole biosynthesis | <http://humancyc.org/HUMAN/new-image?object=PWY-5189> |
| cardiolipin biosynthesis | <http://humancyc.org/HUMAN/new-image?object=PWY-5269> |
| morphine biosynthesis | <http://humancyc.org/HUMAN/new-image?object=PWY-5270> |
| sulfite oxidation | <http://humancyc.org/HUMAN/new-image?object=PWY-5326> |
| superpathway of methionine degradation | <http://humancyc.org/HUMAN/new-image?object=PWY-5328> |
| L-cysteine degradation II | <http://humancyc.org/HUMAN/new-image?object=PWY-5329> |
| taurine biosynthesis | <http://humancyc.org/HUMAN/new-image?object=PWY-5331> |
| sulfate activation for sulfonation | <http://humancyc.org/HUMAN/new-image?object=PWY-5340> |
| thiosulfate disproportionation III (rhodanese) | <http://humancyc.org/HUMAN/new-image?object=PWY-5350> |
| methylglyoxal degradation I | <http://humancyc.org/HUMAN/new-image?object=PWY-5386> |
| methylthiopropionate biosynthesis | <http://humancyc.org/HUMAN/new-image?object=PWY-5389> |
| acetone degradation I (to methylglyoxal) | <http://humancyc.org/HUMAN/new-image?object=PWY-5451> |
| methylglyoxal degradation III | <http://humancyc.org/HUMAN/new-image?object=PWY-5453> |
| lactate fermentation (reoxidation of cytosolic NADH) | <http://humancyc.org/HUMAN/new-image?object=PWY-5481> |
| UDP-N-acetyl-D-galactosamine biosynthesis I | <http://humancyc.org/HUMAN/new-image?object=PWY-5512> |
| UDP-N-acetyl-D-galactosamine biosynthesis II | <http://humancyc.org/HUMAN/new-image?object=PWY-5514> |
| D-glucuronate degradation | <http://humancyc.org/HUMAN/new-image?object=PWY-5525> |
| tryptophan degradation to 2-amino-3-carboxymuconate semialdehyde | <http://humancyc.org/HUMAN/new-image?object=PWY-5651> |
| 2-amino-3-carboxymuconate semialdehyde degradation to glutaryl-CoA | <http://humancyc.org/HUMAN/new-image?object=PWY-5652> |
| NAD biosynthesis from 2-amino-3-carboxymuconate semialdehyde | <http://humancyc.org/HUMAN/new-image?object=PWY-5653> |
| GDP-mannose biosynthesis | <http://humancyc.org/HUMAN/new-image?object=PWY-5659> |
| GDP-glucose biosynthesis II | <http://humancyc.org/HUMAN/new-image?object=PWY-5661-1> |
| tetrahydrobiopterin de novo biosynthesis | <http://humancyc.org/HUMAN/new-image?object=PWY-5663> |
| CDP-diacylglycerol biosynthesis | <http://humancyc.org/HUMAN/new-image?object=PWY-5667> |
| epoxysqualene biosynthesis | <http://humancyc.org/HUMAN/new-image?object=PWY-5670> |
| UMP biosynthesis | <http://humancyc.org/HUMAN/new-image?object=PWY-5686> |
| urate biosynthesis/inosine 5'-phosphate degradation | <http://humancyc.org/HUMAN/new-image?object=PWY-5695> |
| 4-hydroxybenzoate biosynthesis | <http://humancyc.org/HUMAN/new-image?object=PWY-5754> |
| ubiquinol-10 biosynthesis | <http://humancyc.org/HUMAN/new-image?object=PWY-5872> |
| heme degradation | <http://humancyc.org/HUMAN/new-image?object=PWY-5874> |
| hypusine biosynthesis | <http://humancyc.org/HUMAN/new-image?object=PWY-5905> |
| superpathway of geranylgeranyldiphosphate biosynthesis I (via mevalonate) | <http://humancyc.org/HUMAN/new-image?object=PWY-5910> |
| heme biosynthesis | <http://humancyc.org/HUMAN/new-image?object=PWY-5920> |
| L-glutamine tRNA biosynthesis | <http://humancyc.org/HUMAN/new-image?object=PWY-5921> |
| glycogenolysis | <http://humancyc.org/HUMAN/new-image?object=PWY-5941-1> |
| thio-molybdenum cofactor biosynthesis | <http://humancyc.org/HUMAN/new-image?object=PWY-5963> |
| fatty acid biosynthesis initiation | <http://humancyc.org/HUMAN/new-image?object=PWY-5966-1> |
| stearate biosynthesis | <http://humancyc.org/HUMAN/new-image?object=PWY-5972> |
| palmitate biosynthesis | <http://humancyc.org/HUMAN/new-image?object=PWY-5994> |
| oleate biosynthesis | <http://humancyc.org/HUMAN/new-image?object=PWY-5996> |
| gamma-linolenate biosynthesis | <http://humancyc.org/HUMAN/new-image?object=PWY-6000> |
| acyl carrier protein metabolism | <http://humancyc.org/HUMAN/new-image?object=PWY-6012-1> |
| serotonin and melatonin biosynthesis | <http://humancyc.org/HUMAN/new-image?object=PWY-6030> |
| bile acid biosynthesis, neutral pathway | <http://humancyc.org/HUMAN/new-image?object=PWY-6061> |
| zymosterol biosynthesis | <http://humancyc.org/HUMAN/new-image?object=PWY-6074> |
| 1,25-dihydroxyvitamin D3 biosynthesis | <http://humancyc.org/HUMAN/new-image?object=PWY-6076> |
| L-carnitine biosynthesis | <http://humancyc.org/HUMAN/new-image?object=PWY-6100> |
| mitochondrial L-carnitine shuttle | <http://humancyc.org/HUMAN/new-image?object=PWY-6111> |
| spermine and spermidine degradation I | <http://humancyc.org/HUMAN/new-image?object=PWY-6117> |
| glycerol-3-phosphate shuttle | <http://humancyc.org/HUMAN/new-image?object=PWY-6118> |
| 5-aminoimidazole ribonucleotide biosynthesis | <http://humancyc.org/HUMAN/new-image?object=PWY-6121> |
| inosine-5'-phosphate biosynthesis | <http://humancyc.org/HUMAN/new-image?object=PWY-6124> |
| dolichol and dolichyl phosphate biosynthesis | <http://humancyc.org/HUMAN/new-image?object=PWY-6129> |
| lanosterol biosynthesis | <http://humancyc.org/HUMAN/new-image?object=PWY-6132> |
| (S)-reticuline biosynthesis | <http://humancyc.org/HUMAN/new-image?object=PWY-6133> |
| CMP-N-acetylneuraminate biosynthesis I (eukaryotes) | <http://humancyc.org/HUMAN/new-image?object=PWY-6138> |
| creatine-phosphate biosynthesis | <http://humancyc.org/HUMAN/new-image?object=PWY-6158> |
| histamine biosynthesis | <http://humancyc.org/HUMAN/new-image?object=PWY-6173> |
| histamine degradation | <http://humancyc.org/HUMAN/new-image?object=PWY-6181> |
| thyroid hormone biosynthesis | <http://humancyc.org/HUMAN/new-image?object=PWY-6241> |
| thyroid hormone metabolism I (via deiodination) | <http://humancyc.org/HUMAN/new-image?object=PWY-6260> |
| thyroid hormone metabolism II (via conjugation and/or degradation) | <http://humancyc.org/HUMAN/new-image?object=PWY-6261> |
| selenocysteine biosynthesis | <http://humancyc.org/HUMAN/new-image?object=PWY-6281> |
| cysteine biosynthesis | <http://humancyc.org/HUMAN/new-image?object=PWY-6292> |
| tryptophan degradation X (mammalian, via tryptamine) | <http://humancyc.org/HUMAN/new-image?object=PWY-6307> |
| L-kynurenine degradation | <http://humancyc.org/HUMAN/new-image?object=PWY-6309> |
| serotonin degradation | <http://humancyc.org/HUMAN/new-image?object=PWY-6313> |
| L-dopa degradation | <http://humancyc.org/HUMAN/new-image?object=PWY-6334> |
| noradrenaline and adrenaline degradation | <http://humancyc.org/HUMAN/new-image?object=PWY-6342> |
| D-myo-inositol (1,4,5)-trisphosphate biosynthesis | <http://humancyc.org/HUMAN/new-image?object=PWY-6351> |
| 3-phosphoinositide biosynthesis | <http://humancyc.org/HUMAN/new-image?object=PWY-6352> |
| purine nucleotides degradation | <http://humancyc.org/HUMAN/new-image?object=PWY-6353> |
| superpathway of D-myo-inositol (1,4,5)-trisphosphate metabolism | <http://humancyc.org/HUMAN/new-image?object=PWY-6358> |
| 1D-myo-inositol hexakisphosphate biosynthesis II (mammalian) | <http://humancyc.org/HUMAN/new-image?object=PWY-6362> |
| D-myo-inositol (1,4,5)-trisphosphate degradation | <http://humancyc.org/HUMAN/new-image?object=PWY-6363> |
| D-myo-inositol (1,3,4)-trisphosphate biosynthesis | <http://humancyc.org/HUMAN/new-image?object=PWY-6364> |
| D-myo-inositol (3,4,5,6)-tetrakisphosphate biosynthesis | <http://humancyc.org/HUMAN/new-image?object=PWY-6365> |
| D-myo-inositol (1,4,5,6)-tetrakisphosphate biosynthesis | <http://humancyc.org/HUMAN/new-image?object=PWY-6366> |
| D-myo-inositol-5-phosphate metabolism | <http://humancyc.org/HUMAN/new-image?object=PWY-6367> |
| 3-phosphoinositide degradation | <http://humancyc.org/HUMAN/new-image?object=PWY-6368> |
| inositol pyrophosphates biosynthesis | <http://humancyc.org/HUMAN/new-image?object=PWY-6369> |
| ascorbate recycling (cytosolic) | <http://humancyc.org/HUMAN/new-image?object=PWY-6370> |
| superpathway of inositol phosphate compounds | <http://humancyc.org/HUMAN/new-image?object=PWY-6371> |
| alpha-tocopherol degradation | <http://humancyc.org/HUMAN/new-image?object=PWY-6377> |
| melatonin degradation I | <http://humancyc.org/HUMAN/new-image?object=PWY-6398> |
| melatonin degradation II | <http://humancyc.org/HUMAN/new-image?object=PWY-6399> |
| melatonin degradation III | <http://humancyc.org/HUMAN/new-image?object=PWY-6400> |
| superpathway of melatonin degradation | <http://humancyc.org/HUMAN/new-image?object=PWY-6402> |
| Rapoport-Luebering glycolytic shunt | <http://humancyc.org/HUMAN/new-image?object=PWY-6405> |
| thymine degradation | <http://humancyc.org/HUMAN/new-image?object=PWY-6430> |
| L-dopachrome biosynthesis | <http://humancyc.org/HUMAN/new-image?object=PWY-6481> |
| diphthamide biosynthesis | <http://humancyc.org/HUMAN/new-image?object=PWY-6482> |
| eumelanin biosynthesis | <http://humancyc.org/HUMAN/new-image?object=PWY-6498> |
| oxidized GTP and dGTP detoxification | <http://humancyc.org/HUMAN/new-image?object=PWY-6502> |
| N-acetylglucosamine degradation II | <http://humancyc.org/HUMAN/new-image?object=PWY-6517> |
| 4-aminobutyrate degradation | <http://humancyc.org/HUMAN/new-image?object=PWY-6535> |
| 1D-myo-inositol hexakisphosphate biosynthesis V (from Ins(1,3,4)P3) | <http://humancyc.org/HUMAN/new-image?object=PWY-6554> |
| glycoaminoglycan-protein linkage region biosynthesis | <http://humancyc.org/HUMAN/new-image?object=PWY-6557> |
| heparan sulfate biosynthesis (late stages) | <http://humancyc.org/HUMAN/new-image?object=PWY-6558> |
| heparan sulfate biosynthesis | <http://humancyc.org/HUMAN/new-image?object=PWY-6564> |
| chondroitin and dermatan biosynthesis | <http://humancyc.org/HUMAN/new-image?object=PWY-6566> |
| chondroitin sulfate biosynthesis (late stages) | <http://humancyc.org/HUMAN/new-image?object=PWY-6567> |
| dermatan sulfate biosynthesis (late stages) | <http://humancyc.org/HUMAN/new-image?object=PWY-6568> |
| chondroitin sulfate biosynthesis | <http://humancyc.org/HUMAN/new-image?object=PWY-6569> |
| dermatan sulfate biosynthesis | <http://humancyc.org/HUMAN/new-image?object=PWY-6571> |
| chondroitin sulfate degradation (metazoa) | <http://humancyc.org/HUMAN/new-image?object=PWY-6573> |
| dermatan sulfate degradation (metazoa) | <http://humancyc.org/HUMAN/new-image?object=PWY-6576> |
| guanosine nucleotides degradation | <http://humancyc.org/HUMAN/new-image?object=PWY-6608> |
| adenine and adenosine salvage III | <http://humancyc.org/HUMAN/new-image?object=PWY-6609> |
| tetrahydrofolate salvage from 5,10-methenyltetrahydrofolate | <http://humancyc.org/HUMAN/new-image?object=PWY-6613> |
| oxidative ethanol degradation III | <http://humancyc.org/HUMAN/new-image?object=PWY66-161> |
| ethanol degradation IV | <http://humancyc.org/HUMAN/new-image?object=PWY66-162> |
| adenine and adenosine salvage II | <http://humancyc.org/HUMAN/new-image?object=PWY-6619> |
| nicotine degradation IV | <http://humancyc.org/HUMAN/new-image?object=PWY66-201> |
| guanine and guanosine salvage | <http://humancyc.org/HUMAN/new-image?object=PWY-6620> |
| ethanol degradation II | <http://humancyc.org/HUMAN/new-image?object=PWY66-21> |
| nicotine degradation III | <http://humancyc.org/HUMAN/new-image?object=PWY66-221> |
| bupropion degradation | <http://humancyc.org/HUMAN/new-image?object=PWY66-241> |
| catecholamine biosynthesis | <http://humancyc.org/HUMAN/new-image?object=PWY66-301> |
| cholesterol biosynthesis I | <http://humancyc.org/HUMAN/new-image?object=PWY66-341> |
| flavin biosynthesis | <http://humancyc.org/HUMAN/new-image?object=PWY66-366> |
| ketogenesis | <http://humancyc.org/HUMAN/new-image?object=PWY66-367> |
| ketolysis | <http://humancyc.org/HUMAN/new-image?object=PWY66-368> |
| sucrose degradation | <http://humancyc.org/HUMAN/new-image?object=PWY66-373> |
| C20 prostanoid biosynthesis | <http://humancyc.org/HUMAN/new-image?object=PWY66-374> |
| leukotriene biosynthesis | <http://humancyc.org/HUMAN/new-image?object=PWY66-375> |
| pregnenolone biosynthesis | <http://humancyc.org/HUMAN/new-image?object=PWY66-377> |
| androgen biosynthesis | <http://humancyc.org/HUMAN/new-image?object=PWY66-378> |
| estradiol biosynthesis I | <http://humancyc.org/HUMAN/new-image?object=PWY66-380> |
| glucocorticoid biosynthesis | <http://humancyc.org/HUMAN/new-image?object=PWY66-381> |
| mineralocorticoid biosynthesis | <http://humancyc.org/HUMAN/new-image?object=PWY66-382> |
| dTMP de novo biosynthesis (mitochondrial) | <http://humancyc.org/HUMAN/new-image?object=PWY66-385> |
| fatty acid alpha-oxidation | <http://humancyc.org/HUMAN/new-image?object=PWY66-387> |
| fatty acid alpha-oxidation III | <http://humancyc.org/HUMAN/new-image?object=PWY66-388> |
| phytol degradation | <http://humancyc.org/HUMAN/new-image?object=PWY66-389> |
| fatty acid beta-oxidation (peroxisome) | <http://humancyc.org/HUMAN/new-image?object=PWY66-391> |
| lipoxin biosynthesis | <http://humancyc.org/HUMAN/new-image?object=PWY66-392> |
| aspirin-triggered lipoxin biosynthesis | <http://humancyc.org/HUMAN/new-image?object=PWY66-393> |
| aspirin triggered resolvin E biosynthesis | <http://humancyc.org/HUMAN/new-image?object=PWY66-394> |
| aspirin triggered resolvin D biosynthesis | <http://humancyc.org/HUMAN/new-image?object=PWY66-395> |
| resolvin D biosynthesis | <http://humancyc.org/HUMAN/new-image?object=PWY66-397> |
| TCA cycle | <http://humancyc.org/HUMAN/new-image?object=PWY66-398> |
| gluconeogenesis | <http://humancyc.org/HUMAN/new-image?object=PWY66-399> |
| cholesterol biosynthesis II (via 24,25-dihydrolanosterol) | <http://humancyc.org/HUMAN/new-image?object=PWY66-3> |
| glycolysis | <http://humancyc.org/HUMAN/new-image?object=PWY66-400> |
| superpathway of tryptophan utilization | <http://humancyc.org/HUMAN/new-image?object=PWY66-401> |
| superpathway of conversion of glucose to acetyl CoA and entry into the TCA cycle | <http://humancyc.org/HUMAN/new-image?object=PWY66-407> |
| superpathway of purine nucleotide salvage | <http://humancyc.org/HUMAN/new-image?object=PWY66-409> |
| superpathway of choline degradation to L-serine | <http://humancyc.org/HUMAN/new-image?object=PWY66-414> |
| carnosine biosynthesis | <http://humancyc.org/HUMAN/new-image?object=PWY66-420> |
| homocarnosine biosynthesis | <http://humancyc.org/HUMAN/new-image?object=PWY66-421> |
| D-galactose degradation V (Leloir pathway) | <http://humancyc.org/HUMAN/new-image?object=PWY66-422> |
| fructose 2,6-bisphosphate synthesis/dephosphorylation | <http://humancyc.org/HUMAN/new-image?object=PWY66-423> |
| lysine degradation II (pipecolate pathway) | <http://humancyc.org/HUMAN/new-image?object=PWY66-425> |
| hydrogen sulfide biosynthesis (trans-sulfuration) | <http://humancyc.org/HUMAN/new-image?object=PWY66-426> |
| threonine degradation | <http://humancyc.org/HUMAN/new-image?object=PWY66-428> |
| cholesterol biosynthesis III (via desmosterol) | <http://humancyc.org/HUMAN/new-image?object=PWY66-4> |
| superpathway of cholesterol biosynthesis | <http://humancyc.org/HUMAN/new-image?object=PWY66-5> |
| anandamide degradation | <http://humancyc.org/HUMAN/new-image?object=PWY6666-1> |
| dopamine degradation | <http://humancyc.org/HUMAN/new-image?object=PWY6666-2> |
| thyronamine and iodothyronamine metabolism | <http://humancyc.org/HUMAN/new-image?object=PWY-6688> |
| tRNA splicing | <http://humancyc.org/HUMAN/new-image?object=PWY-6689> |
| GDP-L-fucose biosynthesis I (from GDP-D-mannose) | <http://humancyc.org/HUMAN/new-image?object=PWY-66> |
| S-methyl-5-thio-alpha-D-ribose 1-phosphate degradation | <http://humancyc.org/HUMAN/new-image?object=PWY-6755> |
| S-methyl-5'-thioadenosine degradation | <http://humancyc.org/HUMAN/new-image?object=PWY-6756> |
| molybdenum cofactor biosynthesis | <http://humancyc.org/HUMAN/new-image?object=PWY-6823> |
| retinol biosynthesis | <http://humancyc.org/HUMAN/new-image?object=PWY-6857> |
| the visual cycle I (vertebrates) | <http://humancyc.org/HUMAN/new-image?object=PWY-6861> |
| retinoate biosynthesis I | <http://humancyc.org/HUMAN/new-image?object=PWY-6872> |
| retinoate biosynthesis II | <http://humancyc.org/HUMAN/new-image?object=PWY-6875> |
| thiamin salvage III | <http://humancyc.org/HUMAN/new-image?object=PWY-6898> |
| NADH repair | <http://humancyc.org/HUMAN/new-image?object=PWY-6938> |
| GDP-L-fucose biosynthesis II (from L-fucose) | <http://humancyc.org/HUMAN/new-image?object=PWY-6> |
| eicosapentaenoate biosynthesis | <http://humancyc.org/HUMAN/new-image?object=PWY-7049> |
| 4-hydroxy-2-nonenal detoxification | <http://humancyc.org/HUMAN/new-image?object=PWY-7112> |
| UTP and CTP de novo biosynthesis | <http://humancyc.org/HUMAN/new-image?object=PWY-7176> |
| UTP and CTP dephosphorylation II | <http://humancyc.org/HUMAN/new-image?object=PWY-7177> |
| purine deoxyribonucleosides degradation | <http://humancyc.org/HUMAN/new-image?object=PWY-7179-1> |
| 2'-deoxy-alpha-D-ribose 1-phosphate degradation | <http://humancyc.org/HUMAN/new-image?object=PWY-7180> |
| pyrimidine deoxyribonucleosides degradation | <http://humancyc.org/HUMAN/new-image?object=PWY-7181> |
| pyrimidine deoxyribonucleotides de novo biosynthesis | <http://humancyc.org/HUMAN/new-image?object=PWY-7184> |
| UTP and CTP dephosphorylation I | <http://humancyc.org/HUMAN/new-image?object=PWY-7185> |
| pyrimidine ribonucleosides salvage I | <http://humancyc.org/HUMAN/new-image?object=PWY-7193> |
| pyrimidine deoxyribonucleotide phosphorylation | <http://humancyc.org/HUMAN/new-image?object=PWY-7197> |
| pyrimidine deoxyribonucleosides salvage | <http://humancyc.org/HUMAN/new-image?object=PWY-7199> |
| superpathway of pyrimidine deoxyribonucleoside salvage | <http://humancyc.org/HUMAN/new-image?object=PWY-7200> |
| CMP phosphorylation | <http://humancyc.org/HUMAN/new-image?object=PWY-7205> |
| pyrimidine ribonucleosides degradation | <http://humancyc.org/HUMAN/new-image?object=PWY-7209> |
| pyrimidine deoxyribonucleotides biosynthesis from CTP | <http://humancyc.org/HUMAN/new-image?object=PWY-7210> |
| superpathway of pyrimidine deoxyribonucleotides de novo biosynthesis | <http://humancyc.org/HUMAN/new-image?object=PWY-7211> |
| adenosine ribonucleotides de novo biosynthesis | <http://humancyc.org/HUMAN/new-image?object=PWY-7219> |
| guanosine ribonucleotides de novo biosynthesis | <http://humancyc.org/HUMAN/new-image?object=PWY-7221> |
| purine deoxyribonucleosides salvage | <http://humancyc.org/HUMAN/new-image?object=PWY-7224> |
| guanosine deoxyribonucleotides de novo biosynthesis | <http://humancyc.org/HUMAN/new-image?object=PWY-7226> |
| adenosine deoxyribonucleotides de novo biosynthesis | <http://humancyc.org/HUMAN/new-image?object=PWY-7227> |
| guanosine nucleotides de novo biosynthesis | <http://humancyc.org/HUMAN/new-image?object=PWY-7228> |
| iron-sulfur cluster biosynthesis | <http://humancyc.org/HUMAN/new-image?object=PWY-7250> |
| wybutosine biosynthesis | <http://humancyc.org/HUMAN/new-image?object=PWY-7283> |
| 7-(3-amino-3-carboxypropyl)-wyosine biosynthesis | <http://humancyc.org/HUMAN/new-image?object=PWY-7286> |
| progesterone biosynthesis | <http://humancyc.org/HUMAN/new-image?object=PWY-7299> |
| superpathway of steroid hormone biosynthesis | <http://humancyc.org/HUMAN/new-image?object=PWY-7305> |
| estradiol biosynthesis II | <http://humancyc.org/HUMAN/new-image?object=PWY-7306> |
| mRNA capping | <http://humancyc.org/HUMAN/new-image?object=PWY-7375-1> |
| mucin core 1 and core 2 O-glycosylation | <http://humancyc.org/HUMAN/new-image?object=PWY-7433> |
| terminal O-glycans residues modification | <http://humancyc.org/HUMAN/new-image?object=PWY-7434> |
| protein O-[N-acetyl]-glucosylation | <http://humancyc.org/HUMAN/new-image?object=PWY-7437> |
| allopregnanolone biosynthesis | <http://humancyc.org/HUMAN/new-image?object=PWY-7455> |
| phosphatidylserine biosynthesis I | <http://humancyc.org/HUMAN/new-image?object=PWY-7501> |
| purine nucleotides de novo biosynthesis | <http://humancyc.org/HUMAN/new-image?object=PWY-841> |
| mevalonate pathway | <http://humancyc.org/HUMAN/new-image?object=PWY-922> |
| pyruvate decarboxylation to acetyl CoA | <http://humancyc.org/HUMAN/new-image?object=PYRUVDEHYD-PWY> |
| adenosine nucleotides degradation | <http://humancyc.org/HUMAN/new-image?object=SALVADEHYPOX-PWY> |
| S-adenosyl-L-methionine biosynthesis | <http://humancyc.org/HUMAN/new-image?object=SAM-PWY> |
| L-serine degradation | <http://humancyc.org/HUMAN/new-image?object=SERDEG-PWY> |
| serine and glycine biosynthesis | <http://humancyc.org/HUMAN/new-image?object=SER-GLYSYN-PWY-1> |
| serine biosynthesis (phosphorylated route) | <http://humancyc.org/HUMAN/new-image?object=SERSYN-PWY> |
| thioredoxin pathway | <http://humancyc.org/HUMAN/new-image?object=THIOREDOX-PWY> |
| triacylglycerol biosynthesis | <http://humancyc.org/HUMAN/new-image?object=TRIGLSYN-PWY> |
| tRNA charging | <http://humancyc.org/HUMAN/new-image?object=TRNA-CHARGING-PWY> |
| tryptophan degradation | <http://humancyc.org/HUMAN/new-image?object=TRYPTOPHAN-DEGRADATION-1> |
| tyrosine degradation | <http://humancyc.org/HUMAN/new-image?object=TYRFUMCAT-PWY> |
| UDP-N-acetyl-D-glucosamine biosynthesis II | <http://humancyc.org/HUMAN/new-image?object=UDPNACETYLGALSYN-PWY> |
| valine degradation | <http://humancyc.org/HUMAN/new-image?object=VALDEG-PWY> |
