# Supplementary material for: Molecular pathway activation features linked with transition from normal skin to primary and metastatic melanomas in human
Source: Oncotarget. 2015 Nov 26;7(1):656–70. doi: 10.18632/oncotarget.6394 (PMC4808024; doi:10.18632/oncotarget.6394)

Clusters, determined using WGCNA.

Cluster #1

Correlation heatmap

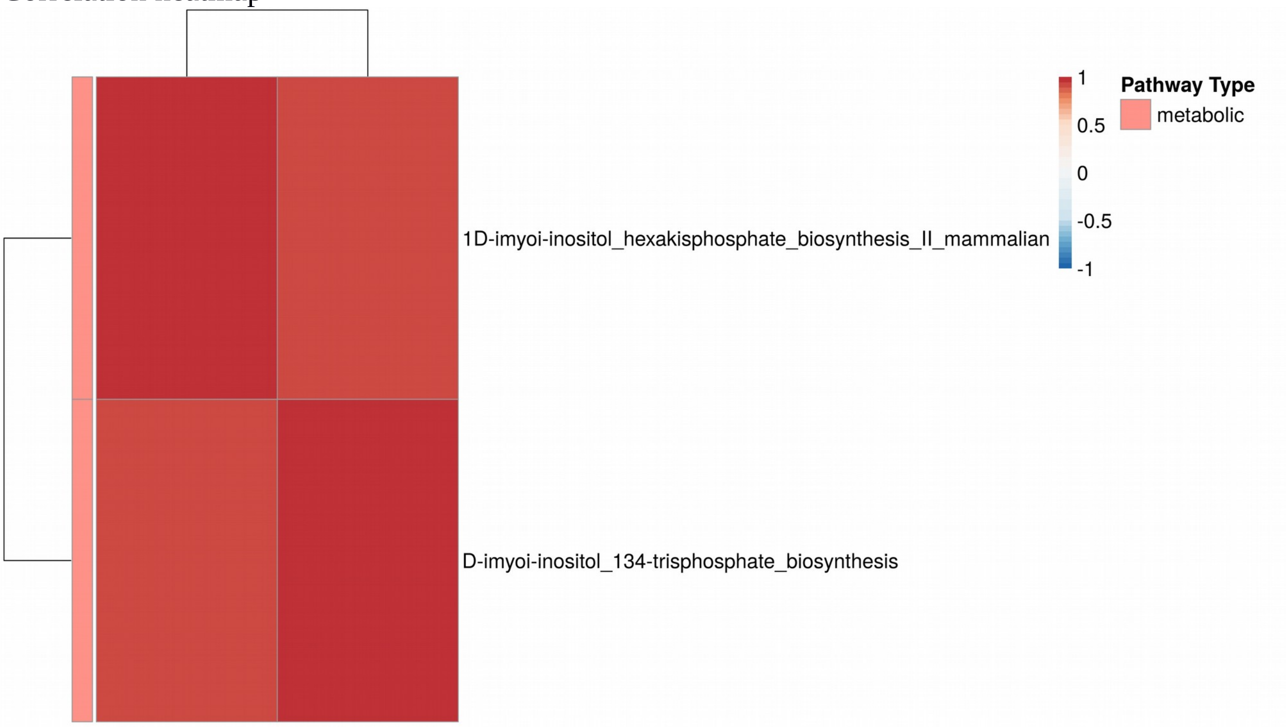

Jaccard index heatmap

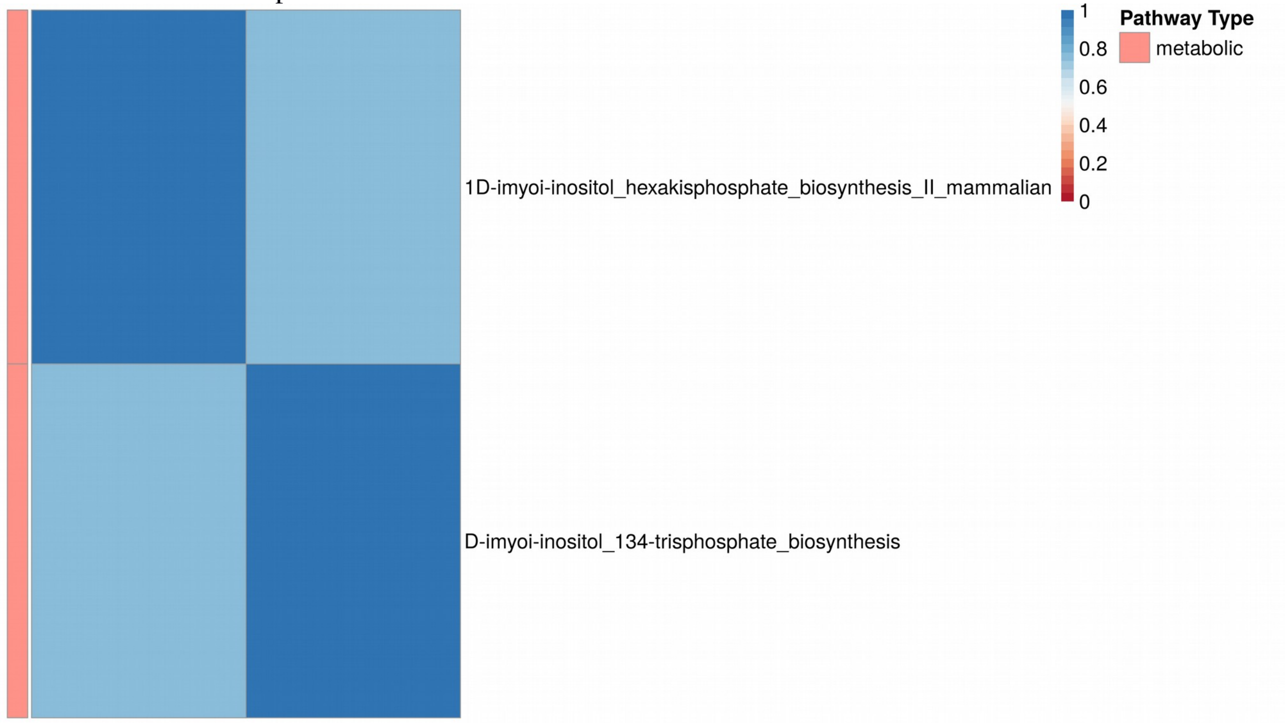

Correlation table

Cluster #2

Correlation heatmap

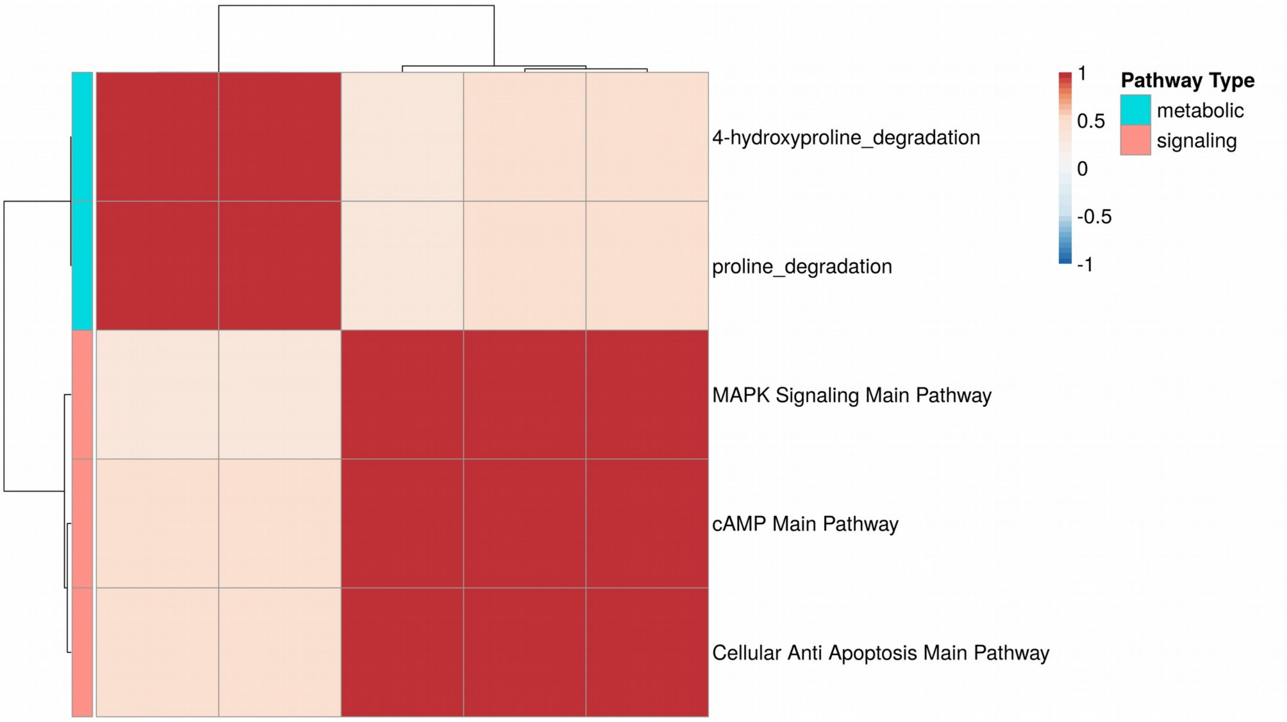

Jaccard index heatmap

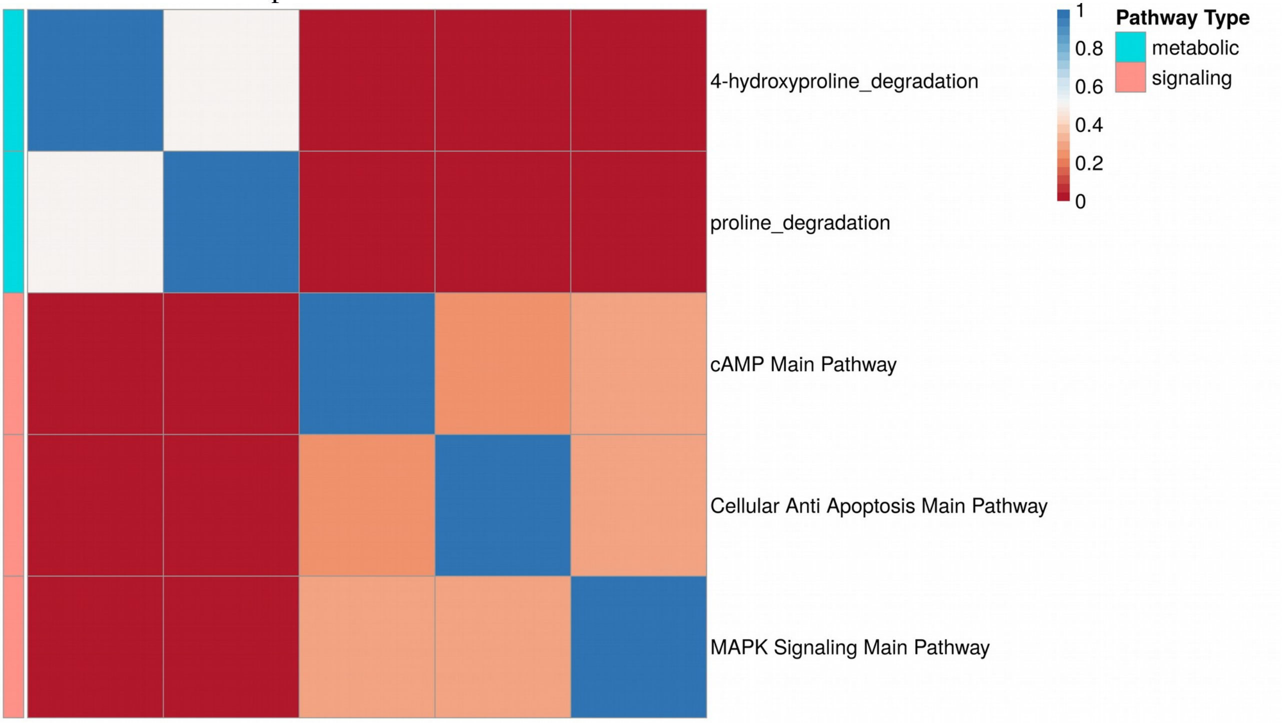

Correlation table

Cluster #3

Correlation heatmap

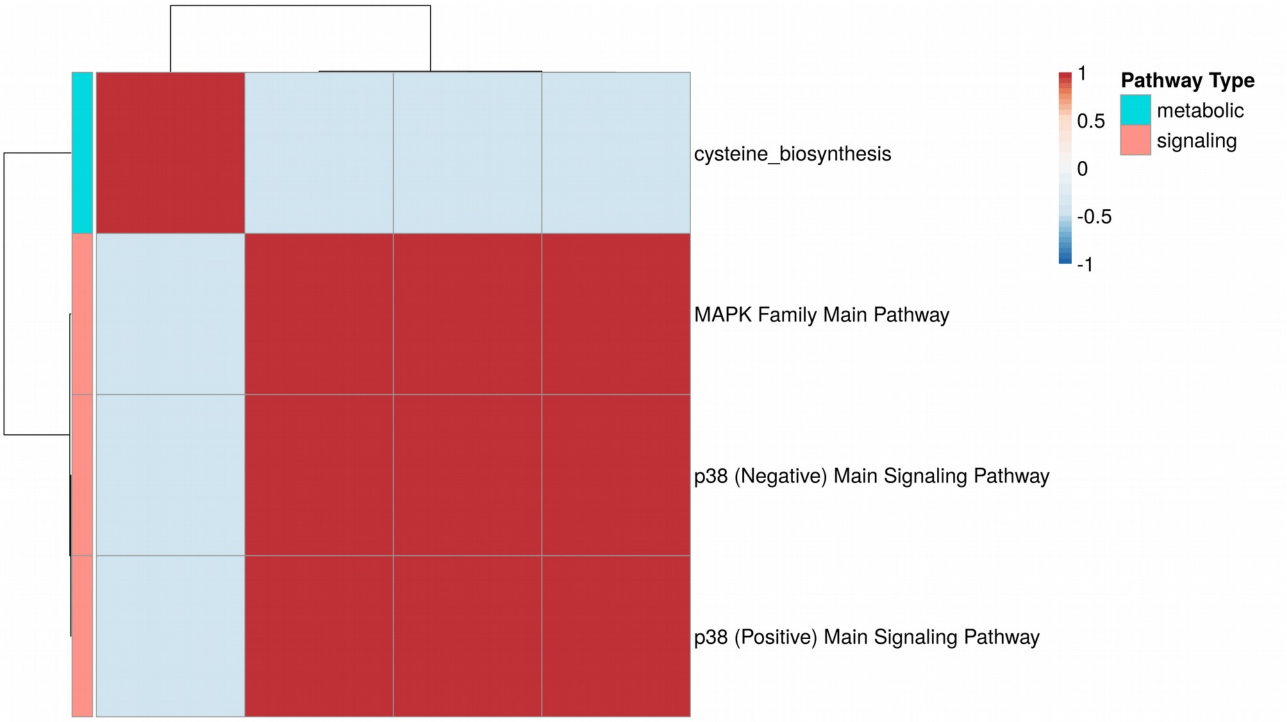

Jaccard index heatmap

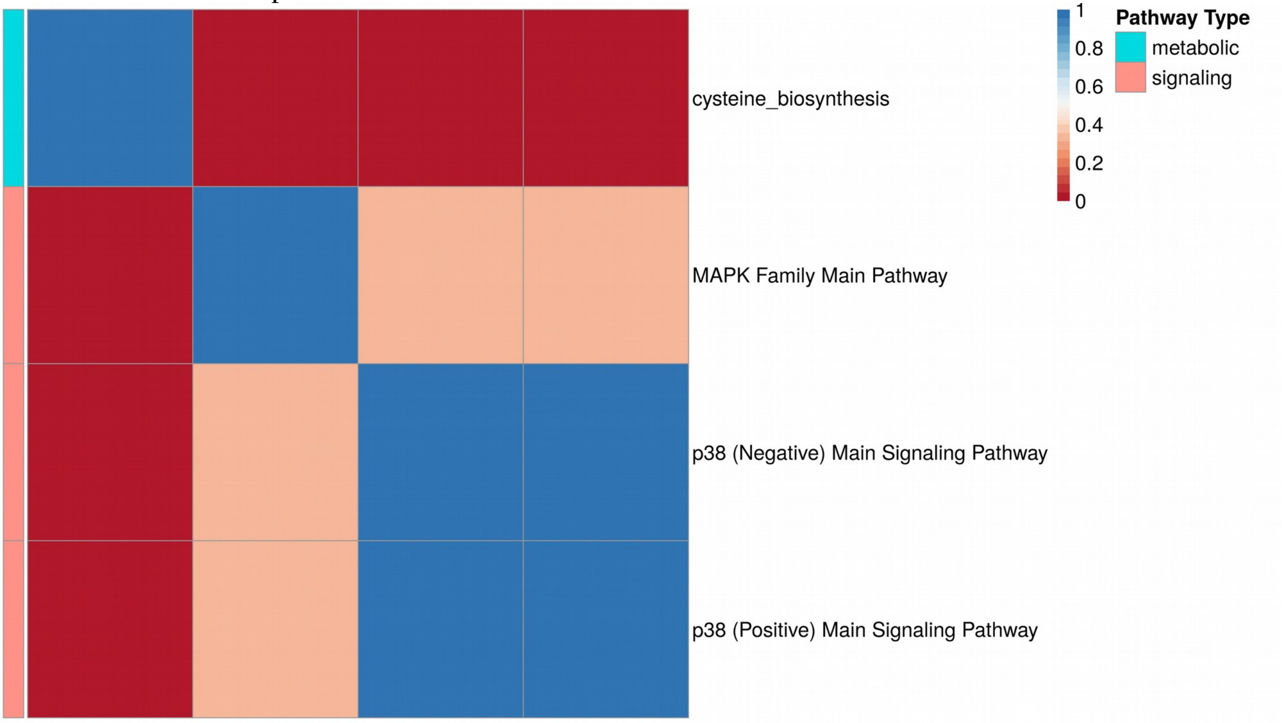

Cluster #4

Correlation heatmap

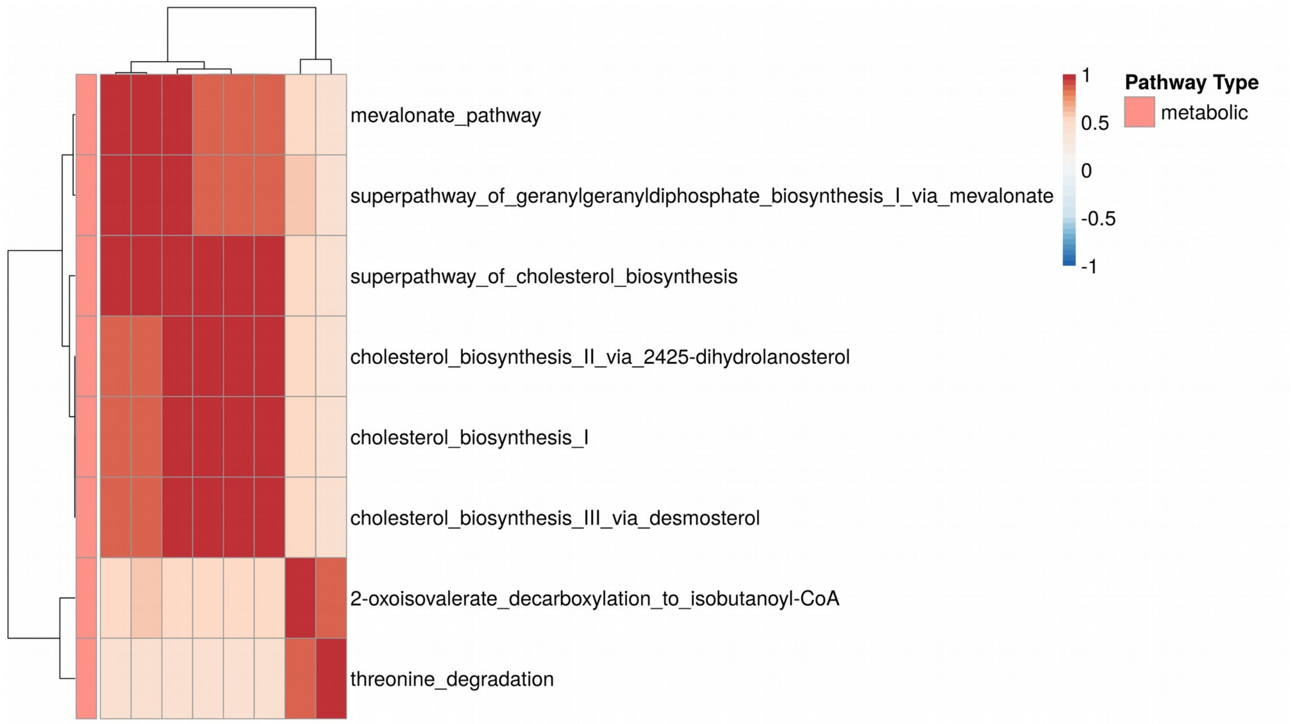

Jaccard index heatmap

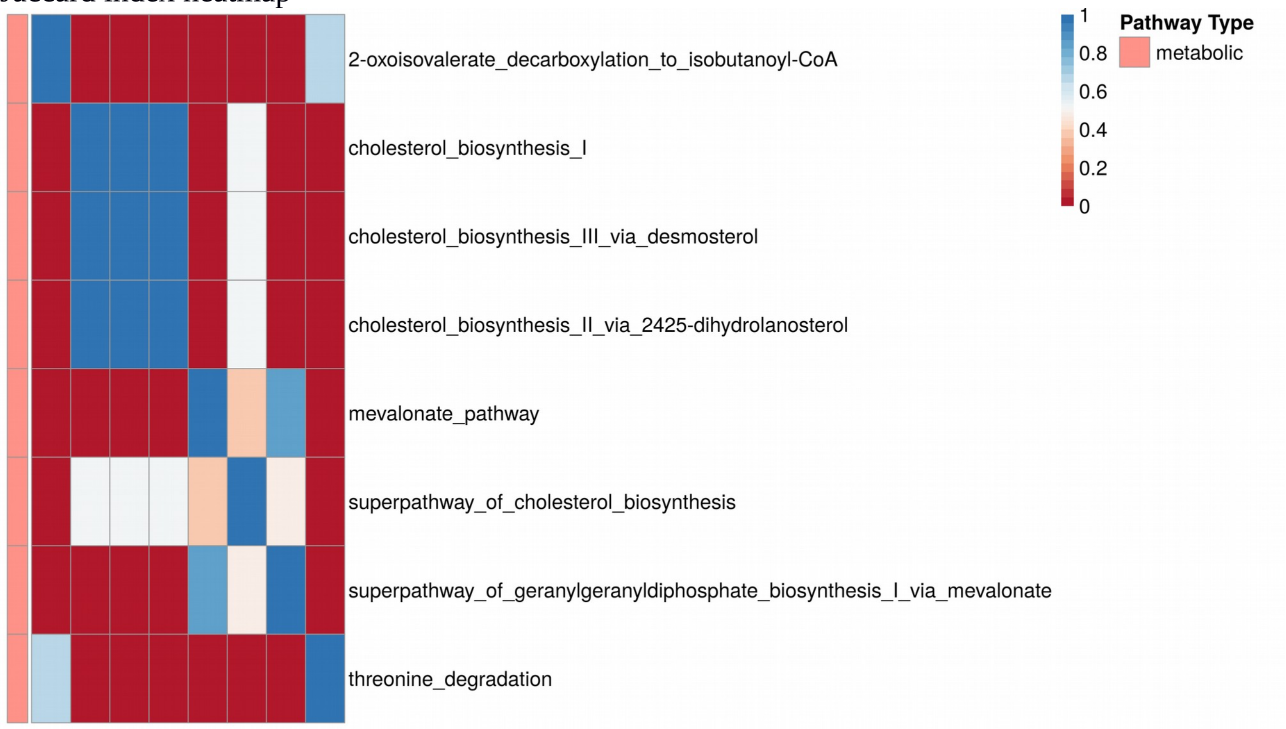

Cluster #5
Corelation heatmap

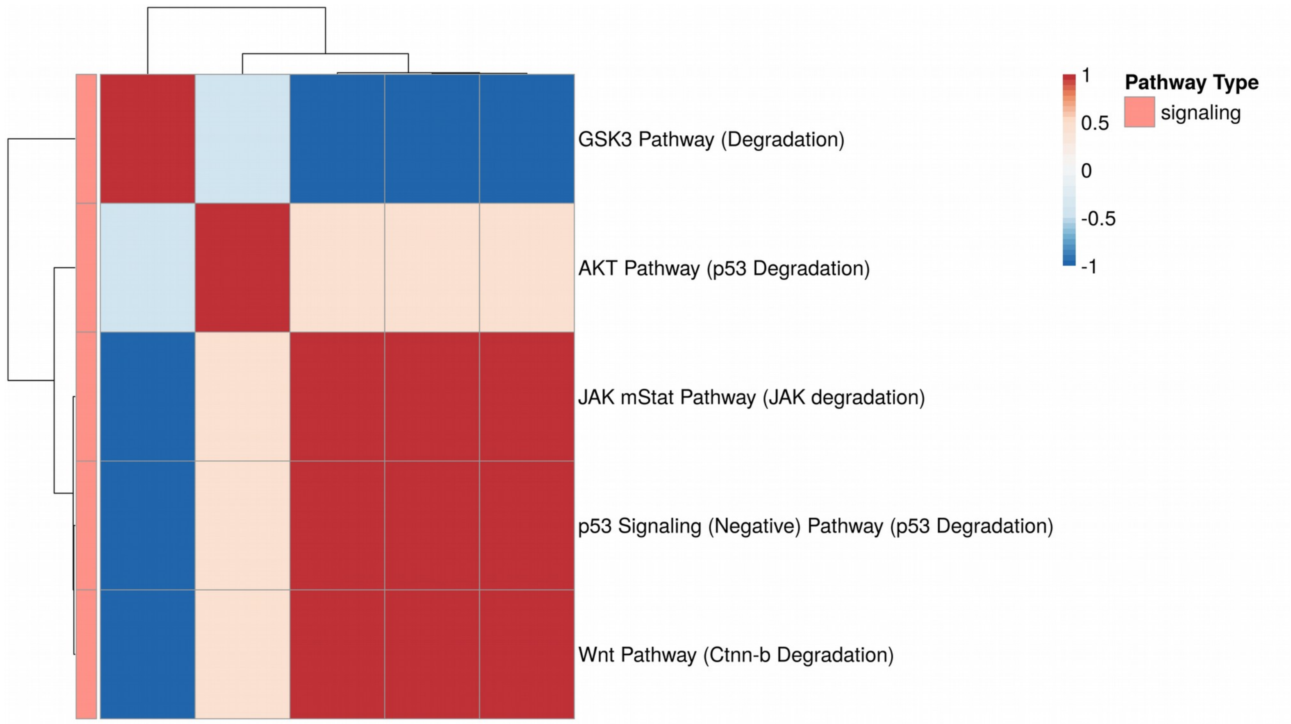

Jaccard index heatmap

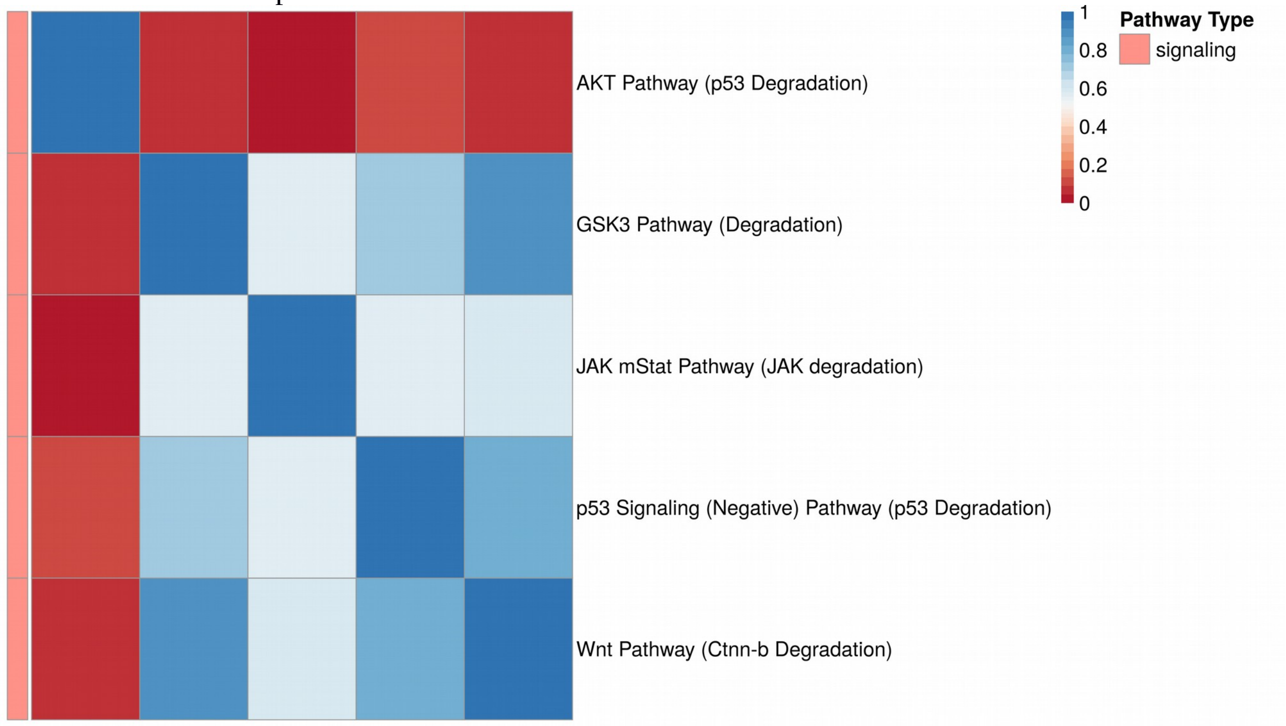

Cluster #6

Corelation heatmap

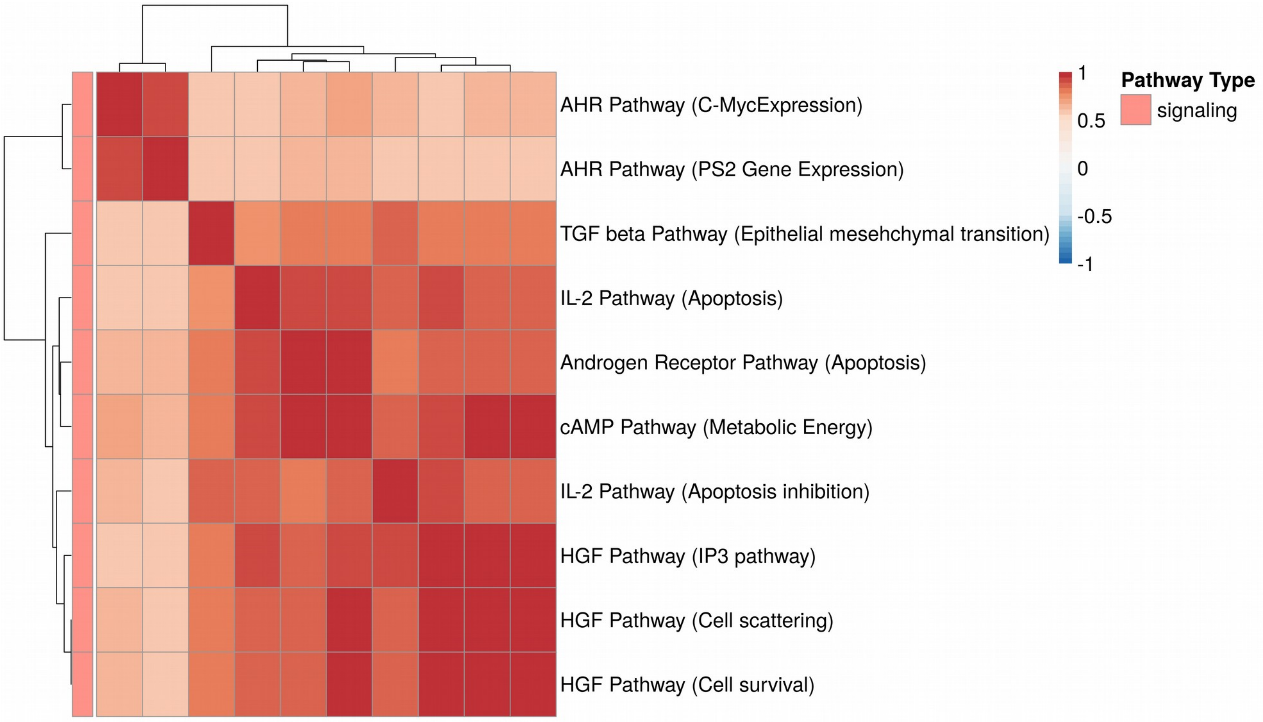

Jaccard index heatmap

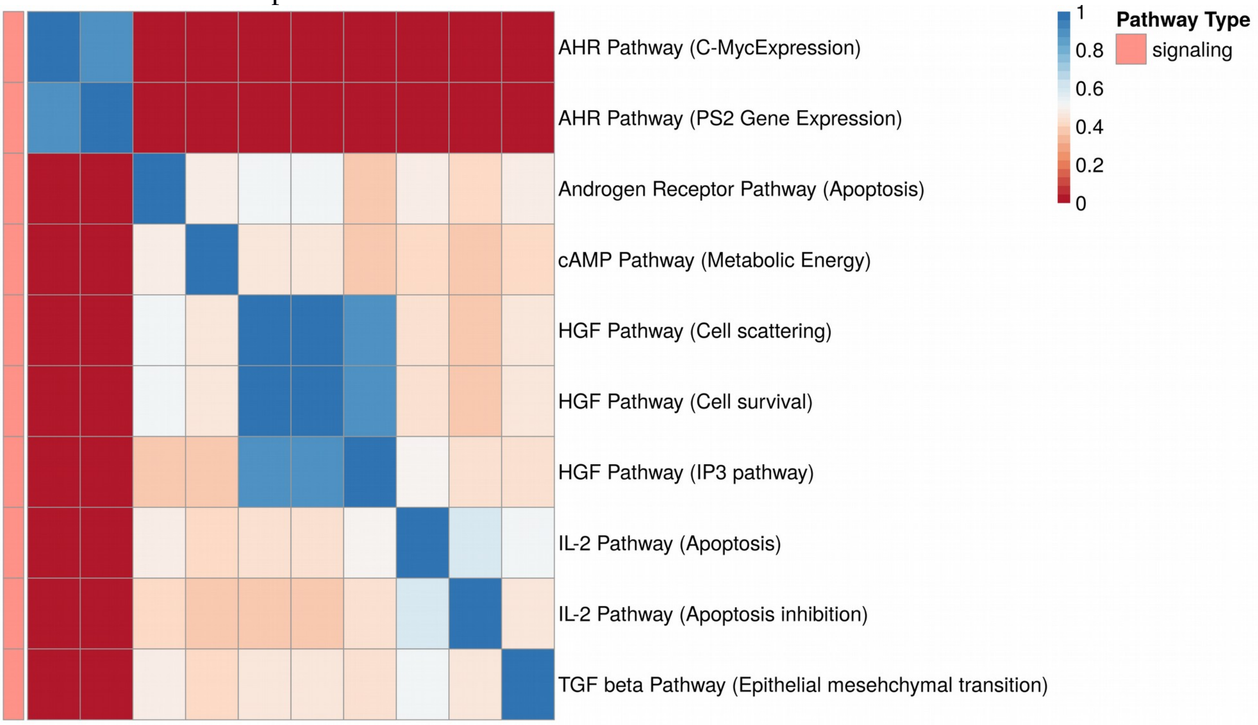

Cluster #7

Corelation heatmap

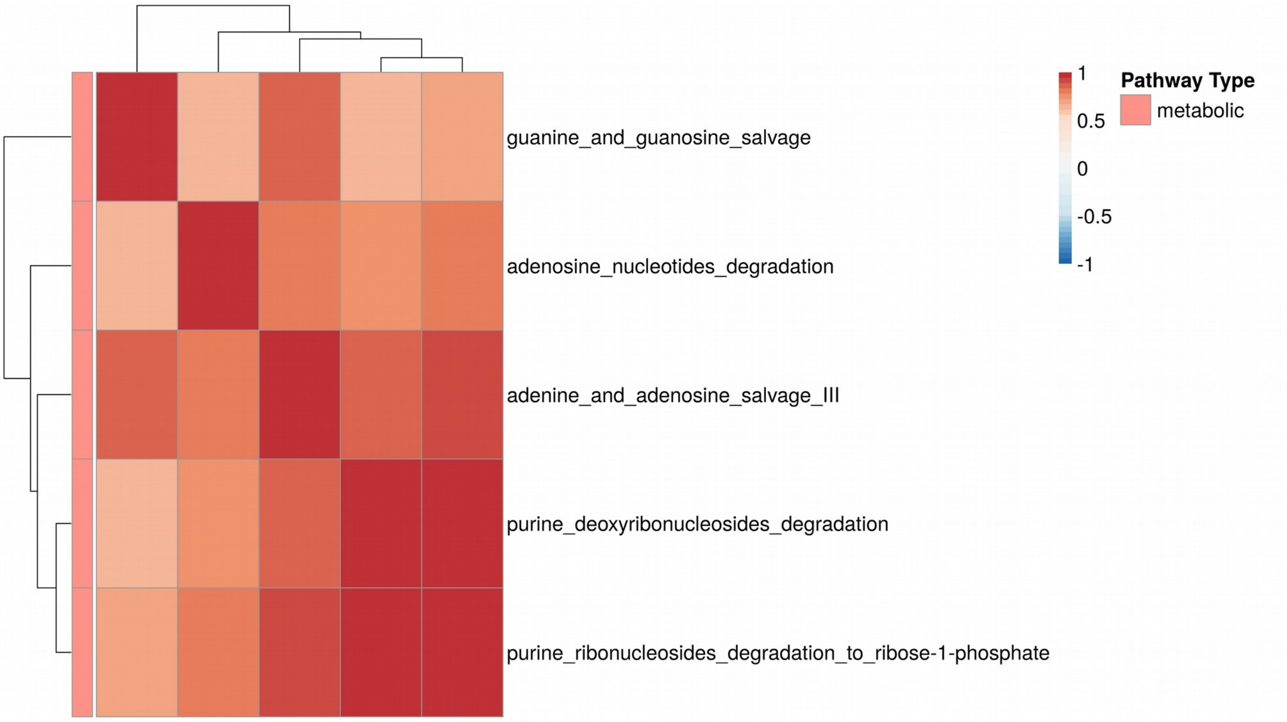

Jaccard index heatmap

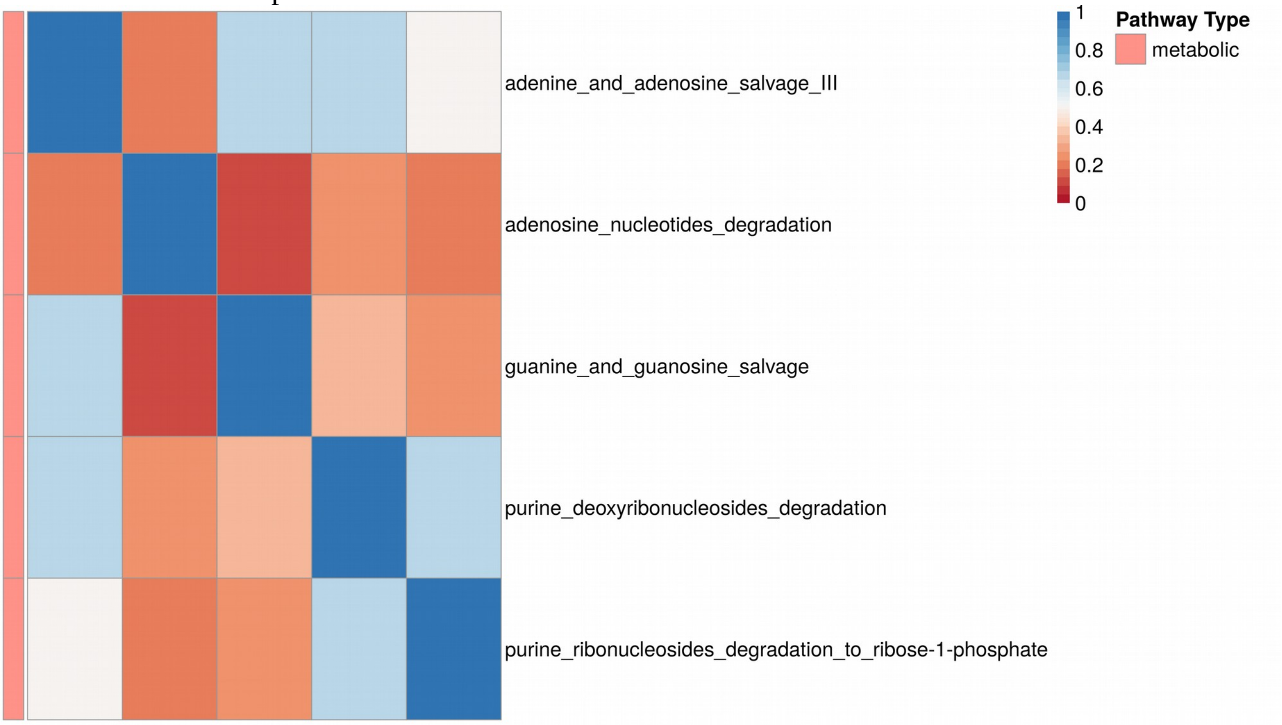

Cluster #8

Corelation heatmap

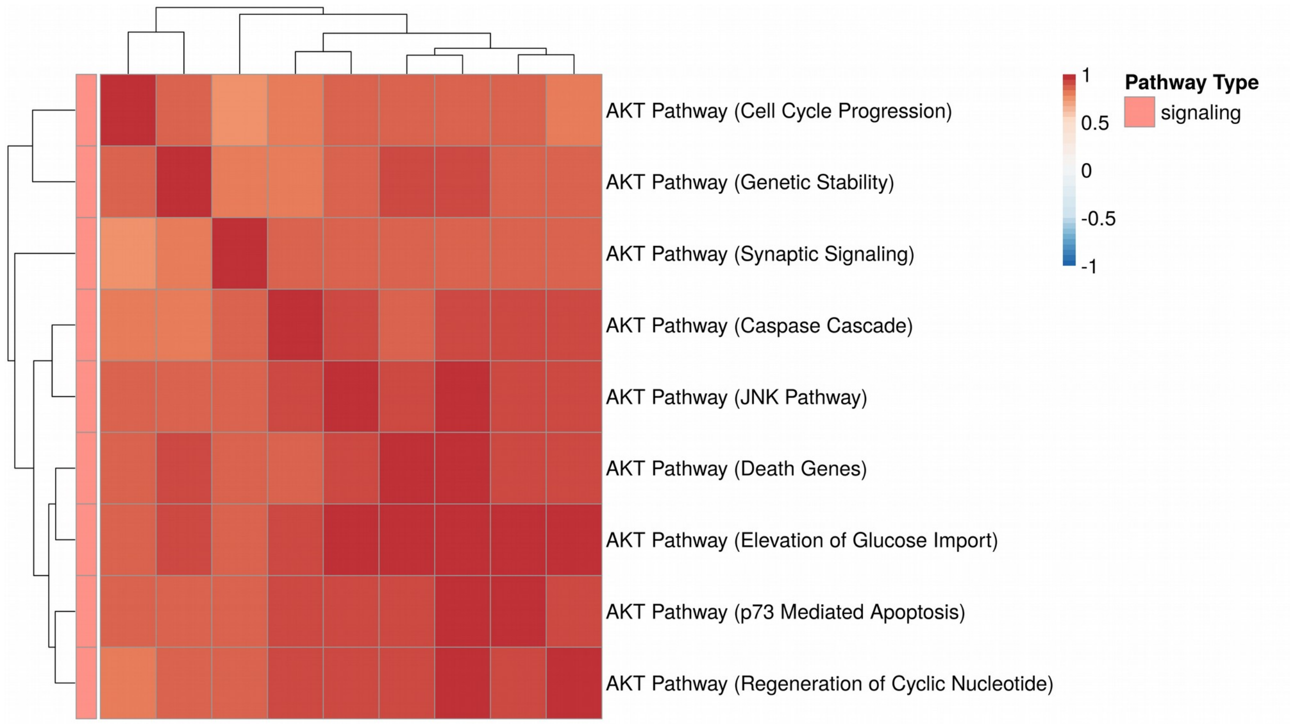

Jaccard index heatmap

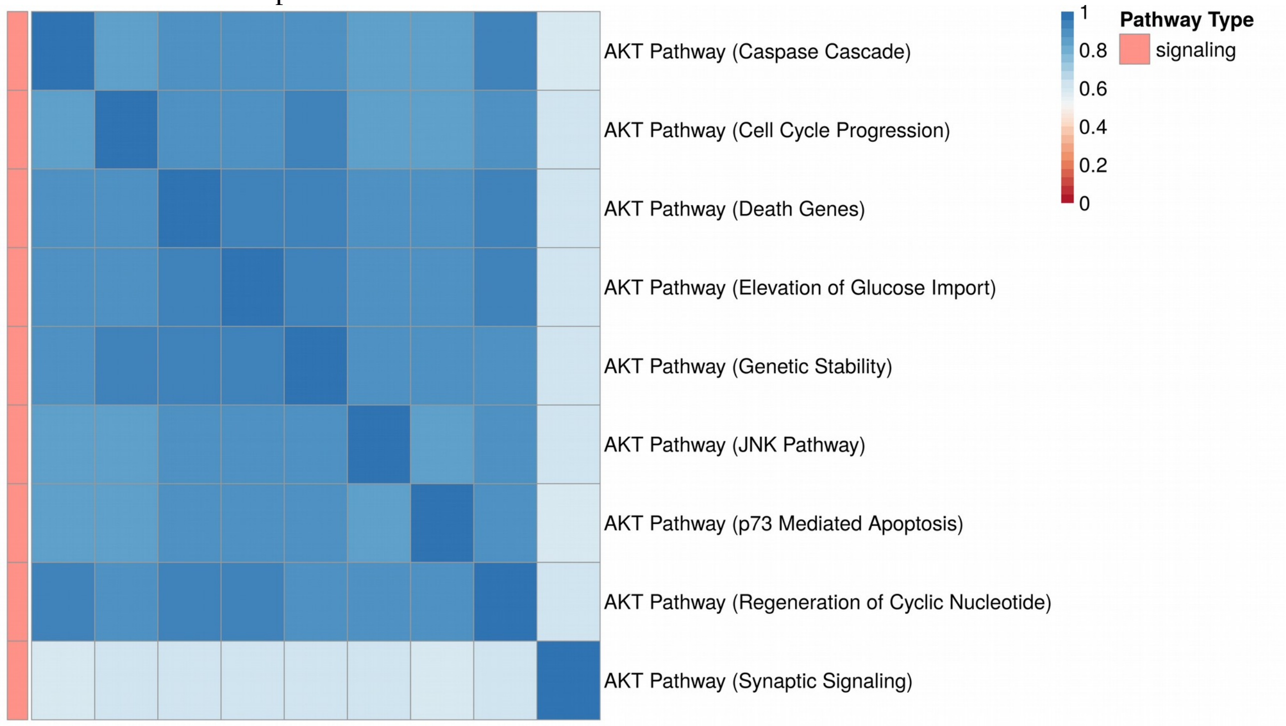

Cluster #9

Corelation heatmap

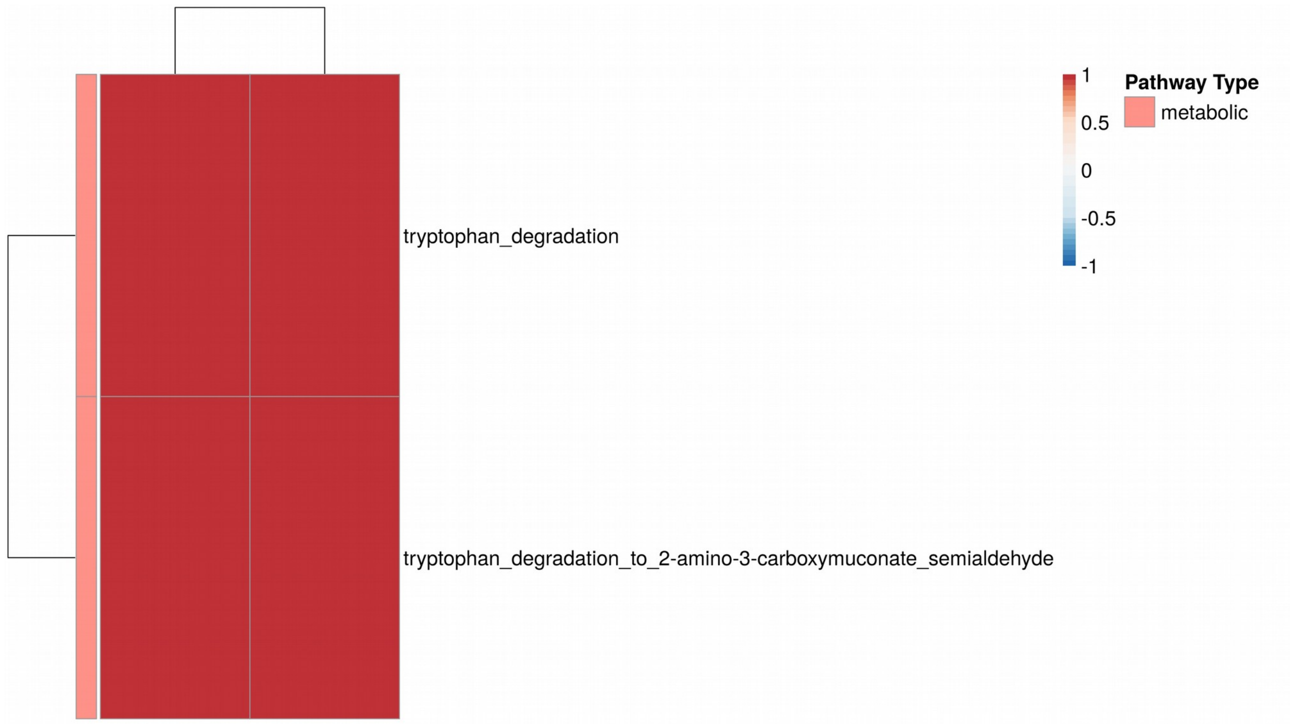

Jaccard index heatmap

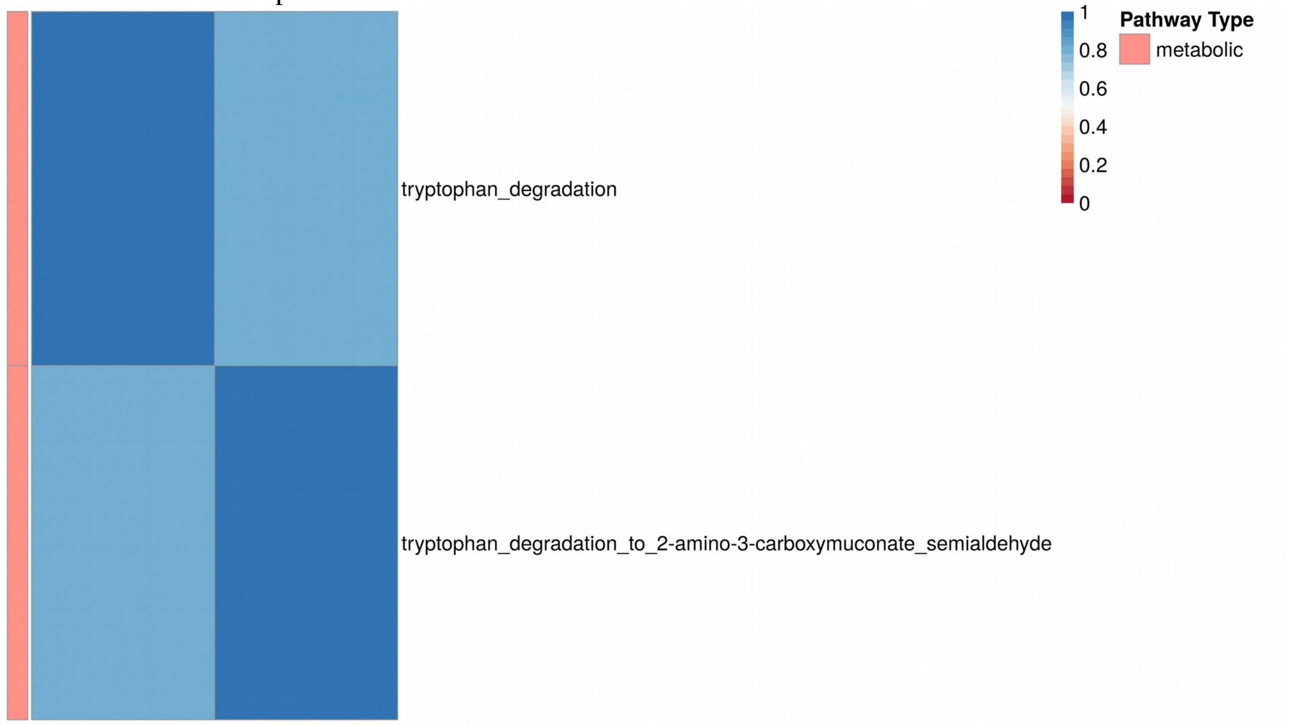

Cluster #10

Corelation heatmap

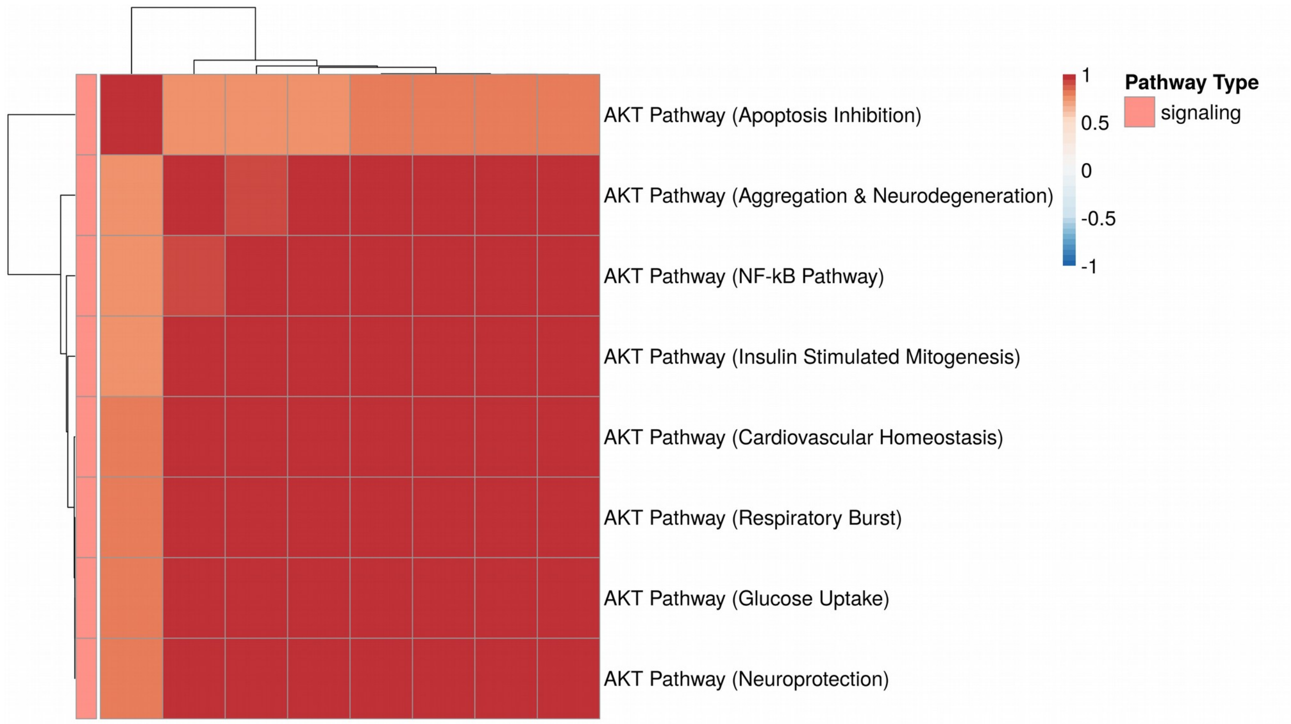

Jaccard index heatmap

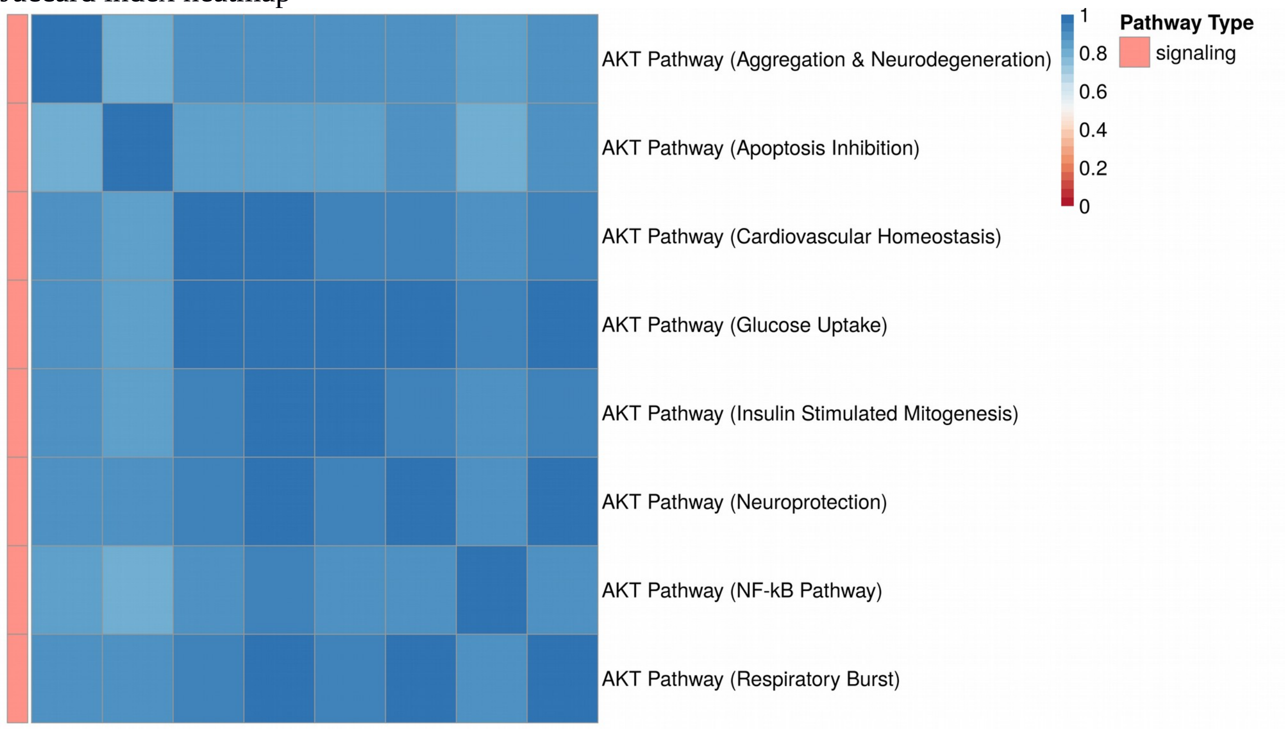

Cluster #11

Corelation heatmap

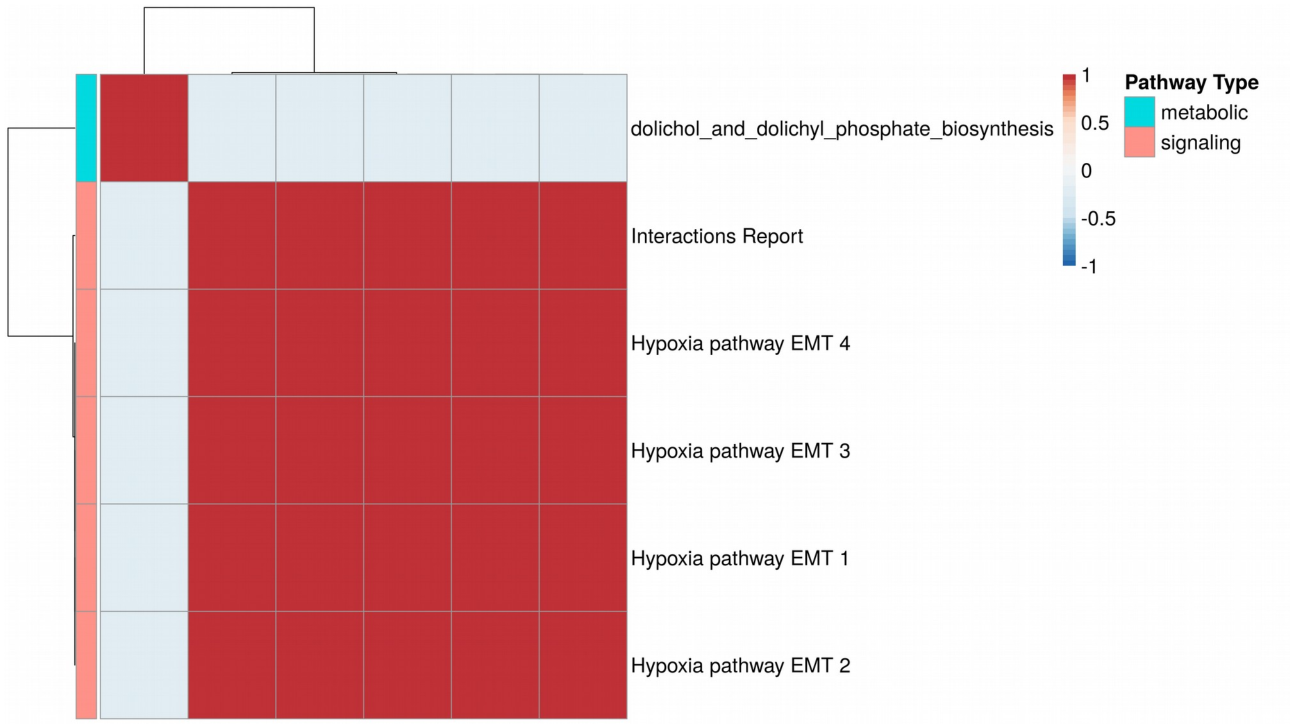

Jaccard index heatmap

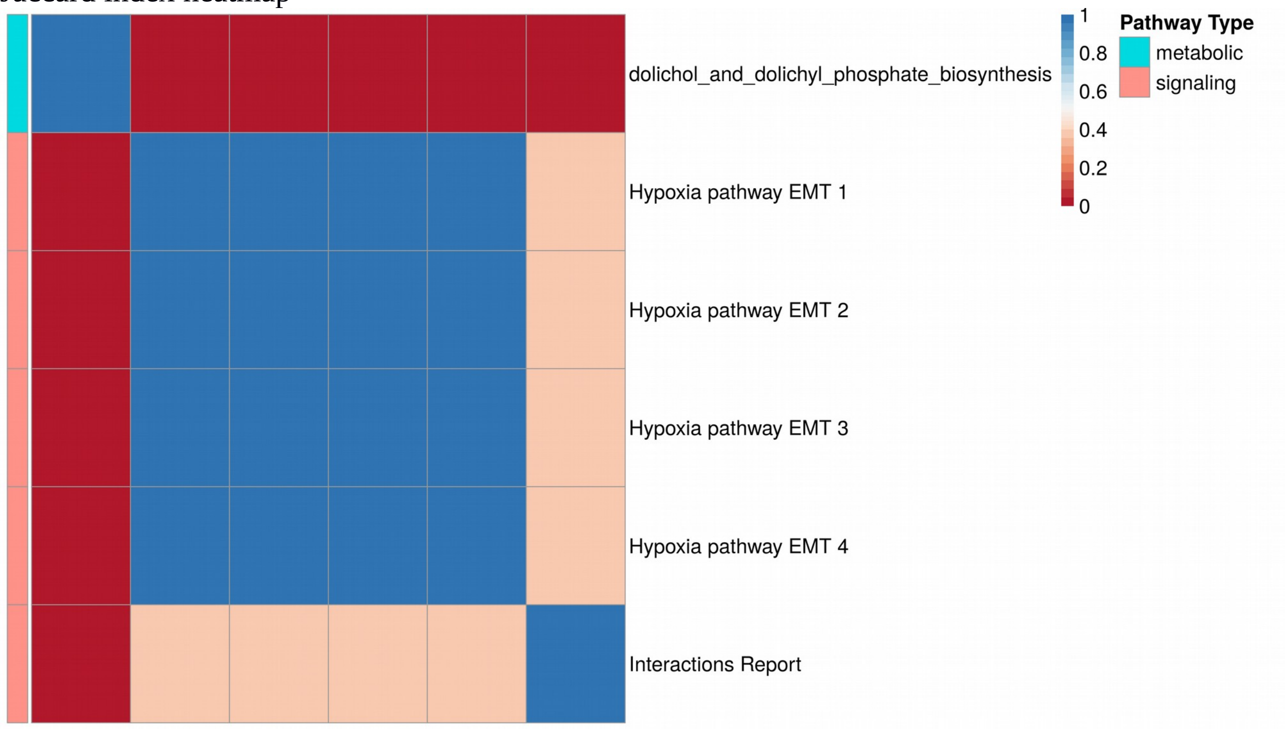

Cluster #12

Corelation heatmap

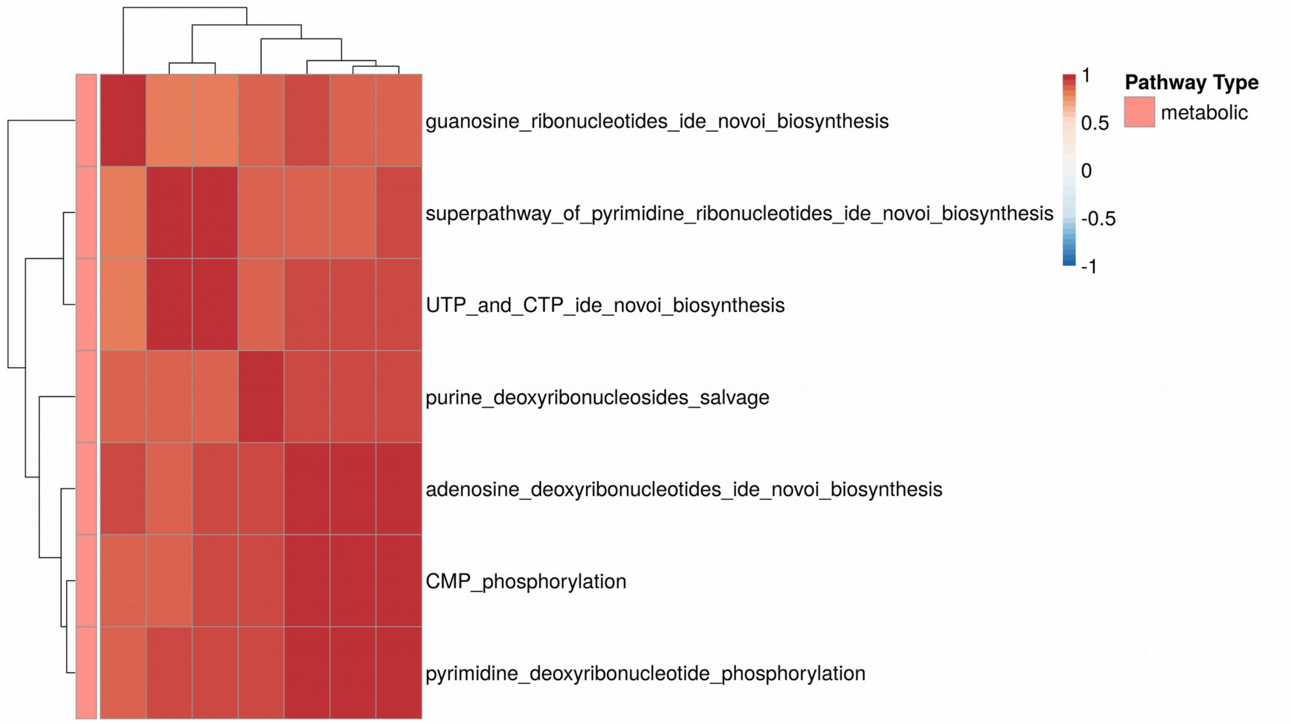

Jaccard index heatmap

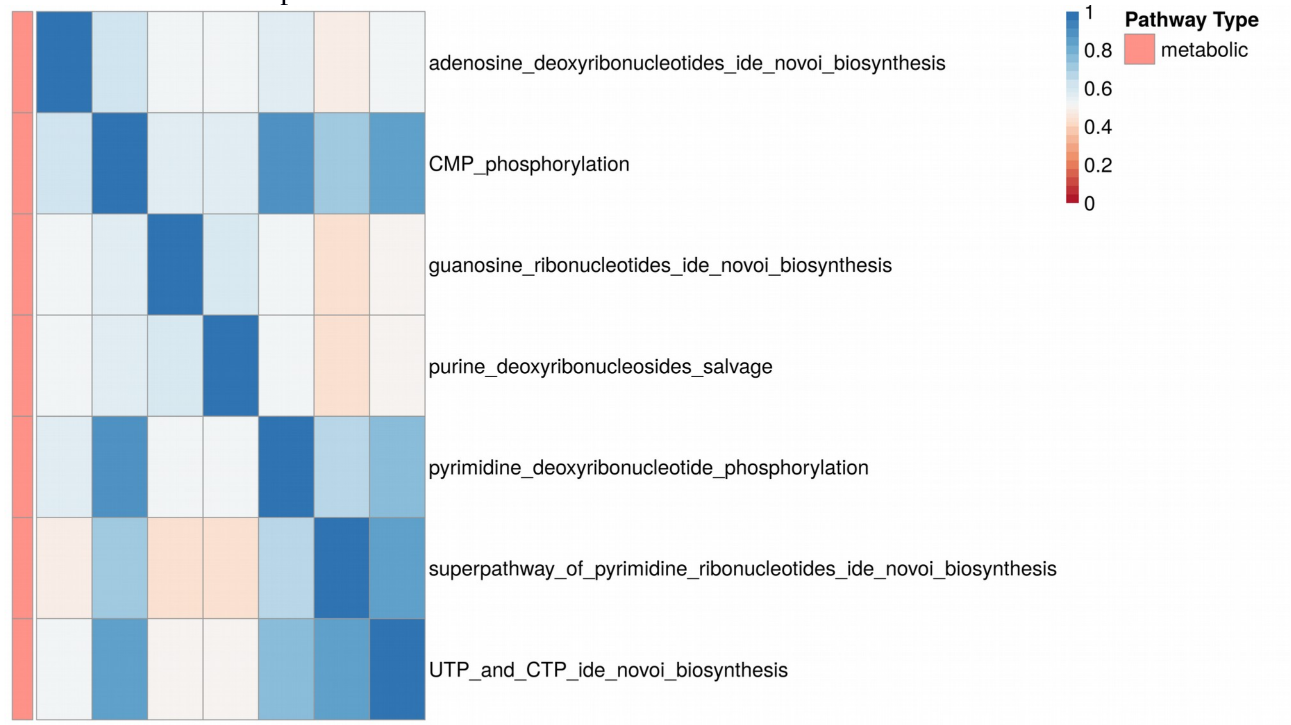

Cluster #13

Corelation heatmap

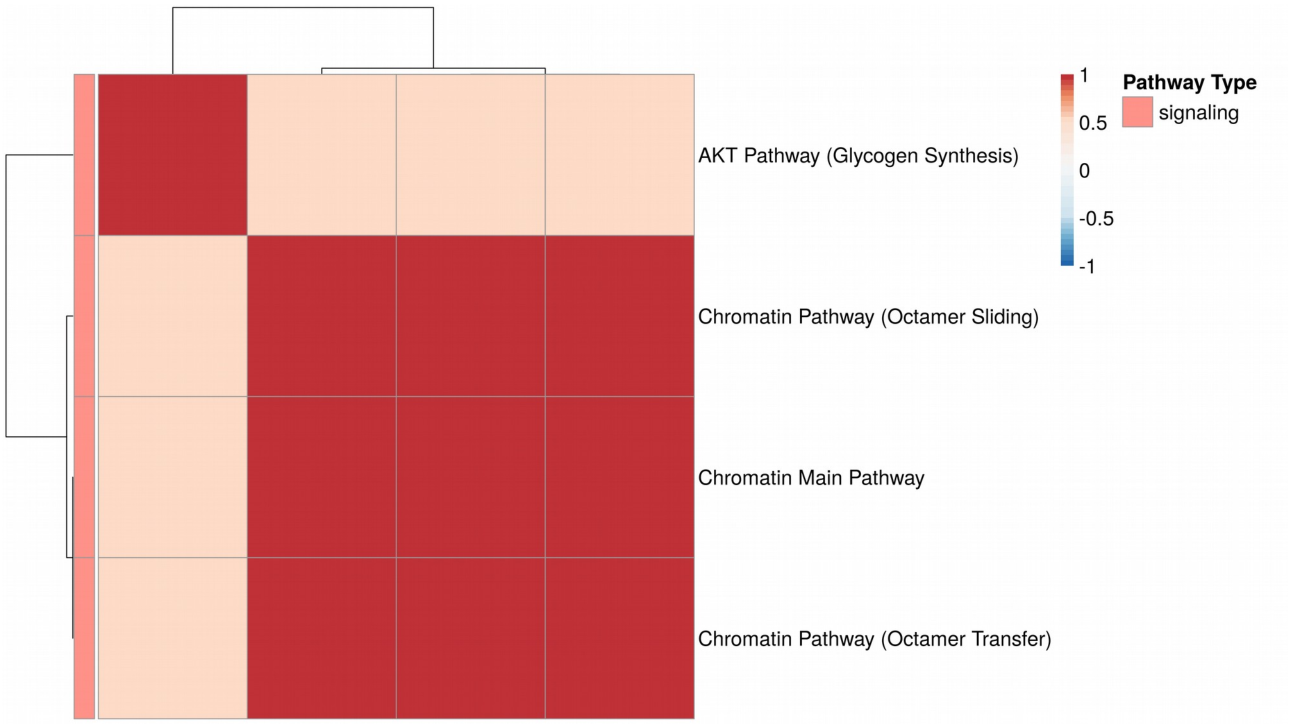

Jaccard index heatmap

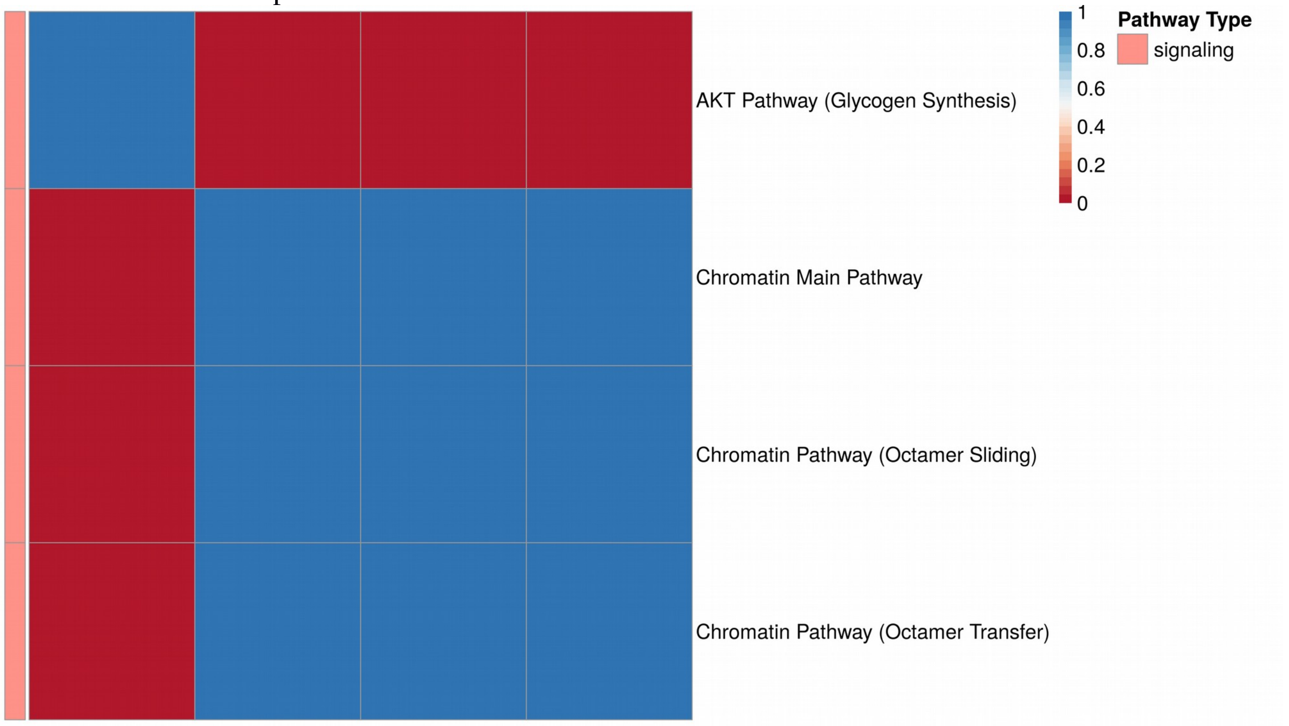

Cluster #14
Corelation heatmap

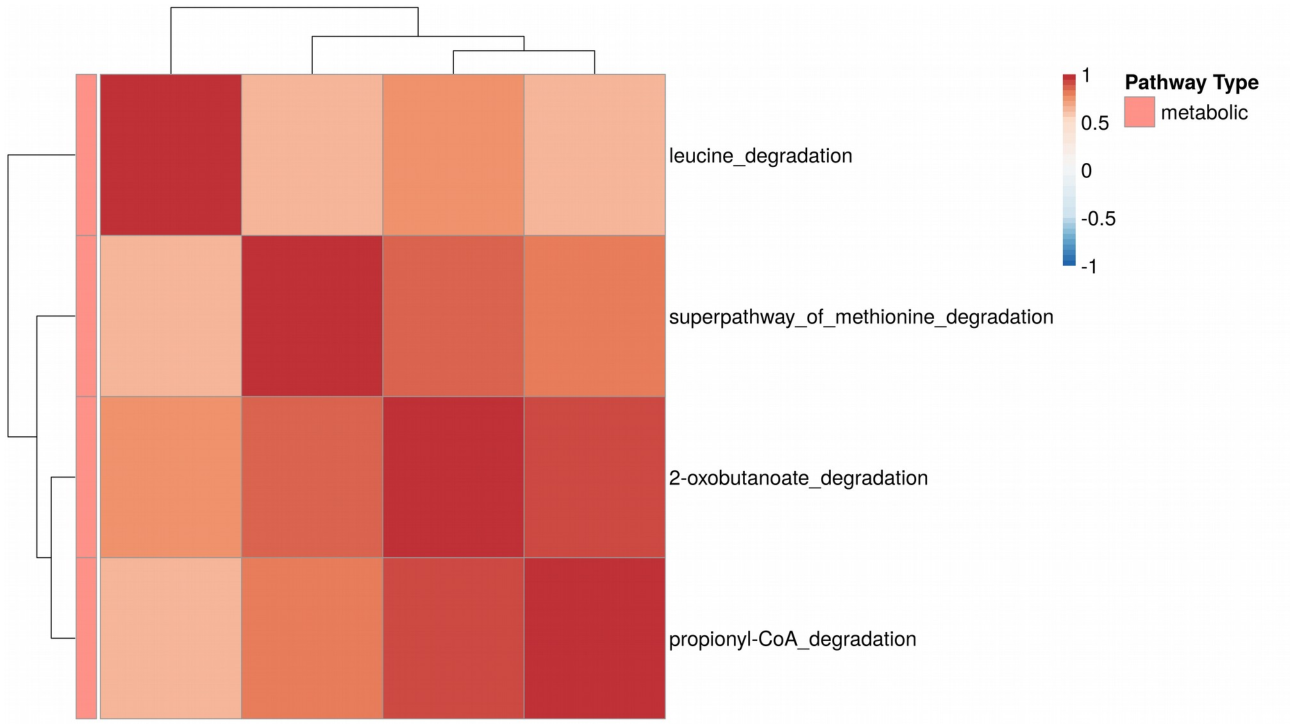

Jaccard index heatmap

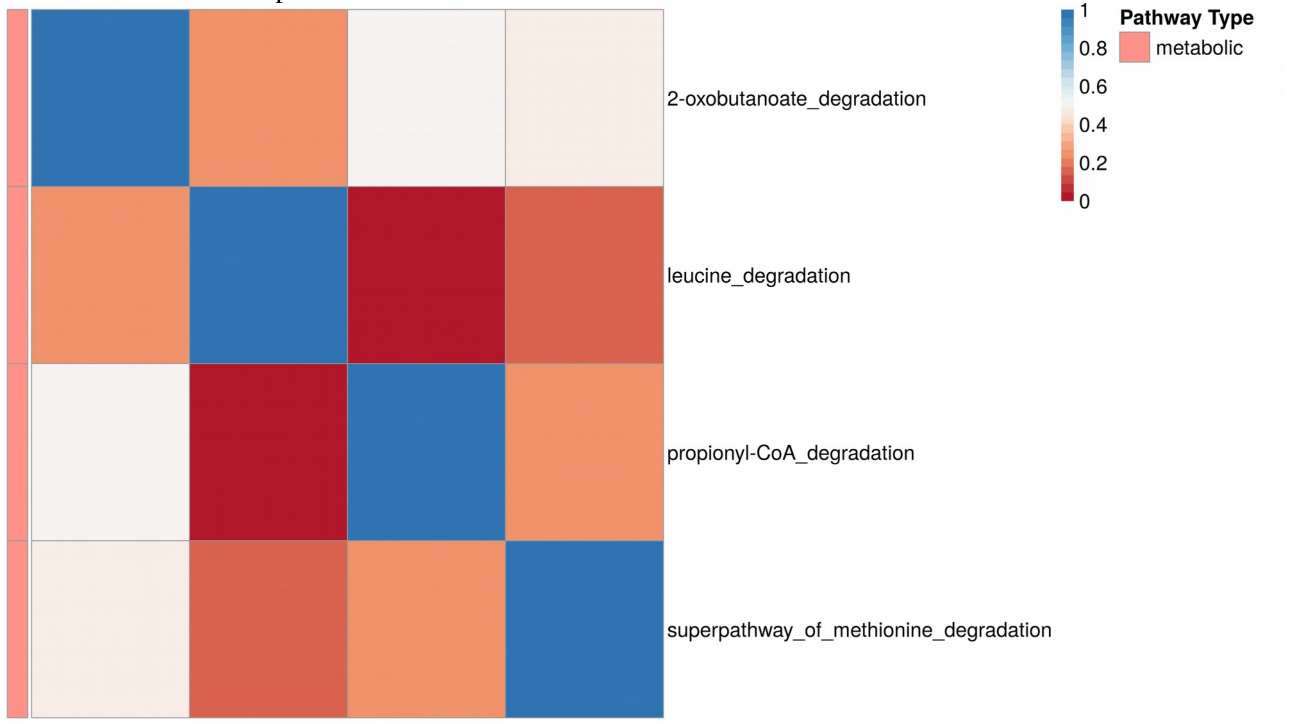

Supplement: Supplementary file 6 [file oncotarget-07-0656-s006.pdf]
